# Steric Gating Contributes to the Preferential Ubiquitin Charging of BIRC6 by UBA6 versus UBA1

**DOI:** 10.1038/s41467-026-75898-6

**Published:** 2026-08-29

**Authors:** Zebin Tong, Rujing Yuan, Xiangwei Wu, Hongyi Cai, Ziyu Xu, Lei Liu, Huasong Ai

**Affiliations:** 1 School of Pharmaceutical Sciences, Shanghai Key Laboratory for Antibody-Drug Conjugates with Innovative Target, Center for Chemical Glycobiology-Zhangjiang Institute for Advanced Study, Shanghai Frontiers Science Center of Drug Target Identification and Delivery, Research Center for Chemical Biology of Post-Translational Modifications, Shanghai Jiao Tong University, Shanghai, China; 2 New Cornerstone Science Laboratory, Tsinghua-Peking Joint Center for Life Sciences, Ministry of Education Key Laboratory of Bioorganic Phosphorus Chemistry and Chemical Biology, Center for Synthetic and Systems Biology, Department of Chemistry, Tsinghua University, Beijing, 100084 China; 3 Present Address: Department of Molecular Machines and Signaling, Max Planck Institute of Biochemistry, Martinsried, Germany

**Keywords:** Enzyme mechanisms, Enzyme mechanisms, Cryoelectron microscopy, Ubiquitylation, Multienzyme complexes

## Abstract

The giant E2–E3 chimera BIRC6 specifically engages ubiquitin-activating E1 enzyme UBA6, but not UBA1, enabling UBA6 to transfer activated ubiquitin to BIRC6, which then ubiquitinates downstream substrates such as caspases to regulate apoptosis. However, the molecular mechanism that underlies UBA6-mediated specific engagement of BIRC6 remains elusive. Here we use a Ub^Dha^ probe to trap the transient E1–E2 transthioesterification intermediate and determine cryo-EM structures of singly and doubly loaded UBA6-BIRC6^UBC^ complexes at 3.3 and 3.4 Å resolution. Structural analysis reveals that an insertion loop (residues 4649–4653) in BIRC6 and the gate helix of UBA6 play a key role in the specific BIRC6-UBA6 pairing. Unlike UBA1, UBA6 avoids steric clash through a ~ 30° rotation and a 19 Å displacement of its gate helix, creating a compatible cavity for the insertion loop. Biochemical validation confirms that truncation of the BIRC6 insertion loop rescues UBA1‑mediated charging, and swapping the UBA6 gate helix into UBA1 confers activity toward BIRC6^UBC^. Our study provides structural insights into E1–E2 pairing specificity and advances the mechanistic understanding of apoptotic signaling.

## Abstract (web summary)

The giant E2–E3 chimera BIRC6 specifically engages Ub-activating E1 enzyme UBA6, but not UBA1. Here, the authors reveal the structural basis of this selectivity, showing that a unique gate helix in UBA6 creates a compatible pocket for a key insertion loop in BIRC6, while UBA1 causes a steric clash.

## Introduction

Ubiquitin (Ub) and ubiquitin-like proteins (UBLs) modify target proteins to alter their structures, localizations, and interactions, thereby regulating a broad range of cellular functions, including protein homeostasis, cell division, and development^1–3^. The covalent attachment of Ub/UBLs to substrates relies on a sequential enzymatic cascade involving E1 activating enzymes, E2 conjugating enzymes, and E3 ligases^4–6^. During E1 activation, the C-terminus of Ub/UBLs is first adenylated in the presence of ATP, followed by thioester bond formation with the catalytic cysteine of E1. E1 then recruits an E2 conjugating enzyme and transfers the Ub/UBL moiety to the catalytic cysteine of the E2, thereby generating an E2~Ub/UBL thioester intermediate (where “~” denotes a thioester bond), a process known as E1–E2 transthioesterification. Finally, E3 ligases transfer Ub/UBLs from the E2 to either their own catalytic cysteine (HECT family) or directly to the target substrate (RING family), depending on their classification^7–9^.

For most UBL systems, conjugation is predominantly initiated by a single dedicated E1 activating enzyme^9^. The ubiquitin system is a notable exception: in 2007, a second ubiquitin-activating enzyme, UBA6, was identified in humans in addition to the canonical E1 enzyme UBA1^10^. UBA6 shares approximately 40% sequence similarity with UBA1^11^. These two E1s engage over 30 different E2 conjugating enzymes, yet they exhibit distinct E2 selectivity^8^. For example, UBA1 specifically engages E2s such as UBE2H, UBE2R2, and UBE2K, whereas UBA6 is specific to E2s including UBE2Z and BIRC6. Additionally, a subset of E2s, such as UbcH5 family, UBE2G2 and UBE2S, can be charged by both UBA1 and UBA6^10^. This differential E2 preference allows the two E1s to direct ubiquitin to distinct subsets of E3 ligases, thereby mediating the modification of specific substrates and triggering diverse downstream cellular functions, including protein degradation, DNA repair, and immune signaling^12^. Previous structural studies of UBA1 in complex with various E2s have delineated the mechanisms underlying ubiquitin adenylation, UBA1~Ub thioester bond formation, and UBA1-E2 transthioesterification^13–21^. In contrast, the molecular basis governing UBA6’s selective recognition of E2 partners has remained unclear, limiting our understanding of E1–E2 pairing specificity.

BIRC6 is a giant 4857-amino acid E2–E3 hybrid enzyme and the only essential inhibitor of apoptosis (IAP)^22,23^. Apoptosis is carried out by a family of cysteine proteases known as caspases, which initiate and execute programmed cell death in response to developmental cues or cellular stress. IAP proteins function by directly restricting these caspases, thereby inhibiting apoptosis. BIRC6’s C-terminus contains a ubiquitin-conjugating (UBC) domain, which is a hallmark of the E2 ubiquitin-conjugating enzyme family^8,24^, while its N-terminal baculoviral IAP repeat (BIR) domains recognize specific degrons on substrates, conferring E3 ligase-like activity for direct substrate recruitment^25^. In the apoptosis pathway, BIRC6 functions exclusively with UBA6, but not UBA1^26–28^, enabling it to ubiquitinate substrates such as caspases-3, -7, -9, and HTRA2, thereby suppressing apoptosis^29,30^.

Recent structural studies have reported the structures of apo BIRC6 and BIRC6 in complex with various client proteins^26–28,31^. These structures revealed that BIRC6 forms an unusual, massive head-to-tail dimer, thereby assembling into a U-shaped complex with a molecular mass of approximately 1.2 MDa. This architecture features a central cavity flanked by key functional domains such as the UBC and BIR domains. The central cavity serves as a critical binding site for both substrates (including caspases-3, -7, -9, and HTRA2) and the inhibitory protein SMAC (second mitochondria-derived activator of caspases). Although these studies have elucidated the role of BIRC6 as an E3 ligase in substrate recognition and recruitment, the structural and mechanistic basis underlying its specificity for UBA6 (when acting as an E2 enzyme) and its lack of reactivity with UBA1 remains unknown.

Here, we show that the UBA6-BIRC6 specificity is governed by steric compatibility between an insertion loop in BIRC6 and the gate helix of UBA6. By employing an activity‑based Ub^Dha^ probe, we capture the transthioesterification intermediate and determine cryo‑EM structures of singly and doubly loaded UBA6-BIRC6^UBC^ complexes. Structural analysis reveals that UBA6, unlike UBA1, undergoes a ~ 30° rotation and a 19 Å displacement of its gate helix to create a complementary cavity that accommodates the BIRC6 insertion loop. Biochemical validation confirms that truncating the insertion loop rescues UBA1‑mediated charging, while swapping the UBA6 gate helix into UBA1 confers transthioesterification activity. Together, our findings provide structural and mechanistic insights into how UBA6 specifically engages BIRC6 rather than UBA1, advancing understanding of E1–E2 pairing specificity.

## Results

### The UBC domain of BIRC6 confers specificity for UBA6

**Fig. 1:**
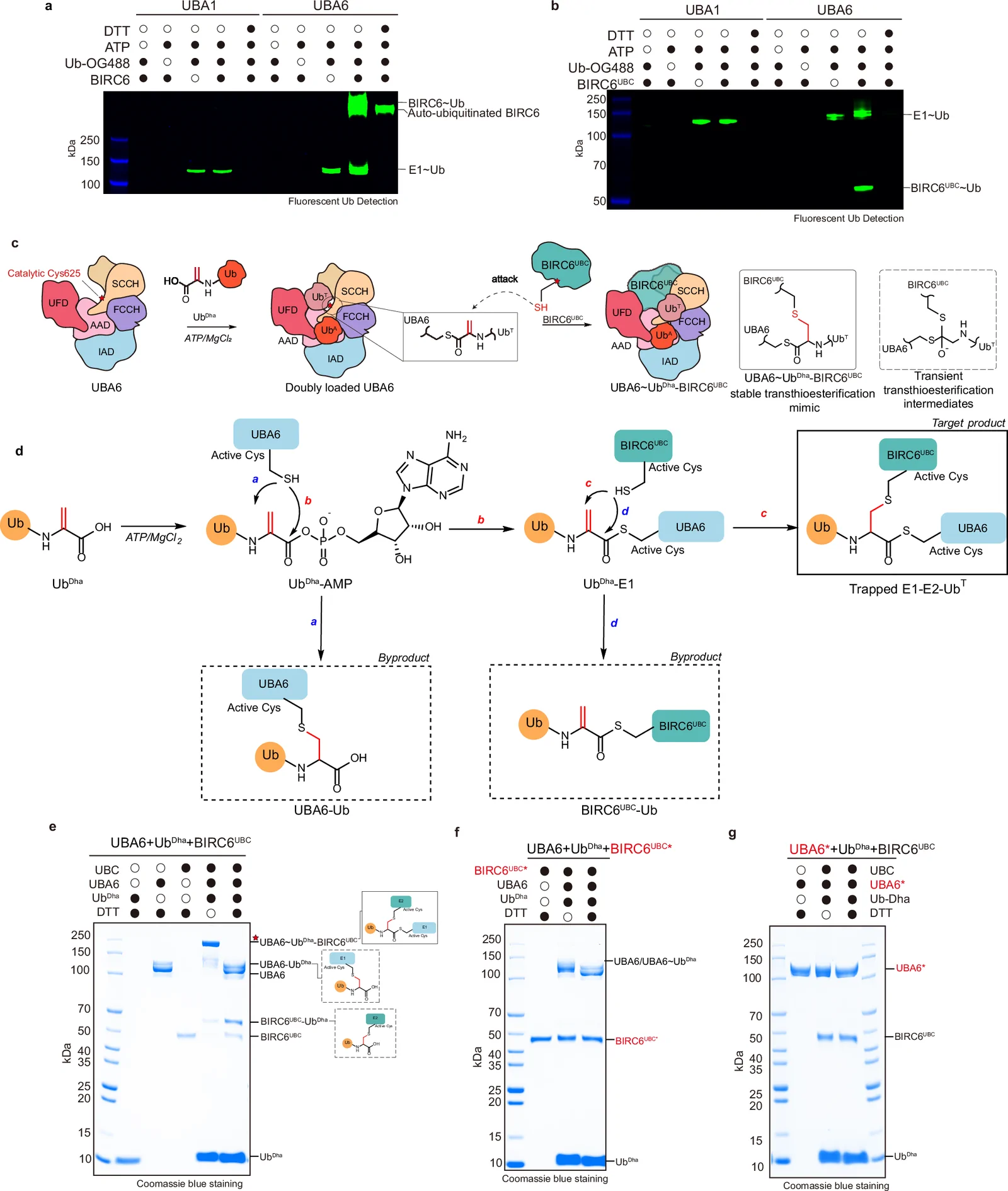
Chemical trapping of the UBA6-BIRC6 transthioesterification reaction. **a, b** E1–E2 transthioesterification assays for full-length BIRC6 (**a**) and the UBC domain of BIRC6, BIRC6^UBC^ (**b**). The Ub’s transfer was tracked by fluorescently labeled Ub (Ub-OG488). **c** Schematic illustrating chemical trapping of the UBA6-BIRC6^UBC^-Ub^T^-Ub^A^ complex. Ub^Dha^, a ubiquitin variant with residue G76 replaced by dehydroalanine, can be activated by UBA6 in a manner similar to natural ubiquitin to form doubly loaded UBA6. The electrophilic dehydroalanine then reacts with the active cysteine of the BIRC6^UBC^, generating a trapped transthioesterification mimic. The native and transient transthioesterification intermediates are also shown. **d** Reaction pathways of Ub^Dha^ in the transthioesterification reaction. Both the target product formation pathway and the byproduct formation pathway are shown. **e-g** SDS-PAGE analysis of the chemical trapping reaction for wild-type UBA6/BIRC6^UBC^ (**e**), BIRC6^UBC^ with the active-site C4666A mutation (**f**) and UBA6 with the active-site C625A mutation (**g**). BIRC6^UBC*^: the active‑site Cys4666 of BIRC6^UBC^ is replaced by alanine (C4666A). UBA6*: the active‑site Cys625 of UBA6 is replaced by alanine (C625A). Reactions were performed with 1 μM UBA6, 1 μM BIRC6^UBC^, and 12.5 μM Ub^Dha^ at 37 °C for 30 min. The formation of the target product depends on the catalytic cysteines of UBA6 and BIRC6^UBC^. The gel image (Fig. 1a, b, e, f, g) represents independent biological replicates (*n*  =  3).

**Fig. 2:**
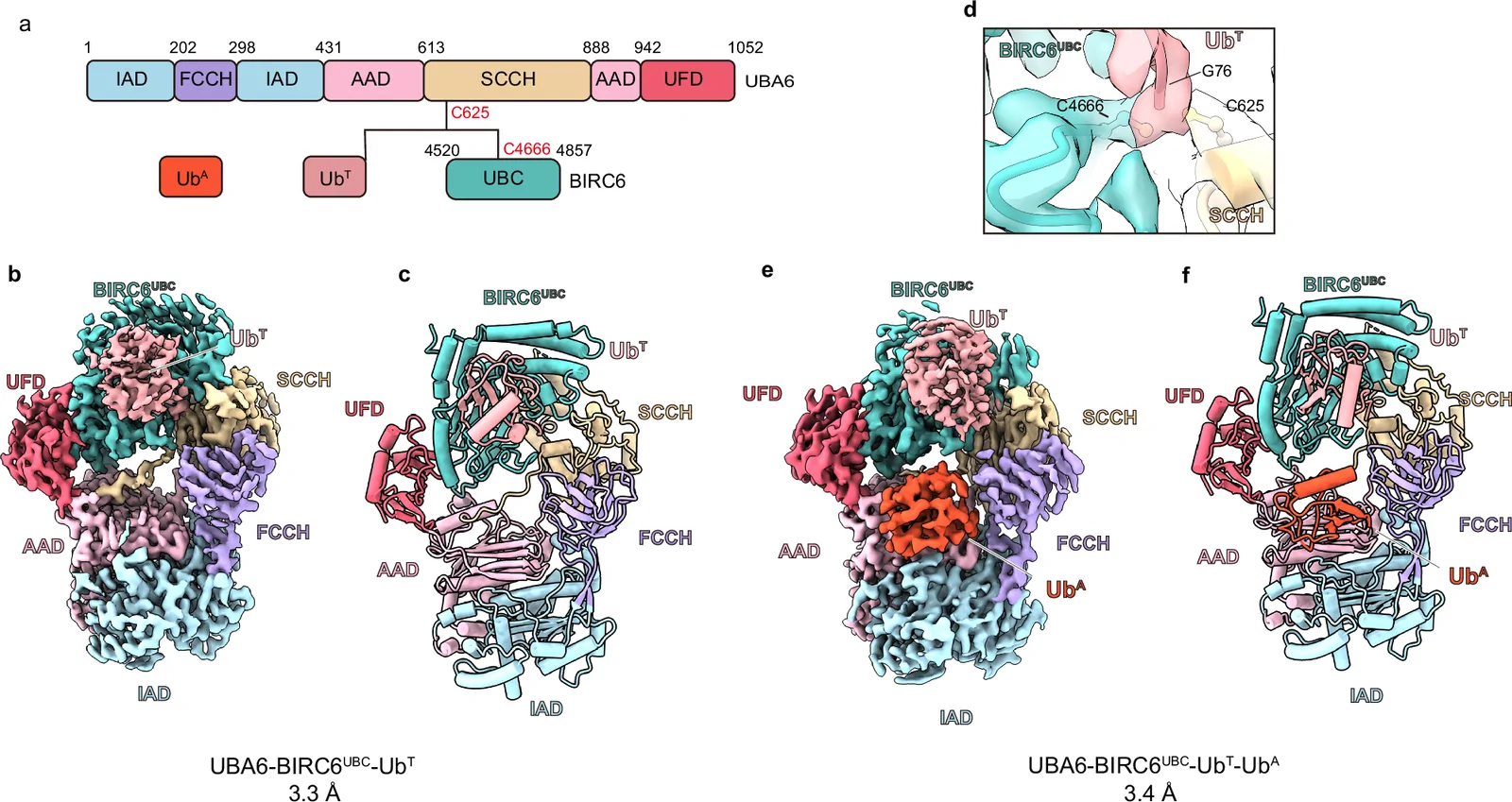
Cryo-EM structures of UBA6-BIRC6 transthioesterification mimic complexes. **a** Domain diagrams of UBA6, BIRC6^UBC^, Ub^A^, Ub^T^. The domains are colored and labeled accordingly. The black solid line indicates covalent linkages among UBA6, BIRC6^UBC^ and Ub^T^. **b, c** Cryo-EM map (**b**) and structural model (**c**) of singly loaded UBA6-BIRC6^UBC^-Ub^T^ complex. Each domain is labeled and the color scheme is the same as in (**a**). **d** A close-up view shows the local density and the fitted model at the transthioesterification center. **e, f** Cryo-EM map (**e**) and structural model (**f**) of doubly loaded UBA6-BIRC6^UBC^-Ub^T^-Ub^A^ complex.

To characterize the enzyme activity of BIRC6, we expressed full-length BIRC6 in HEK293F cells and reconstituted its activity through in vitro transthioesterification reactions. Our results showed that BIRC6 is specifically charged by UBA6, but not by UBA1 (Fig. 1a). This result is consistent with previous in vitro reconstitution data and the documented functional synergy between UBA6 and BIRC6 in autophagy regulation^26–28,32^. We further confirmed that the catalytic UBC domain of BIRC6 (residues 4520-4857) exhibited the same UBA6 preference as full-length BIRC6 (Figs. 1b, 2a), indicating that the selectivity of BIRC6 for UBA6 originates from its UBC domain. Based on this finding, we used the UBC domain of BIRC6 for subsequent mechanistic studies.

### One-step trapping of the E1–E2-Ub^T^-Ub^A^ complex

During the E1–E2 transthioesterification process, the E1 first catalyzes the adenylation of a ubiquitin molecule (Ubᴬ). The activated ubiquitin is then transferred to the catalytic cysteine of the E1, forming a thioester-linked ubiquitin (Ubᵀ). Subsequently, E1 recruits and adenylates a second ubiquitin molecule, resulting in a doubly loaded E1 complex with a 1:2 E1-to-ubiquitin stoichiometry^33,34^. This doubly loaded E1–E2-Ub^T^-Ub^A^ complex exhibits maximal transfer efficiency of Ub^T^ to the catalytic cysteine of the E2, establishing this state as the definitive active conformation for E1–E2 ubiquitination^34^. To better understand the molecular mechanisms underlying E1–E2 pairing specificity, we sought to determine the structure of the doubly loaded E1–E2-Ub^T^-Ub^A^ complex. However, capturing the structure of the doubly loaded E1–E2-Ub^T^-Ub^A^ complex is challenging due to the inherent instability of the thioester bond, the low affinity between E1 and E2, and the transient nature of the transthioesterification intermediates.

Activity-based ubiquitin probes have significantly advanced structural studies of ubiquitin-related enzymes^35–37^, including those involved in the E1–E2–E3 cascade^14,15,19–21,38–47^ and deubiquitinating enzymes (DUBs)^48–50^. Here, we employed the activity-based probe Ub^Dha^ to chemically trap the doubly loaded E1–E2-Ubᵀ-Ubᴬ complex^51^. Ub^Dha^ is commercially available or can be prepared in‑house through chemical synthesis by recombinantly expressing UbG76C, followed by converting the cysteine residue to the Dha moiety using 2,5-dibromohexanediamide in a one-step reaction (Supplementary Fig. 1a−c)^51^. Although residue 76 of Ub^Dha^ is replaced by the electrophilic dehydroalanine, Ub^Dha^ remains recognizable and can be processed by the natural ubiquitin machinery^51^. Ub^Dha^ can be activated at its C-terminus by E1 in a manner similar to natural ubiquitin, leading to the formation of a thioester-linked product E1~Ub^Dha^ intermediate, which can subsequently be transferred to E2s (Fig. 1c, d). Moreover, the electrophilic dehydroalanine of Ub^Dha^ can irreversibly react with the active cysteine residues of E1, E2, HECT/RBR-type E3 ligases and deubiquitinases^48–50^.

We next tested the ability of the Ub^Dha^ probe to covalently trap the UBA6-BIRC6^UBC^ complex. To this end, we mixed the Ub^Dha^ probe, UBA6, BIRC6^UBC^, and ATP/Mg²⁺ and optimized their concentrations and molar ratios. At the optimized molar ratio of UBA6:BIRC6^UBC^:Ub^Dha^ = 1:1:12.5, with UBA6 at a final concentration of 1 μM, nearly all UBA6 was cross-linked with BIRC6^UBC^ and Ub^Dha^, forming the doubly loaded UBA6-BIRC6^UBC^-Ub^T^-Ub^A^ complex (171 kDa) (Fig. 1e, lane 4; Supplementary Fig. 1d). The cross-linked byproducts UBA6-Ub^Dha^ (126 kDa) and BIRC6^UBC^~Ub^Dha^ (43 kDa) can be removed through size-exclusion chromatography, allowing isolation of high-purity doubly loaded UBA6-BIRC6^UBC^-Ub^T^-Ub^A^ complexes (Supplementary Fig. 1e, f). Additionally, mutating the active site C4666 of BIRC6^UBC^ or C625 of UBA6 to alanine resulted in negligible cross-linking bands, confirming that the reaction is activity‑dependent and specific (Fig. 1f, g). Importantly, this Ub^Dha^‑based method enables activity‑specific, site‑directed cross‑linking without requiring any point mutations or chemical modifications of the E1 or E2 enzymes.

### Structure of the UBA6-BIRC6^UBC^-Ub transthioesterification mimic

The vitrified samples were analyzed by cryo-EM, yielding two reconstructions: the singly loaded UBA6-BIRC6^UBC^-Ubᵀ complex at 3.3 Å resolution and the doubly loaded UBA6-BIRC6^UBC^-Ubᵀ-Ubᴬ complex at 3.4 Å resolution (Fig. 2b, e, Supplementary Figs. 2, 3a, 3b). Both constructs exhibited the identical overall architecture, with BIRC6^UBC^ sandwiched between the UFD and SCCH domains of UBA6. The difference between these reconstructions was the presence of additional density for the Ub^A^. This observation can be explained by the differential stability of the two ubiquitin moieties: Ubᵀ is covalently linked to UBA6 Cys625 via a thioester bond, whereas Ubᴬ is bound non‑covalently and is therefore more dynamic. The inherent lability of the non‑covalent connection makes Ubᴬ prone to dissociation during sample processing steps such as size‑exclusion chromatography and cryo‑EM vitrification. Importantly, the cryo-EM map unambiguously reveals clear density connecting the catalytic residues UBA6 Cys625, BIRC6^UBC^ Cys4666, and Ub^T^ Gly76, validating our trapping strategy (Fig. 2d).

Both reconstructions exhibit well-ordered UBA6 and BIRC6^UBC^, with local resolutions ranging from 2.9 to 3.4 Å. By contrast, Ub^T^ is comparatively disordered, with a local resolution ranging from 4.0 to 5.8 Å (Supplementary Fig. 3c). We therefore performed focused 3D classification, an approach first applied to capture ubiquitin transfer states by Kochańczyk and co‑workers in the context of *S. pombe* (Sp) UBA1-Ubc4 using the PSAN probe^13^. For both the singly loaded UBA6-BIRC6^UBC^-Ub^T^ and doubly loaded UBA6-BIRC6^UBC^-Ub^T^-Ubᴬ complexes, focused 3D classification yielded 10 reconstructions each, revealing pronounced Ub^T^ heterogeneity (Supplementary Fig. 3d, e). These reconstructions capture a conformational continuum—from Ub^T^ positioned near the FCCH domain of UBA6 to states proximal to BIRC6^UBC^. This spatial progression implies the process of the Ub^T^ transfer from UBA6 catalytic cysteine to BIRC6^UBC^ (Supplementary Fig. 3d, e). Inspection of the ATP catalytic pocket in both singly loaded UBA6-BIRC6^UBC^-Ub^T^ and doubly loaded UBA6-BIRC6^UBC^-Ub^T^-Ubᴬ structures revealed additional density that is compatible with AMP (Supplementary Fig. 3fi). Furthermore, in the doubly loaded state, an extra density putatively ascribed to the C‑terminal tail of Ubᴬ was observed (Supplementary Fig. 3h, i). These features indicate that the two captured complexes closely resemble Cluster 5 of the previously reported doubly loaded E1–E2 states (PDB: 9B5I), in which ATP is fully hydrolyzed to AMP, PPi•Mg²⁺ is absent, Ubᴬ is adenylated, and Ub^T^ occupies the donor position. This configuration is consistent with a mechanism in which Ubᴬ adenylation and PPi•Mg²⁺ release occur concomitantly and are coupled with transthiolation^13^.

Structural models were obtained for the singly loaded UBA6-BIRC6^UBC^-Ub^T^ and doubly loaded UBA6-BIRC6^UBC^-Ub^T^-Ubᴬ complexes via structure docking, manual refinement, and Phenix real-space refinement (Fig. 2c, f, Supplementary Figs. 3c, 4, 5). Because the doubly loaded UBA6-BIRC6^UBC^-Ub^T^-Ubᴬ complex exhibits maximal transfer efficiency of Ub^T^ to E2’s catalytic cysteine, subsequent analysis focuses on this doubly loaded complex.

### Combinatorial recognition of BIRC6^UBC^ by UBA6

**Fig. 3:**
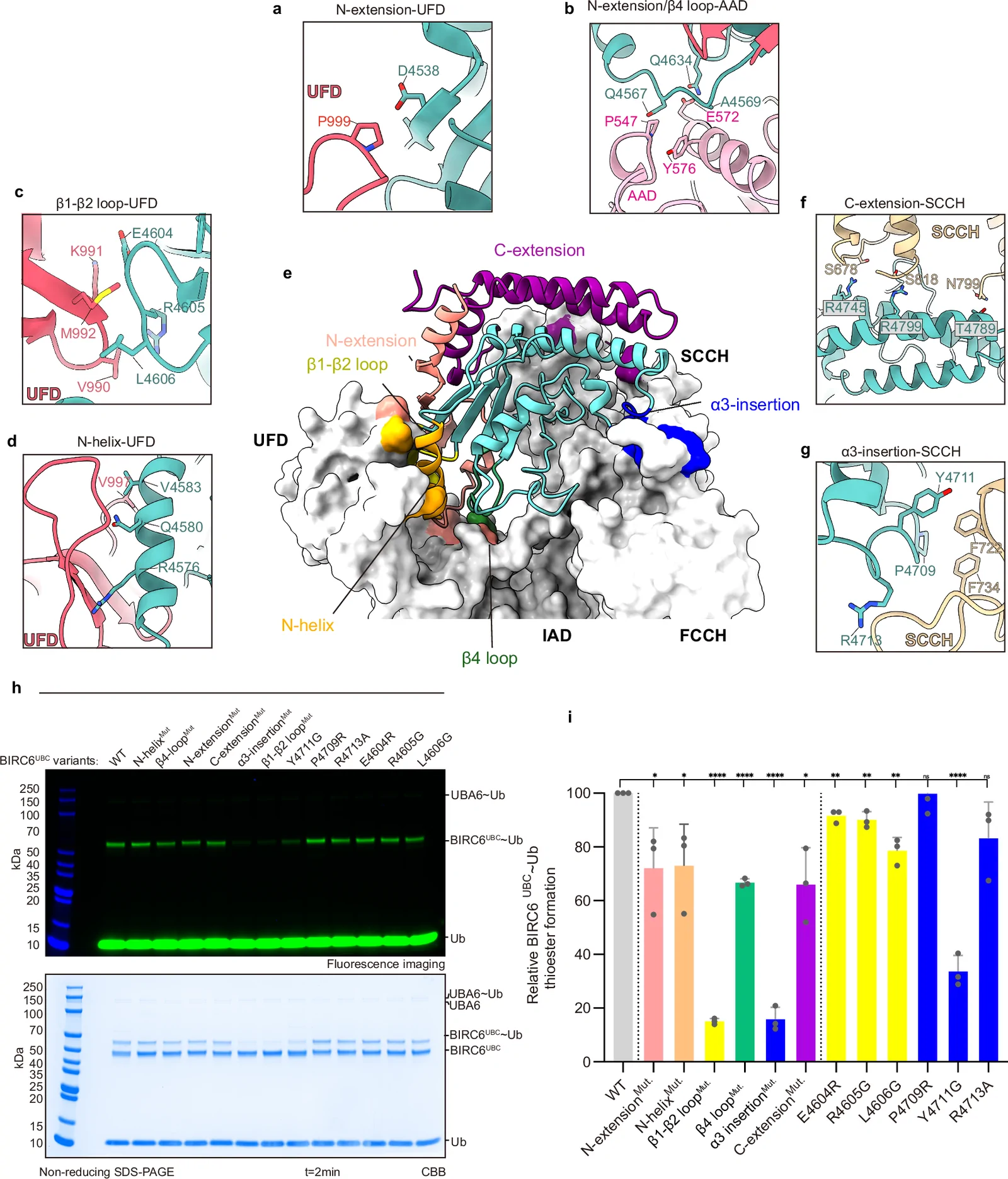
Molecular recognition of BIRC6^UBC^ by UBA6. **a** Close-up view of the interface between N-extension of BIRC6^UBC^ and the UBA6 UFD domain. The interacting residues are labeled. **b** Close-up view of the interface between N-extension/β4 loop of BIRC6^UBC^ and the UBA6 AAD domain. **c** Close-up view of the interface between the β1-β2 loop of BIRC6^UBC^ and the UBA6 UFD domain. **d** Close-up view of the interface between the N-helix of BIRC6^UBC^ and the UBA6 UFD domain. **e** Overall view of the contacts between BIRC6^UBC^ and UBA6. UBA6 is shown in surface representation, and BIRC6^UBC^ in cartoon representation. The different regions of BIRC6^UBC^ are colored and labeled, and the corresponding interacting regions on UBA6 are colored identically. **f** Close-up view of the interface between the C-terminal extension of BIRC6^UBC^ and the UBA6 SCCH domain. **g** Close-up view of the interface between α3-insertion of BIRC6^UBC^ and the UBA6 SCCH domain. **h** In vitro transthioesterification assays using fluorescently labeled Ub. Various BIRC6^UBC^ mutants were analyzed. The gel image represents independent biological replicates (*n*  =  3). Both fluorescent signals and the corresponding Coomassie Brilliant Blue-stained gels are shown. **i** Bar graph showing the fraction of BIRC6^UBC^~Ub presented as mean ± s.d. from *n*  =  3 independent experiments. Each data point is shown as a black dot (data from **h**). A two-sample, two-tailed Student’s unpaired t-test was used to calculate p values; ****p  <  0.0001, ***p  <  0.001, *p  <  0.05, exact p value: N-extension^Mut.^=0.0325, N-helix^Mut.^=0.0391, β1-β2^Mut.^< 0.0001, β4^Mut.^< 0.0001, α3-insertion^Mut.^< 0.0001, C-terminal extension^Mut^ = 0.0128, E4604R = 0.0037, R4605G = 0.005, L4606G = 0.0017, P4709R = 0.9648, Y4711G <  0.0001, R4713A = 0.0984. Reaction time: 2 min. Quantification was based on the Coomassie Brilliant Blue-stained gels and calculated as the percentage of BIRC6^UBC^~Ub relative to total BIRC6^UBC^, followed by normalization to WT BIRC6^UBC^.

The 40 human E2 enzymes share a conserved catalytic domain of approximately 150 amino acid residues, known as the UBC domain. The UBC domain comprises four α-helices and a four-stranded β-sheet connected by β-loops (Supplementary Fig. 6a). Beyond the canonical UBC scaffold, the UBC domain of BIRC6 features N-terminal/C-terminal extensions and internal insertions (e.g., the α3 insertion and insertion loop) that constitute structural elements critical for UBA6-BIRC6^UBC^ recognition (Supplementary Figs. 6b–d). Therefore, in contrast to other E1–E2 interactions, the molecular recognition between BIRC6^UBC^ and UBA6 is combinatorial: BIRC6^UBC^ engages with the UFD, AAD, and SCCH domains of UBA6 through six distinct interfaces. This interaction buries approximately 1400 Å² of the BIRC6^UBC^ surface area within the complex (Fig. 3e).

The first interface involves the N-terminal extension of BIRC6^UBC^ engaging the UFD and AAD domains of UBA6. The N-terminal extension of BIRC6^UBC^, positioned directly above the UBC domain, traverses the cleft between UFD and AAD and folds back to the N-helix of BIRC6^UBC^, forming multiple hydrophobic interactions involving both side chains and the backbone of UBA6 (Fig. 3e). Specifically, UFD residue P999 is spatially proximate to the backbone of BIRC6^UBC^ residue D4538 (Fig. 3a, Supplementary Fig. 7a), the backbone of BIRC6^UBC^ residue A4569 is near that of AAD residue Y576, and the AAD residue P547 is adjacent to the of backbone of BIRC6^UBC^ residue Q4567. These spatial relationships suggest potential hydrophobic interactions (Fig. 3b, Supplementary Fig. 7b). The second and third interfaces involve interactions between the BIRC6^UBC^ N-helix/β1-β2 loop and UFD (Fig. 3c, d, Supplementary Fig. 7c, d). Regarding the BIRC6^UBC^ N-helix/UFD interface, BIRC6^UBC^ residues R4576 and Q4580 are within hydrogen-bonding distance of UFD’s backbone carbonyl group. Furthermore, BIRC6^UBC^ residue V4583 may potentially participate in a hydrophobic interaction with UFD residue V997 (Fig. 3d, Supplementary Fig. 7d). In the β1-β2 loop/UFD interface, a potential van der Waals network connects the backbone frameworks of β1-β2 loop residues E4604-R4605-L4606 and UFD residues V990-K991-M992 (Fig. 3c, Supplementary Fig. 7c). Fourth, the residue Q4634 in the β4 loop of BIRC6^UBC^ may form a salt bridge with residue E572 in the AAD domain (Fig. 3b, Supplementary Fig. 7b). Fifth, the α3 insertion residues P4709 and Y4711 of BIRC6^UBC^ may interact with residues F722 and F734 in the SCCH domain through van der Waals forces, while residue R4713 of BIRC6^UBC^ is within hydrogen-bonding distance of SCCH domain backbone atoms (Fig. 3g, Supplementary Fig. 7e). The sixth interaction interface involves the C-terminal extension of BIRC6^UBC^ and the SCCH domain. The residues R4745, R4799, and T4789 in the C-terminal extension of BIRC6^UBC^ are within hydrogen-bonding distance of the backbone of residues S678 and S818, as well as residue N799 in the SCCH domain (Fig. 3f, Supplementary Fig. 7f).

To validate these interactions, we conducted E1–E2 transthioesterification assays using BIRC6^UBC^ with mutations at six different interfaces (Fig. 3h, i). The mutants included: an N-terminal extension mutant (A4569R); an N-helix mutant (R4576A-Q4580A-V4583G); a β1-β2 loop mutant (E4604R-R4605G-L4606G); a β4 loop mutant (Q4634A); an α3-insertion mutant (P4709R-Y4711G-R4745A); and a C-terminal extension mutant (R4745A-R4799A-T4789A). Both size-exclusion chromatography (SEC) and circular dichroism (CD) spectroscopy confirmed that all BIRC6^UBC^ mutants retained native folding, as they exhibited elution volumes and CD spectra comparable to wild‑type (WT) BIRC6^UBC^ (Supplementary Fig. 7g, h). The E1–E2 charging assays showed that mutations in the N-terminal extension and N-helix of BIRC6^UBC^ reduced Ub charging by by approximately 25% relative to WT. In contrast, mutations in the β4 loop and C-terminal extension resulted in a 50% reduction in activity, while mutations in the β1-β2 loop and α3 insertion almost abolished activity. To more precisely map the critical determinants, we subsequently performed single-residue substitutions at key positions within the β1-β2 loop (E4604R, R4605G, L4606G) and the α3 insertion (Y4711G, P4709R, R4713A) (Fig. 3h, i). The Y4711G mutation led to a 60% reduction in BIRC6^UBC^~Ub formation, whereas P4709R and R4713A showed minimal effects. Mutations within the β1-β2 loop (E4604R, R4605G, L4606G) each decreased Ub charging activity by 10-20%. These experiments revealed that Y4711 within the α3 insertion is the primary contributor to hydrophobic packing, whereas residues E4604, R4605, and L4606 in the β1-β2 loop collectively contribute to the interaction.

### Gating mechanism for E1–E2 pair specificity in transthioesterification

**Fig. 4:**
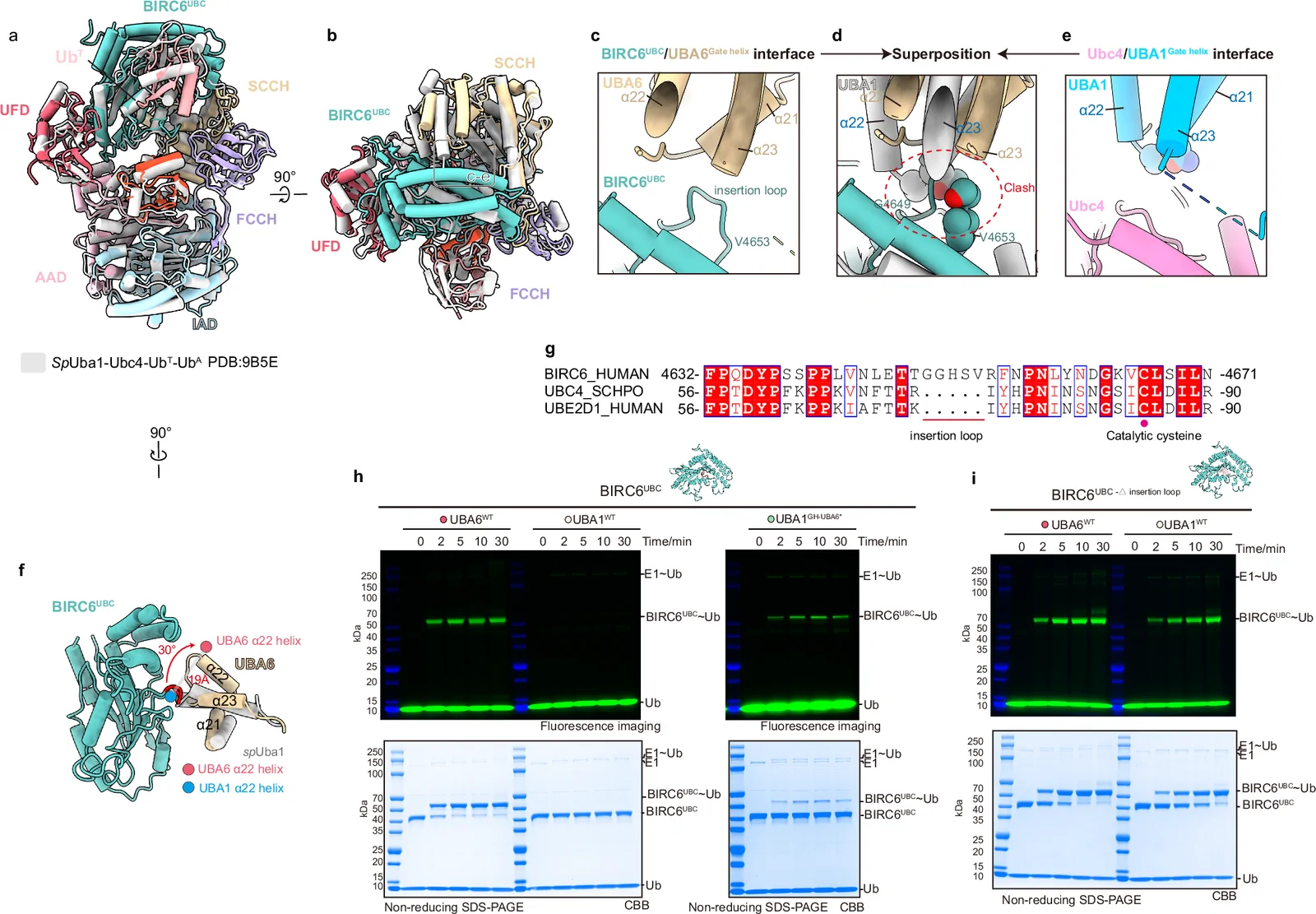
Structural identification and biochemical validation of the gate helix and insertion loop that determines UBA6-BIRC6^UBC^ pairing specificity. **a, b** Comparison of the doubly loaded UBA6-BIRC6^UBC^-Ub^T^-Ub^A^ complex with representative doubly loaded SpUBA1-Ubc4-Ub^T^-Ub^A^ complex structures (9B5E) revealed a pronounced steric clash between BIRC6^UBC^ and spUBA1. The rounded rectangle region corresponds to the close-up view shown in (**c–e**). Sp: *S. pombe*. **c** Close-up view of the spatial arrangement between BIRC6^UBC^ and the UBA6 gate helix (α22-α23 helix). The BIRC6^UBC^ (4649-4653) is shown and labeled as the insertion loop. **d** Structural alignment revealed a steric clash between the SpUBA1 gate helix (α22-α23 helix, amino acids 632-656) and the insertion loop of BIRC6^UBC^. **e** Close-up view of the spatial arrangement between Ubc4 and the SpUBA1 gate helix (α22-α23 helix, amino acids 632-656). **f** Relative to the α22 helix (amino acids 632-641) in UBA1, the α22 helix (amino acids 666-674) in UBA6 is rotated 30° clockwise and shifted by 19 Å, generating a space that accommodates the insertion loop of BIRC6^UBC^. **g** Sequence alignment shows BIRC6 contains a five-amino-acid insertion loop compared with other representative E2 enzymes (SpUbc4 and HsUbE2D1). **h** E1–E2 transthioesterification assays using UBA6^WT^, UBA1^WT^ or UBA1^GH-UBA6*^ as the E1 enzyme and BIRC6^UBC^ as the E2 enzyme. Time points: 0, 2, 5, 10, and 30 min. **i** E1–E2 transthioesterification assays using UBA6^WT^ or UBA1^WT^ as the E1 enzyme and BIRC6^UBCΔinsertion loop^ as the E2 enzyme. Time points: 0, 2, 5, 10, and 30 min. In these biochemical assays (**h** and **i**), fluorescently labeled ubiquitin was used, and both fluorescent signals and the corresponding Coomassie Brilliant Blue-stained gels are shown. The gel image represents independent biological replicates (n  =  2).

To elucidate the molecular basis of selective recognition of BIRC6^UBC^ by UBA6, we compared our resolved structures of doubly loaded UBA6-BIRC6^UBC^-Ub^T^-Ubᴬ complexes with the previously determined UBA1-E2-Ub structures (PDB: 9B5E, 9B5N, 4II2, 5KNL, 7K5J) (Supplementary Fig. 8a)^13,14,16,17^. Structural alignment revealed highly similar overall conformations, with root mean square deviation (RMSD) values ranging from 1.3 to 1.7 Å (Supplementary Fig. 8a). Further comparison of the doubly loaded UBA6-BIRC6^UBC^-Ubᵀ-Ubᴬ structure with the recently determined *S. pombe* UBA1-Ubc4-Ubᵀ-Ubᴬ complex (PDB: 9B5E) identified a critical structural distinction: although BIRC6^UBC^ and Ubc4 occupy equivalent positions on their respective E1 enzymes, a pronounced steric clash occurs between BIRC6^UBC^ and SpUBA1 (Fig. 4a, b)^13^. This clash was consistently observed in other aligned UBA1-E2-Ub structures, involving an insertion loop (residues 4649-4653) of BIRC6^UBC^ and the α22-α23 helix of SpUBA1 (referred to as the gate helix, amino acids 632-656) (Fig. 4c–e). Sequence alignment indicates that BIRC6^UBC^ has a five-amino-acid insertion loop compared to SpUbc4 (and its human homolog UbcH5a), which creates a spatial conflict with the gate helix of SpUBA1 (Fig. 4d, g). In contrast, the gate helix of UBA6 (residues 666-690) avoids this steric clash through a 30° clockwise rotation and 19 Å movement of its α22-helix, thereby accommodating the insertion loop of BIRC6^UBC^ (Fig. 4f).

To validate whether steric clashes prevent UBA1-activated ubiquitin transfer to BIRC6^UBC^, we truncated the insertion loop of BIRC6^UBC^, then measured its transthioesterification activity towards UBA1 and UBA6. Deletion of the insertion loop of BIRC6^UBC^ (referred to as BIRC6^UBCΔinsertion loop^) exhibited transthioesterification activity toward UBA6 comparable to that of WT BIRC6^UBC^, indicating that truncation of the insertion loop does not affect the intrinsic activity of BIRC6^UBC^ (Fig. 4h, i, Supplementary Fig. 8j). In contrast, BIRC6^UBCΔinsertion loop^ rescued UBA1‑mediated ubiquitin charging (Fig. 4i, Supplementary Fig. 8j).

We next engineered chimeric E1 enzymes by exchanging the gate helix. Specifically, we generated a chimera in which UBA6’s gate helix was replaced with that of UBA1 (referred to as UBA6^GH-UBA1*^), and a chimera in which UBA1’s gate helix was replaced with that of UBA6 (referred to as UBA1^GH-UBA6*^). E1~Ub charging assays showed that UBA6^GH-UBA1*^ exhibited little to no ability to activate ubiquitin, whereas UBA1^GH-UBA6*^ retained approximately 50% of the activity compared to UBA1^WT^ (Supplementary Fig. 8f). Consistent with this, transthioesterification assays using the promiscuous E2 UbcH5c revealed that UBA6^GH-UBA1*^ failed to catalyze UbcH5c~Ub formation, while UBA1^GH-UBA6*^ remained capable of charging UbcH5c, albeit with reduced efficiency (Supplementary Fig. 8g, h). Because the UBA6^GH-UBA1*^ chimera is defective in the ubiquitin transfer cascade, precluding further transthioesterification assays with the BIRC6^UBC^. Therefore, we tested only UBA1^GH-UBA6*^ for its transthioesterification activity toward BIRC6^UBC^. A time‑course quantitative comparison of the activities of UBA1^WT^, UBA6^WT^, and UBA1^GH-UBA6*^ toward BIRC6^UBC^ showed that the UBA1^GH-UBA6*^ chimera partially restored this activity, whereas UBA1^WT^ showed no detectable charging of BIRC6^UBC^ (Fig. 4h, Supplementary Fig. 8i).

In conclusion, although the loss of UBA6^GH-UBA1*^ E1 activity precluded reciprocal validation, both UBA1^GH-UBA6*^and BIRC6^UBCΔinsertion loop^ partially restored transthioesterification activity, supporting a model in which the gate helix and insertion loop are critical determinants of UBA6-BIRC6 ubiquitin transfer specificity. Mechanistically, a 30° rotation and and 19 Å movement in the UBA6 gate helix avoids steric clash with the BIRC6^UBC^ insertion loop, whereas the UBA1 gate helix imposes a spatial conflict, thereby establishing exclusive BIRC6^UBC^ selectivity for UBA6.

### BIRC6 preferentially binds UBA6/UBA6-Ub over UBA1/UBA1-Ub

After identifying the gate helix in the SCCH domain as a key determinant of UBA6-BIRC6 specificity, we next investigated whether differences in binding affinity between BIRC6 and UBA1 versus UBA6 also contribute to specificity. We prepared oxyester‑linked UBA1-Ub and UBA6-Ub conjugates (where “‑” denotes the oxyester linkage) to mimic the unstable thioester intermediates UBA1~Ub and UBA6~Ub. Subsequently, we compared the binding affinities of BIRC6^UBC^ for UBA6, UBA6‑Ub, UBA1, and UBA1‑Ub using bio‑layer interferometry (BLI). The results showed that BIRC6^UBC^ binds to UBA6 and UBA6‑Ub with steady‑state K_D_ values of 4.82 μM and 2.41 μM, respectively (Supplementary Fig. 9a, b, f). In contrast to UBA6 and UBA6‑Ub, which gave clear concentration‑dependent responses and allowed robust steady‑state fitting, the sensorgrams for UBA1 and UBA1‑Ub largely overlapped across the 40-1080 nM range, with only a modest signal increase observed at 3240 nM (Supplementary Fig. 9c, d, f). Consequently, the datasets lacked the necessary concentration‑dependent variation for reliable steady‑state affinity fitting. These BLI results reconcile our findings with the size‑exclusion chromatography (SEC) co‑elution profiles reported by Riechmann et al., confirming that BIRC6^UBC^ preferentially interacts with its cognate UBA6 rather than UBA1^52^.

Furthermore, BIRC6^UBCΔinsertion loop^ regained detectable binding to UBA1, with a steady-state K_D_ of 1.47 μM (Supplementary Fig. 9e, f). This gain in binding affinity is consistent with our in vitro transthioesterification assays, in which deletion of the insertion loop rescued UBA1-mediated ubiquitin charging of BIRC6^UBC^ (Fig. 4i). Together with the above binding data, these observations support our model that the BIRC6^UBC^ insertion loop and the UBA1 gate helix are key determinants in excluding UBA1 while enabling UBA6 specificity.

## Discussion

**Fig. 5:**
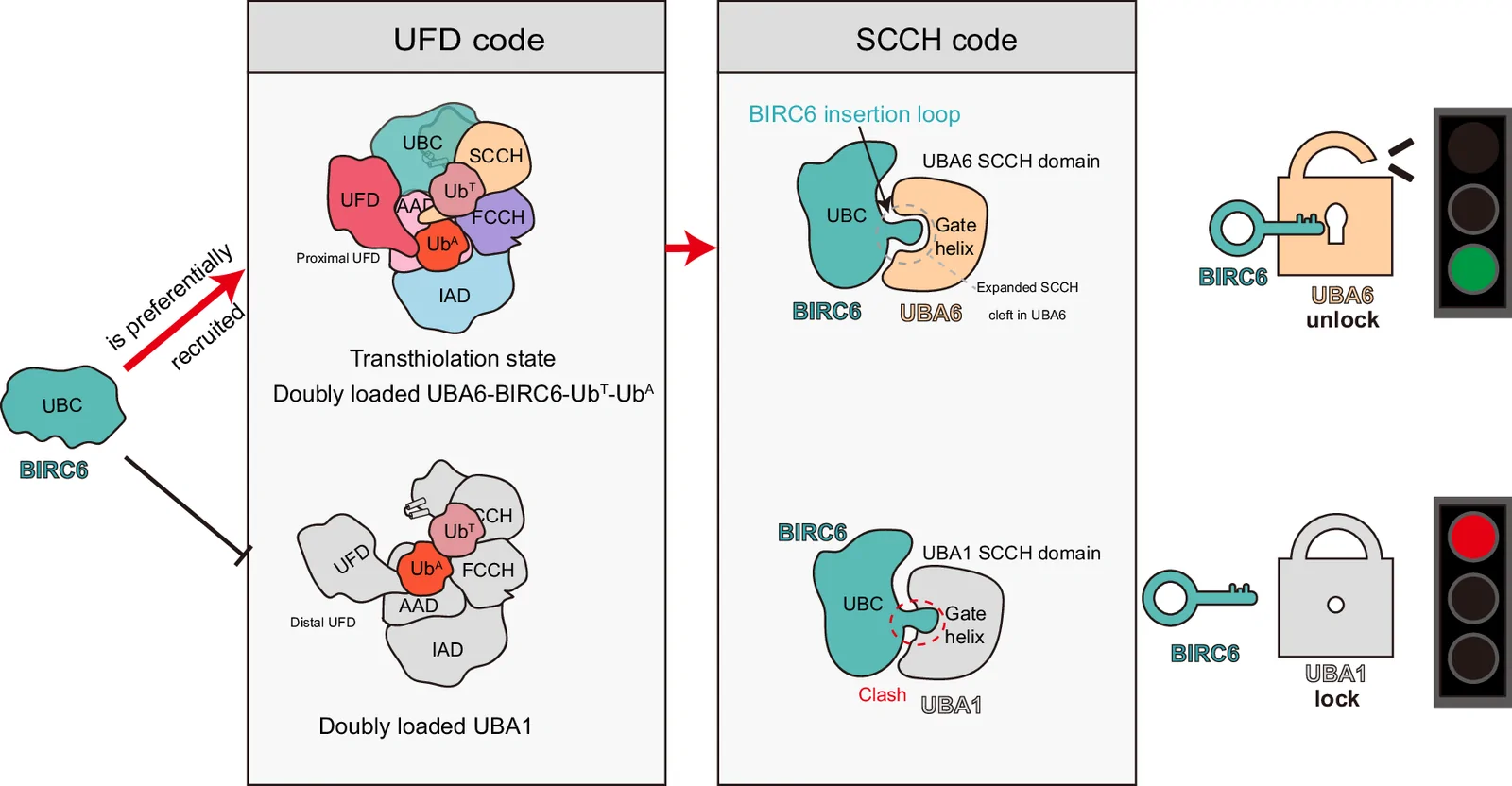
Dual code model for the preferential transthioesterification of BIRC6^UBC^ by UBA6 over UBA1. 1. UFD Code. BIRC6 is preferentially recruited to UBA6~Ub rather than UBA1~Ub. 2. SCCH gate helix code. The SCCH domain of UBA6 contains an expanded cleft, and the gate helix adopts a distinct conformation structurally configured to accommodate the insertion loop of BIRC6^UBC^. The insertion loop fits into a compatible cavity created by the rotated gate helix, enabling catalysis (unlock). In contrast, UBA1 fails to catalyze transthioesterification due to a steric clash between the insertion loop of BIRC6^UBC^ and the gate helix of UBA1, which prevents productive engagement (lock).

Although previous studies have systematically resolved the structural landscape of the UBA1-E2 transthioesterification cycle, the molecular mechanism by which UBA6, the second Ub-activating enzyme with non-redundant physiological functions in the ubiquitin system, selectively recognizes its specific E2 partners remains largely unknown. Here, we elucidated the molecular mechanism underlying the UBA6-specific activation of the giant E2–E3 chimera BIRC6. Using an activity-based Ub^Dha^ probe, we captured and visualized the cryo-EM structures of the transthioesterification intermediate complexes, doubly loaded UBA6-BIRC6^UBC^-Ubᵀ-Ubᴬ and singly loaded UBA6-BIRC6^UBC^-Ubᵀ. Based on structural comparison, sequence alignment, and biochemical validation, we identified an insertion loop in BIRC6^UBC^ and the gate helix (α22-α23 helix) in the SCCH domain of UBA6 as contributing factors to BIRC6-UBA6 specificity. In UBA6, the gate helix within the SCCH domain adopts a distinct conformation structurally configured to accommodate an insertion loop of BIRC6^UBC^. In contrast, the corresponding gate helix in UBA1 extends inward toward the E2, causing steric clashes with the insertion loop of BIRC6 (Fig. 5).

### Complementary insights into UFD‑ and SCCH‑mediated UBA6-BIRC6 specificity

During the review of our manuscript, two independent studies were published that also focused on the molecular mechanisms by which UBA1 and UBA6 achieve selective E2 engagement through fundamentally distinct domain architectures^52,53^. Using domain‑swap experiments, both studies demonstrated that the UFD and SCCH domains are essential for UBA6-BIRC6 specificity. Riechmann et al. further determined the structures of *human* UBA6-BIRC6^UBC^-Ub and *Drosophila* UBA1-Ub^Dha^-BIRC6^UBC^, elucidating the molecular mechanism by which the UFD domain of UBA6 drives selective recognition of BIRC6. The UFD domain of UBA6 presents a more hydrophobic E2‑binding surface than that of UBA1, and BIRC6^UBC^ contains a set of hydrophobic residues optimally oriented to complement this surface, thereby providing a basis for selective engagement. Moreover, they mapped the specificity determinants within the UFD to a key pair of residues (E4603 and E4604) in the β1-β2 loop of BIRC6. Consistent with this observation, disruption of the β1-β2 loop-UFD interface in BIRC6 led to a nearly complete loss of UBA6-mediated BIRC6^UBC^ charging activity in our study. On the other hand, our BLI binding data showed that BIRC6^UBC^ preferentially interacts with UBA6/UBA6-Ub rather than UBA1/UBA1-Ub. This observation is consistent with the conclusion by Riechmann et al. that a stable interaction occurs only with its cognate E1, UBA6, supporting the notion that initial recruitment via the UFD is another critical determinant of UBA6-BIRC6 specificity.

Inspired by the groundbreaking work of Riechmann et al. and based on our own findings, we propose a dual‑code model to explain the molecular basis of UBA6-BIRC6 selectivity (Fig. 5). The first code, mediated by the UFD domain and demonstrated by Riechmann et al., acts as the primary binding interface that governs initial partner selection. The second code, discovered in this study and mediated by the SCCH domain’s gate helix, functions as a catalytic gatekeeper. The unique conformation of the UBA6 gate helix creates a sterically compatible pocket that accommodates the insertion loop of BIRC6^UBC^. This structural alignment positions the BIRC6^UBC^ active site appropriately relative to the UBA6~Ub thioester intermediate, enabling efficient transthioesterification (the “unlock” state). In contrast, the UBA1 gate helix causes steric hindrance with the BIRC6^UBC^ insertion loop, which blocks the UBC domain from adopting the correct orientation required for catalysis (the “lock” state).

Our study complements these findings by revealing the role of the SCCH domain in this specificity. Together, these studies elucidate distinct yet synergistic layers of the UBA6-BIRC6 specificity mechanism through the UFD and SCCH domains, thereby providing a more complete mechanistic framework.

### A similar dual‑domain code underlies UBA6‑specific recognition of UBE2Z

In addition to BIRC6, UBE2Z represents another E2 enzyme specifically charged by UBA6^10,24,54^. Structural and sequence alignments reveal that UBE2Z (PDB: 5A4P) also contains a five‑residue insertion loop, which is likely to cause steric clash with the gate helix of UBA1 (Supplementary Fig. 10a–d). Therefore, our structural model may also explain the UBA6-specific activation mechanism of UBE2Z. In a recently published study, Nayak et al. elucidated the molecular mechanism underlying UBA6-UBE2Z specificity, showing that it is jointly determined by the UFD and SCCH domains^53^. First, the acidic β30-β31 loop of the UBA1 UFD domain interacts with the basic helix A of UBE2Z to ensure initial recruitment. Second, the SCCH domain supplies a pre‑organized catalytic scaffold that accommodates the insertion of the LA and LC loops of UBE2Z; the LA loop corresponds to the five‑residue insertion loop we identified in BIRC6.

Thus, together with the work by Riechmann et al. and Nayak et al., our studies complement and reinforce one another, providing a more complete mechanistic understanding of UBA6‑specific E2 recognition. In this model, UBA6 determines BIRC6 specificity through a dual‑domain code involving both the UFD and SCCH domains: the UFD provides an initial recruitment platform that governs partner selection, while the SCCH optimizes the catalytic geometry.

### Expanding the utility of Ub^Dha^ probes for structural studies of ubiquitin transfer

Ub^Dha^ is a commercially available or readily synthesized chemical probe that has been used in activity-based profiling to target E1–E2–E3 ubiquitin enzymes. It is suitable for both proteomic analysis and monitoring enzymatic activities in living cells^51^. In this study, we expanded the application of Ub^Dha^ to structural biology by demonstrating its ability to directly and conveniently capture structures of the transthioesterification intermediate within the doubly loaded E1–E2-Ubᵀ-Ubᴬ complex. Furthermore, focused 3D classification of Ubᵀ revealed a continuum of conformations depicting ubiquitin transfer from the FCCH domain to the E2 active site, underscoring the strong potential of Ub^Dha^ for resolving dynamic architectures along the ubiquitination cascade. The Ub^Dha^ probe shows strong potential for capturing transthioesterification intermediates of HECT and RBR E3 ligases in complex with E2 and ubiquitin, and holds promise for extension to other ubiquitin-like protein (UBL) systems, highlighting its broad value as a chemical tool for studying UB/UBL cascades.

## Methods

### Plasmids and molecular cloning

The pDARMO-3×FLAG-BIRC6 plasmid was purchased from Addgene (plasmid #197967, deposited by Eric Fischer)^27^. The cDNA of human UBA6 (Uniprot entry A0AVT1, full-length, amino acid residues 1-1052), BIRC6 UBC domain (Uniprot entry Q9NR09, amino acid residues 4520-4857) were synthesized with codon optimization for *Escherichia coli* recombinant expression by GenScript Biotech (Nanjing, China). The UBA6 sequence was subsequently cloned into a pDARMO_CMVT vector with an N-terminal 3×FLAG tag. The UBC domain of BIRC6 (BIRC6^UBC^, residues 4520-4857) and UbcH5c sequences were cloned into the pET-28a vector with an N-terminal His_6_ tag, followed by an HRV 3C protease cleavage site. Plasmids of human UBA1 and human ubiquitin were constructed in accordance with previous study^41^. The generation of protein mutants and truncations was achieved through standard site-directed PCR mutagenesis or homologous recombination techniques and verified by DNA sequencing (Tsingke, Beijing).

### Protein expression and purification

BIRC6, UBA6, and UBA6 mutants were expressed in mammalian cells. HEK293F cells (Thermo Fisher Scientific, Cat#A14528) were cultivated in Union-293 chemically defined medium (Union-Biotech, UP10000) and transfected using polyethyleneimine (PEI, Polysciences) at a density of 1.5-2.0×10^6^ cells·mL^-1^. After 60 h of transfection, the transfected cells were harvested at 5,000 g, resuspended in lysis buffer (50 mM HEPES, pH 7.4, 200 mM NaCl, 5% (v/v) glycerol) supplemented with EDTA-free cOmplete protease inhibitor tablets (Roche, 04693132001). Cells were lysed by sonication and centrifuged at 30,000 g for 45 min at 4 °C. The supernatant was incubated with anti-DYKDDDDK affinity beads (Smart-Lifesciences, SA042025) for 2 h at 4 °C. Bound proteins were eluted with lysis buffer containing 1 mg·mL⁻¹ FLAG peptide. The eluted proteins were then subjected to further purification using a 5 mL Hitrap Q HP column (Cytiva #17115301) (IEX buffer A: 50 mM HEPES, pH 7.4; IEX buffer B: 50 mM HEPES, pH 7.4, 1 M NaCl). Indicated peak fractions were concentrated and further purified using size-exclusion chromatography (SEC, Superose 6 Increase 10/300 GL or Superdex 200 Increase 10/300 GL, Cytiva) in SEC buffer (30 mM HEPES, pH 7.4, 150 mM NaCl).

UBA1, UBA1 mutants, BIRC6^UBC^, BIRC6^UBC^ mutants and UbcH5c were expressed in *Escherichia coli* BL21 (DE3) cells (Transgene). Cells were grown at 37 °C in Luria-Bertani (LB) medium to an OD_600_ of approximately 0.8 and induced overnight with 0.4 mM isopropyl β-D-thiogalactopyranoside (IPTG) at 16 °C. After harvesting, the cell pellets were resuspended in lysis buffer (50 mM HEPES, pH 7.4, 150 mM NaCl) supplemented with 1 mM phenylmethylsulfonyl fluoride (PMSF). Cells were lysed by sonication and centrifuged at 30,000 g for 30 min at 4 °C. The resulting supernatant was incubated with Ni-NTA affinity resin at 4 °C for 1 h (Smart-Lifesciences, SA004500). Bound proteins were subsequently eluted using lysis buffer supplemented with 300 mM imidazole. For BIRC6^UBC^, BIRC6^UBC^ mutants, and UbcH5c, HRV 3C protease was added to remove the His_6_ tag, and the protein was subsequently purified using a Superdex 75 Increase 10/300 GL size-exclusion column (Cytiva) in SEC buffer (50 mM HEPES, pH 7.4, 150 mM NaCl). For UBA1 and its mutants, the protein was purified by ion exchange chromatography using a 5 mL Hitrap Q HP column (IEX buffer A: 50 mM HEPES, pH 7.4; IEX buffer B: 50 mM HEPES, pH 7.4, 1 M NaCl). Indicated peak fractions were concentrated and further purified using SEC on a Superdex 200 Increase 10/300 GL column (Cytiva).

### Circular dichroism (CD) spectroscopy

BIRC6^UBC^ and its mutants were dialyzed into CD buffer (20 mM sodium dihydrogen phosphate, pH 7.5) overnight. Each sample was then diluted to 0.1 mg·mL^-1^. Circular dichroism (CD) spectra were recorded on a Chirascan Plus spectrometer (Applied Photophysics, Leatherhead, Surrey, United Kingdom) at 25 °C using a 0.1 cm cuvette. Spectra were collected from 200 to 280 nm, averaged over three scans, and corrected by subtracting the buffer contribution. Data were fitted using the Chirascan software, and plotting was performed in Origin 2024.

### Fluorescent labeling of ubiquitin

MCQ-Ub (1 equivalent, a ubiquitin variant with an additional cysteine inserted between the N-terminal methionine and glutamine) was concentrated to approximately 2 mM in reaction buffer (20 mM HEPES, pH 7.5, 150 mM NaCl, 0.5 mM Tris (2-carboxyethyl) phosphine (TCEP, Aladdin). Subsequently, 2 equivalents of Oregon GreenTM 488 (OG488) maleimide (dissolved in dimethyl sulfoxide (DMSO), purchased from Thermo Fisher Scientific, O6034) were added, and the pH was adjusted to 7.4. The above reaction was conducted at 37 °C in the dark for 1 h, and then quenched by the addition of 50 mM dithiothreitol (DTT). The reaction was monitored by analytical reversed-phase high-performance liquid chromatography (RP-HPLC). The final product, fluorescently labeled ubiquitin (Ub^OG488^), was purified using a Superdex 75 Increase 10/300 GL column (Cytiva) in SEC buffer (50 mM HEPES, pH 7.4, 150 mM NaCl).

### Preparation of Ub^Dha^

UbG76C (1 equivalent, a ubiquitin variant in which C-terminal Gly76 is mutated to cysteine) was dissolved in reaction buffer (6 M Gn-HCl, 100 mM NaH_2_PO_4_, pH 9.0), and then DTT was added to a final concentration of 0.5 mM. 50 equivalents of α,α’-di-bromo-adipyl(bis)amide (dissolved in DMSO) were added to the above reaction, and the pH was adjusted to 9.5. The reaction was conducted at 37 °C for 2.5 h. The final product was separated by semi-preparative HPLC (214 nm) and characterized by ESI-MS.

After lyophilization, Ub^Dha^ was dissolved in buffer A (8 M urea, 20 mM HEPES, pH 7.4, 150 mM NaCl). Subsequently, buffer B (20 mM HEPES, pH 7.4, 150 mM NaCl) was slowly added and thoroughly mixed until the urea concentration was diluted to 0.5 M. This Ub^Dha^ probe was further purified using a Superdex 75 Increase 10/300 GL column (Cytiva) pre-equilibrated with SEC buffer (50 mM HEPES, pH 7.4, 150 mM NaCl).

### Preparation of oxyester‑linked E1~Ub mimics

Purified UBA1 C623S (active Cys 623 mutated to Ser) (5 µM) and UBA6 C625S (active Cys 625 mutated to Ser) (5 µM) were separately incubated with Ub (50 µM) in reaction buffer (50 mM HEPES, pH 7.4, 150 mM NaCl, 5 mM MgCl_2_, and 10 mM ATP) at 37 °C overnight. Reactions were monitored through SDS-PAGE. Subsequently, oxyester‑linked UBA1-Ub and UBA6-Ub were purified by size-exclusion chromatography through a Superdex 200 Increase 10/300 GL column (Cytiva) equilibrated in SEC buffer (50 mM HEPES, pH 7.4, 150 mM NaCl).

### Reconstitution of UBA6-BIRC6^UBC^-Ub transthioesterification mimic

For the preparation of the UBA6-BIRC6^UBC^-Ub transthiolation mimic, 1 µM UBA6, 1 µM BIRC6^UBC,^ and 12.5 µM Ub^Dha^ were mixed in the reaction buffer (50 mM HEPES, pH 7.4, 150 mM NaCl, 5 mM MgCl_2_, and 10 mM ATP) at 37 °C for 1 h. The reaction was monitored through SDS-PAGE. The crosslinked product was further purified by size-exclusion chromatography through a Superdex 200 Increase 10/300GL column (Cytiva) equilibrated in SEC buffer (50 mM HEPES, pH 7.4, 150 mM NaCl).

### Cryo-EM sample preparation and data collection

3.5 µL of the prepared complex was added to the glow-discharged holey gold grids (Quantifoil R1.2/1.3, Au mesh), and incubated at 4 °C under 100% humidity for 60 seconds. Grids were rapidly   plunged into liquid ethane using the Vitrobot (Thermo Fisher Scientific, blot time 3 s, blot force 3).

A total of 21,555 cryo-EM micrographs of the UBA6-BIRC6^UBC^-Ub^Dha^ dataset were collected on a 300 kV Titan Krios G4 microscope configured with a Falcon4 direct electron detector camera using the EPU software (Thermo Fisher Scientific). Micrographs for this dataset were acquired at 96000× magnification, corresponding to a pixel size of 0.808 Å, under identical exposure conditions. Each image was recorded over 32 frames within 5.59 seconds, with defocus values ranging from -1.5 to -2.0 μm.

### Cryo-EM image processing

All datasets were processed using RELION v3.1.1^55^. The following procedures were performed: motion correction^56^, contrast transfer function (CTF) estimation^57^, manual or automated particle picking, and particle extraction. Subsequent steps in the methodology comprised iterative 2D and 3D classifications, mask generation, 3D auto-refinement, and postprocessing. The data processing flow charts are detailed in Supplementary Fig. 2. The statistics pertaining to the processing of cryo-EM data have been summarized in Table 1. The determination of overall resolutions was based on the gold standard Fourier shell correlation (FSC) criterion at 0.143, and local resolution estimations were carried out using ResMap v1.1.4.

**Table 1:** Cryo-EM data collection, refinement, and validation statistics

	Singly loaded UBA6-BIRC6^UBC^-Ub^T^ complex (EMD- 66842, PDB 9XGB)	Doubly loaded UBA6-BIRC6^UBC^-Ub^T^-Ub^A^ complex (EMD- 66843, PDB 9XGC)
Magnification	96000	96000
Voltage (kV)	300	300
Spherical aberration (mm)	2.7	2.7
Detector	Falcon4	Falcon4
Electron exposure (e^-^/Å2)	51.35 e^–^, 32 frames	51.35 e^–^, 32 frames
Defocus range (μm)	-1.5 to -2.0	-1.5 to -2.0
Pixel size (Å)	0.808	0.808
Symmetry imposed	C1	C1
Box size (pixel)	200	200
Micrographs (no.)	21555	21555
Initial particle images (no.)	14,438,711	14,438,711
Final particle images (no.)	1,201,224	1,087,360
Map resolution (Å)	3.3 Å	3.4 Å
Map resolution range	2.9-5.8 Å	2.9-5.8 Å
FSC threshold	0.143	0.143
Initial model use (PDB code)	1UBQ, 3CEG, 7PVN	1UBQ, 3CEG, 7PVN
Model resolution (Å)	4.0 Å	3.5 Å
FSC threshold	0.5	0.5
Map sharpening B factor (Å2)	-180	-180
Nonhydrogen atoms	10826	11421
Protein residues	1366	1440
Nucleotides	0	0
Protein	30.00/534.77/209.50	131.16/461.09/237.04
Nucleotide	---	---
Bond lengths (Å)	0.009	0.008
Bond angles (°)	1.428	1.143
MolProbity score	1.66	1.71
Clashscore	6.76	7.76
Poor rotamers (%)	1	0.08
Favored (%)	95.88	95.86
Allowed (%)	4.05	4.14
Disallowed (%)	0.07	0

### Model building and refinement

The cryo-EM density maps of the singly loaded UBA6-BIRC6^UBC^-Ubᵀ complex and the doubly loaded UBA6-BIRC6^UBC^-Ubᵀ-Ubᴬ complex were employed for structural modeling. In the case of the singly loaded UBA6-BIRC6^UBC^-Ubᵀ assembly, the atomic coordinates of BIRC6^UBC^ (PDB: 3CEG)^54^, ubiquitin (PDB: 1UBQ)^58^, and UBA6 (PDB: 7PVN) ^11^were fitted as rigid bodies into the cryo-EM density using ChimeraX-1.7.1^59^. The resulting coordinates, which preserved spatial positioning, were subsequently imported into WinCoot-0.8.2, where BIRC6^UBC^, Ubᵀ, and UBA6 were combined into a single initial model^60^.

For the doubly loaded UBA6-BIRC6^UBC^-Ubᵀ-Ubᴬ complex, the structure of the singly loaded UBA6-BIRC6^UBC^-Ubᵀ was manually positioned together with Ubᴬ in ChimeraX-1.7.1, followed by integration of Ubᴬ in WinCoot-0.8.2 to produce the starting model. Initial models were refined in Phenix-1.19.2 through real-space refinement, incorporating secondary structure constraints and geometric restraints^61^. All models underwent manual inspection and adjustment in WinCoot-0.8.2. A summary of the 3D reconstruction and modeling statistics is provided in Table 1. Structural visualizations were generated in ChimeraX-1.7.1, from which figures were exported for presentation.

### E1~Ub thioester formation assays

E1~Ub thioester formation assays (as shown in Supplementary Fig. 8f) were performed with 25 µM Ub^OG488^, 0.2 µM E1 (UBA1, UBA6, or E1 mutants) in ubiquitylation buffer (50 mM HEPES, pH 7.4, 150 mM NaCl). Reactions were incubated at 37 °C and initiated by adding 5× ATP buffer (25 mM MgCl_2_, pH 7.5, 50 mM ATP). After 2 min, reactions were quenched with non-reducing protein loading buffer, followed by separation on a 4-12% SurePAGE™ Bis-Tris gel with MES running buffer (GenScript Biotech). Gels were first visualized by fluorescence, and then stained using the eStain L1 Protein Staining Device (GenScript Biotech). Both fluorescent gels and Coomassie blue-stained gels were scanned on the ChemiDoc MP Imaging System (Bio-Rad). In the stained gels, the proportion of E1~Ub thioester relative to total E1 was quantified within each lane using Image Lab (version 6.0.1). Data analysis and plotting were performed with GraphPad Prism 9.5. Uncropped gels were provided in Source Data.

### E1–E2 thioester transfer assays

To validate the molecular basis of UBA6 specificity for BIRC6 (as shown in Fig. 1a), E1-BIRC6 thioester transfer assays were performed with 25 µM Ub^OG488^, 0.2 µM E1 (UBA1, UBA6), and 0.2 µM BIRC6 in ubiquitylation buffer (50 mM HEPES, pH 7.4, 150 mM NaCl). Reactions were incubated at 37 °C and initiated by adding 5× ATP buffer (25 mM MgCl_2_, pH 7.5, 50 mM ATP). After 30 min, reactions were quenched with non-reducing protein loading buffer. Samples were then resolved on 3-8% NuPAGE Bis‑Tris gels (Thermo Fisher Scientific).

To validate the molecular basis of UBA6 specificity for BIRC6^UBC^ (as shown in Figs. 1b, 4h-i, Supplementary Fig. 8g), E1–E2 thioester transfer assays were performed with 25 µM Ub^OG488^, 0.2 µM E1 (UBA1, UBA6, or E1 mutants), and 5 µM E2 (UbcH5c, BIRC6^UBC^, or BIRC6^UBCΔinsertion loop^) in ubiquitylation buffer (50 mM HEPES, pH 7.4, 150 mM NaCl). Reactions were incubated at 37 °C and initiated by adding 5× ATP buffer (25 mM MgCl_2_, pH 7.5, 50 mM ATP). For Fig.1b, aliquots were taken after 30 min. For Fig. 4h–i, Supplementary Fig. 8g, aliquots were taken at the following time points: 0, 2, 5, 10, 30 min, and then quenched with non-reducing protein loading buffer. Samples were then resolved on 4-12% SurePAGE™ Bis-Tris gels with MES running buffer (GenScript Biotech).

To evaluate critical structural interfaces (as shown in Fig. 3h), UBA6-E2 thioester transfer assays were performed with 25 µM Ub^OG488^, 0.2 µM UBA6, 5 µM E2 (BIRC6^UBC^ and its mutants) in ubiquitination buffer (50 mM HEPES, pH 7.4, 150 mM NaCl). Reactions were incubated at 37 °C and initiated by adding 5× ATP buffer (25 mM MgCl_2_, pH 7.5, 50 mM ATP). Aliquots were taken at 2 min and quenched with non-reducing protein loading buffer. Samples were then resolved on 4-12% SurePAGE™ Bis‑Tris gels with MES running buffer (GenScript).

All gels were first visualized by fluorescence, and then stained using the eStain L1 Protein Staining Device (GenScript Biotech). Both fluorescent gels and stained gels were scanned on the ChemiDoc MP Imaging System (Bio-Rad). In the stained gels, the proportion of E2~Ub thioester relative to total E2 was quantified within each lane using Image Lab (6.0.1). Data analysis and plotting were performed with GraphPad Prism 9.5. Uncropped gels were provided in Source Data.

### Bio-layer interferometry (BLI) assays

Bio-Layer Interferometry (BLI) experiments were performed on an Octet® R8 System (SARTORIUS) using Anti-Penta-His (His1K) biosensors (SARTORIUS, 18-5120). These biosensors were pre-immobilized with Qiagen Penta-HIS antibody (Qiagen, 34660), which specifically binds to the His-tag without an additional activation step.

For sample preparation, ligands were prepared in BLI buffer (50 mM HEPES, pH 7.4, 150 mM NaCl, 0.05% Tween-20, 10 mM imidazole) at the following concentrations: UBA1/UBA1-Ub at 25 µg/mL and UBA6/UBA6-Ub at 50 µg/mL. Analytes (BIRC6^UBC ^or BIRC6^UBCΔinsertion loop^) were three-fold serially diluted in BLI buffer to final concentrations of 3240, 1080, 360, 120, and 40 nM.

For BLI measurements, His-tagged ligands were first immobilized onto His1K biosensors by incubating the sensors in the ligand solutions for 180 s. Reference sensors were only exposed to buffer (no ligand). In the association step, loaded sensors were exposed to serially diluted analyte solutions for 120 s. With the same association times, the biosensors loaded with the same ligand were exposed to the reference well (containing buffer without ligand) to subtract the buffer background. Reference sensors were exposed to the highest analyte concentration (3240 nM BIRC6^UBC^ or BIRC6^UBCΔinsertion loop^) for nonspecific binding control. In the dissociation step, all sensors were exposed to the buffer for 180 s. All measurements were carried out at 25 °C with orbital shaking at 1,000 rpm, and data were collected at 5 Hz.

Data were processed using the Octet BLI Analysis 12.0 software (Sartorius). Reference well subtraction was first applied during the pre-processing step. Then, the Y-axis was aligned to the “Average of Baseline Step”, followed by high-frequency noise reduction using Savitzky-Golay filtering. The steady-state binding response (Req) was calculated as the average signal over the last 5-10 s of the association step. Req values were plotted against analyte concentration and fitted with a 1:1 binding model using global fitting of the Rmax parameters to determine equilibrium dissociation constants (K_D_). Data plotting was performed using GraphPad Prism 9.5. The Source Data for the BLI experiments are provided as Excel files in the attachment.

### Reporting summary

Further information on research design is available in the Nature Portfolio Reporting Summary linked to this article.

## Supplementary information

Supplementary Information

Reporting Summary

Transparent Peer Review file

## Source data

Source Data

## Data Availability

The cryo-EM maps generated in this study have been deposited in the Electron Microscopy Data Bank (EMDB) under accession codes EMD‑66842 (singly loaded UBA6-BIRC6^UBC^-Ub^T^ complex) [https://www.ebi.ac.uk/emdb/entry/EMD-66842] and EMD‑66843 (doubly loaded UBA6-BIRC6^UBC^-Ub^T^-Ub^A^ complex) [https://www.ebi.ac.uk/emdb/entry/EMD-66843]. The atomic models have been deposited in the Protein Data Bank (PDB) under accession codes 9XGB (singly loaded UBA6-BIRC6^UBC^-Ub^T^ complex) [https://doi.org/10.2210/pdb9XGB/pdb] and 9XGC (doubly loaded UBA6-BIRC6^UBC^-Ub^T^-Ub^A^ complex) [https://doi.org/10.2210/pdb9XGC/pdb]. Source data are provided with this paper.

## References

[1] Komander, D. & Rape, M. The Ubiquitin Code. *Annu. Rev. Biochem.* **81**, 203–229 (2012). 10.1146/annurev-biochem-060310-170328 22524316

[2] Cappadocia, L. & Lima, C. D. Ubiquitin-like Protein Conjugation: Structures, Chemistry, and Mechanism. *Chem. Rev.* **118**, 889–918 (2018). 10.1021/acs.chemrev.6b00737 PMC5815371 28234446

[3] Bedford, L., Lowe, J., Dick, L. R., Mayer, R. J. & Brownell, J. E. Ubiquitin-like protein conjugation and the ubiquitin–proteasome system as drug targets. *Nat. Rev. Drug Discov.* **10**, 29–46 (2011). 10.1038/nrd3321 PMC7097807 21151032

[4] Pickart, C. M. & Eddins, M. J. Ubiquitin: structures, functions, mechanisms. *Biochim. Biophys. Acta* **1695**, 55–72 (2004). 10.1016/j.bbamcr.2004.09.019 15571809

[5] Dye, B. T. & Schulman, B. A. Structural mechanisms underlying posttranslational modification by ubiquitin-like proteins. *Annu. Rev. Biophys. Biomol. Struct.* **36**, 131–150 (2007). 10.1146/annurev.biophys.36.040306.132820 17477837

[6] Hershko, A. & Ciechanover, A. The ubiquitin system. *Annu. Rev. Biochem.* **67**, 425–479 (1998). 10.1146/annurev.biochem.67.1.425 9759494

[7] Buetow, L. & Huang, D. T. Structural insights into the catalysis and regulation of E3 ubiquitin ligases. *Nat. Rev. Mol. Cell Biol.* **17**, 626–642 (2016). 10.1038/nrm.2016.91 PMC6211636 27485899

[8] Stewart, M. D., Ritterhoff, T., Klevit, R. E. & Brzovic, P. S. E2 enzymes: more than just middle men. *Cell Res.* **26**, 423–440 (2016). 10.1038/cr.2016.35 PMC4822130 27002219

[9] Schulman, B. A. & Wade Harper, J. Ubiquitin-like protein activation by E1 enzymes: the apex for downstream signalling pathways. *Nat. Rev. Mol. Cell Biol.* **10**, 319–331 (2009). 10.1038/nrm2673 PMC2712597 19352404

[10] Jin, J., Li, X., Gygi, S. P. & Harper, J. W. Dual E1 activation systems for ubiquitin differentially regulate E2 enzyme charging. *Nature* **447**, 1135–1138 (2007). 10.1038/nature05902 17597759

[11] Truongvan, N., Li, S., Misra, M., Kuhn, M. & Schindelin, H. Structures of UBA6 explain its dual specificity for ubiquitin and FAT10. *Nat. Commun.* **13**, 4789 (2022). 10.1038/s41467-022-32040-6 PMC9378703 35970836

[12] Rape, M. Ubiquitylation at the crossroads of development and disease. *Nat. Rev. Mol. Cell Biol.* **19**, 59–70 (2018). 10.1038/nrm.2017.83 28928488

[13] Kochanczyk, T. et al. Structural basis for transthiolation intermediates in the ubiquitin pathway. *Nature* **633**, 216–223 (2024). 10.1038/s41586-024-07828-9 PMC11374688 39143218

[14] Yuan, L., Lv, Z., Adams, M. J. & Olsen, S. K. Crystal structures of an E1–E2–ubiquitin thioester mimetic reveal molecular mechanisms of transthioesterification. *Nat. Commun.* **12**, 2370 (2021). 10.1038/s41467-021-22598-y PMC8062481 33888705

[15] Williams, K. M. et al. Structural insights into E1 recognition and the ubiquitin-conjugating activity of the E2 enzyme Cdc34. *Nat. Commun.* **10**, 3296 (2019). 10.1038/s41467-019-11061-8 PMC6656757 31341161

[16] Lv, Z. et al. S. pombe Uba1-Ubc15 Structure Reveals a Novel Regulatory Mechanism of Ubiquitin E2 Activity. *Mol. Cell* **65**, 699–714 (2017). 10.1016/j.molcel.2017.01.008 PMC5319395 28162934

[17] Olsen, S. K. & Lima, C. D. Structure of a ubiquitin E1–E2 complex: insights to E1–E2 thioester transfer. *Mol. Cell* **49**, 884–896 (2013). 10.1016/j.molcel.2013.01.013 PMC3625138 23416107

[18] Lee, I. & Schindelin, H. Structural Insights into E1-Catalyzed Ubiquitin Activation and Transfer to Conjugating Enzymes. *Cell* **134**, 268–278 (2008). 10.1016/j.cell.2008.05.046 18662542

[19] Wallace, I. et al. Insights into the ISG15 transfer cascade by the UBE1L activating enzyme. *Nat. Commun.* **14**, 7970 (2023). 10.1038/s41467-023-43711-3 PMC10693564 38042859

[20] Afsar, M. et al. Cryo-EM structures of Uba7 reveal the molecular basis for ISG15 activation and E1–E2 thioester transfer. *Nat. Commun.* **14**, 4786 (2023). 10.1038/s41467-023-39780-z PMC10409785 37553340

[21] Nayak, A. et al. Cryo-EM structures reveal the molecular mechanism of SUMO E1–E2 thioester transfer. *Nat. Struct. Mol. Biol.* **32**, 2441–2453 (2025). 10.1038/s41594-025-01681-8 PMC12700794 40999065

[22] Hauser, H.-P., Bardroff, M., Pyrowolakis, G. & Jentsch, S. A Giant Ubiquitin-conjugating Enzyme Related to IAP Apoptosis Inhibitors. *J. Cell Biol.* **141**, 1415–1422 (1998). 10.1083/jcb.141.6.1415 PMC2132795 9628897

[23] Chen, Z. et al. A Human IAP-Family Gene, Apollon, Expressed in Human Brain Cancer Cells. *Biochem. Biophys. Res. Commun.* **264**, 847–854 (1999). 10.1006/bbrc.1999.1585 10544019

[24] Yip, M. C. J., Sedor, S. F. & Shao, S. Mechanism of client selection by the protein quality-control factor UBE2O. *Nat. Struct. Mol. Biol.* **29**, 774–780 (2022). 10.1038/s41594-022-00807-6 PMC9526450 35915257

[25] Silke, J. & Meier, P. Inhibitor of Apoptosis (IAP) Proteins-Modulators of Cell Death and Inflammation. *Cold Spring Harb. Perspect. Biol.* **5**, a008730–a008730 (2013). 10.1101/cshperspect.a008730 PMC3552501 23378585

[26] Dietz, L. et al. Structural basis for SMAC-mediated antagonism of caspase inhibition by the giant ubiquitin ligase BIRC6. *Science* **379**, 1112–1117 (2023). 10.1126/science.ade8840 36758106

[27] Hunkeler, M., Jin, C. Y. & Fischer, E. S. Structures of BIRC6-client complexes provide a mechanism of SMAC-mediated release of caspases. *Science* **379**, 1105–1111 (2023). 10.1126/science.ade5750 36758104

[28] Ehrmann, J. F. et al. Structural basis for regulation of apoptosis and autophagy by the BIRC6/SMAC complex. *Science* **379**, 1117–1123 (2023). 10.1126/science.ade8873 36758105

[29] Bartke, T., Pohl, C., Pyrowolakis, G. & Jentsch, S. Dual role of BRUCE as an antiapoptotic IAP and a chimeric E2/E3 ubiquitin ligase. *Mol. Cell* **14**, 801–811 (2004). 10.1016/j.molcel.2004.05.018 15200957

[30] Hao, Y. et al. Apollon ubiquitinates SMAC and caspase-9, and has an essential cytoprotection function. *Nat. Cell Biol.* **6**, 849–860 (2004). 10.1038/ncb1159 15300255

[31] Liu, S. S. et al. Molecular mechanisms underlying the BIRC6-mediated regulation of apoptosis and autophagy. *Nat. Commun.* **15**, 891 (2024). 10.1038/s41467-024-45222-1 PMC10827748 38291026

[32] Tsherniak, A. et al. Defining a Cancer Dependency Map. *Cell* **170**, 564–576.e516 (2017). 10.1016/j.cell.2017.06.010 PMC5667678 28753430

[33] Haas, A. L., Bright, P. M. & Jackson, V. E. Functional diversity among putative E2 isozymes in the mechanism of ubiquitin-histone ligation. *J. Biol. Chem.* **263**, 13268–13275 (1988). 2843516

[34] Pickart, C. M., Kasperek, E. M., Beal, R. & Kim, A. Substrate properties of site-specific mutant ubiquitin protein (G76A) reveal unexpected mechanistic features of ubiquitin-activating enzyme (E1). *J. Biol. Chem.* **269**, 7115–7123 (1994). 8125920

[35] Kochanczyk, T., Fishman, M. & Lima, C. D. Chemical Tools for Probing the Ub/Ubl Conjugation Cascades. *ChemBioChem* **26**, e202400659 (2025). 10.1002/cbic.202400659 PMC11727022 39313481

[36] Henneberg, L. T. & Schulman, B. A. Decoding the messaging of the ubiquitin system using chemical and protein probes. *Cell Chem. Biol.* **28**, 889–902 (2021). 10.1016/j.chembiol.2021.03.009 PMC7611516 33831368

[37] Yang, Z., Xiao, Y., Shi, Y. & Liu, L. Advances in the chemical synthesis of human proteoforms. *Sci. China Life Sci.* **68**, 2515–2549 (2025). 10.1007/s11427-024-2860-5 40210795

[38] Ai, H. et al. Mechanism of nucleosomal H2A K13/15 monoubiquitination and adjacent dual monoubiquitination by RNF168. *Nat. Chem. Biol.* **21**, 668–680 (2024). 10.1038/s41589-024-01750-x 39394267

[39] Ai, H. et al. Synthetic E2-Ub-nucleosome conjugates for studying nucleosome ubiquitination. *Chem* **9**, 1221–1240 (2023).

[40] Tong, Z. et al. Structural basis for E4 enzyme Ufd2-catalyzed K48/K29 branched ubiquitin chains. *Nat. Chem. Biol.* **22**, 239–248 (2025). 10.1038/s41589-025-01985-2 40817136

[41] Pan, M. et al. Structural insights into Ubr1-mediated N-degron polyubiquitination. *Nature* **600**, 334–338 (2021). 10.1038/s41586-021-04097-8 PMC8798225 34789879

[42] Horn-Ghetko, D. et al. Ubiquitin ligation to F-box protein targets by SCF–RBR E3–E3 super-assembly. *Nature* **590**, 671–676 (2021). 10.1038/s41586-021-03197-9 PMC7904520 33536622

[43] Baek, K. et al. NEDD8 nucleates a multivalent cullin-RING-UBE2D ubiquitin ligation assembly. *Nature* **578**, 461–466 (2020). 10.1038/s41586-020-2000-y PMC7050210 32051583

[44] Hann, Z. S. et al. Structural basis for adenylation and thioester bond formation in the ubiquitin E1. *Proc. Natl. Acad. Sci. Usa.* **116**, 15475–15484 (2019). 10.1073/pnas.1905488116 PMC6681703 31235585

[45] Yang, Z. et al. Molecular basis of SIFI activity in the integrated stress response. *Nature* **643**, 1117–1126 (2025). 10.1038/s41586-025-09074-z PMC12286842 40328314

[46] Streich, F. C. Jr & Lima, C. D. Capturing a substrate in an activated RING E3/E2-SUMO complex. *Nature* **536**, 304–308 (2016). 10.1038/nature19071 PMC5019495 27509863

[47] Maiwald, S. A. et al. TRIP12 structures reveal HECT E3 formation of K29 linkages and branched ubiquitin chains. *Nat. Struct. Mol. Biol.* **32**, 1766–1775 (2025). 10.1038/s41594-025-01561-1 PMC12440805 40419785

[48] Weber, A. et al. A Linear Diubiquitin-Based Probe for Efficient and Selective Detection of the Deubiquitinating Enzyme OTULIN. *Cell Chem. Biol.* **24**, 1299–1313 e1297 (2017). 10.1016/j.chembiol.2017.08.006 PMC5658516 28919039

[49] Lu, L. et al. Met1-specific motifs conserved in OTUB subfamily of green plants enable rice OTUB1 to hydrolyse Met1 ubiquitin chains. *Nat. Commun.* **13**, 4672 (2022). 10.1038/s41467-022-32364-3 PMC9363410 35945250

[50] Li, Y., Varejão, N. & Reverter, D. Structural basis for the SUMO protease activity of the atypical ubiquitin-specific protease USPL1. *Nat. Commun*. **13**, 1819 (2022). 10.1038/s41467-022-29485-0 PMC8983731 35383180

[51] Mulder, M. P. C. et al. A cascading activity-based probe sequentially targets E1–E2–E3 ubiquitin enzymes. *Nat. Chem. Biol.* **12**, 523–530 (2016). 10.1038/nchembio.2084 PMC5108872 27182664

[52] Riechmann, C. et al. UBA6 specificity for ubiquitin E2 conjugating enzymes reveals a priority mechanism of BIRC6. *Nat. Struct. Mol. Biol.* **33**, 464–478 (2025). 10.1038/s41594-025-01717-z PMC12999496 41350950

[53] Nayak, D. et al. Cryo-EM structures of UBA6 reveal mechanisms of E1–E2 specificity and dual FAT10/ubiquitin thioester transfer. *Nat. Commun.* **17**, 3302 (2026). 10.1038/s41467-026-69882-3 PMC13065764 41764162

[54] Sheng, Y. et al. A Human Ubiquitin Conjugating Enzyme (E2)-HECT E3 Ligase Structure-function Screen. *Mol. Cell. Proteom.* **11**, 329–341 (2012). 10.1074/mcp.O111.013706 PMC3412965 22496338

[55] Zivanov, J. et al. New tools for automated high-resolution cryo-EM structure determination in RELION-3. *eLife* **7**, e42166 (2018). 10.7554/eLife.42166 PMC6250425 30412051

[56] Zheng, S. Q. et al. MotionCor2: anisotropic correction of beam-induced motion for improved cryo-electron microscopy. *Nat. Methods* **14**, 331–332 (2017). 10.1038/nmeth.4193 PMC5494038 28250466

[57] Zhang, K. Gctf: Real-time CTF determination and correction. *J. Struct. Biol.* **193**, 1–12 (2016). 10.1016/j.jsb.2015.11.003 PMC4711343 26592709

[58] Vijay-Kumar, Bugg, S. & Cook, C. E. W.J. Structure of Ubiquitin Refined at 1.43 Å Resolution. *J. Mol. Biol.* **194**, 531–544 (1987). 10.1016/0022-2836(87)90679-6 3041007

[59] Pettersen, E. F. et al. UCSF ChimeraX: Structure visualization for researchers, educators, and developers. *Protein Sci.* **30**, 70–82 (2021). 10.1002/pro.3943 PMC7737788 32881101

[60] Emsley, P., Lohkamp, B., Scott, W. G. & Cowtan, K. Features and development of Coot. *Acta Crystallogr., Sect. D: Biol. Crystallogr.* **66**, 486–501 (2010). 10.1107/S0907444910007493 PMC2852313 20383002

[61] Adams, P. D. et al. PHENIX: a comprehensive Python-based system for macromolecular structure solution. *Acta Crystallogr., Sect. D: Biol. Crystallogr.* **66**, 213–221 (2010). 10.1107/S0907444909052925 PMC2815670 20124702

