# Supplementary material for: Steric Gating Contributes to the Preferential Ubiquitin Charging of BIRC6 by UBA6 versus UBA1
Source: Nat Commun. 2026 Aug 29;17:10349. doi: 10.1038/s41467-026-75898-6 (PMC13623948; doi:10.1038/s41467-026-75898-6)
Supplement: Supplementary file 1 [file 41467_2026_75898_MOESM1_ESM.pdf]

Supplementary Information

1. Supplementary Figures

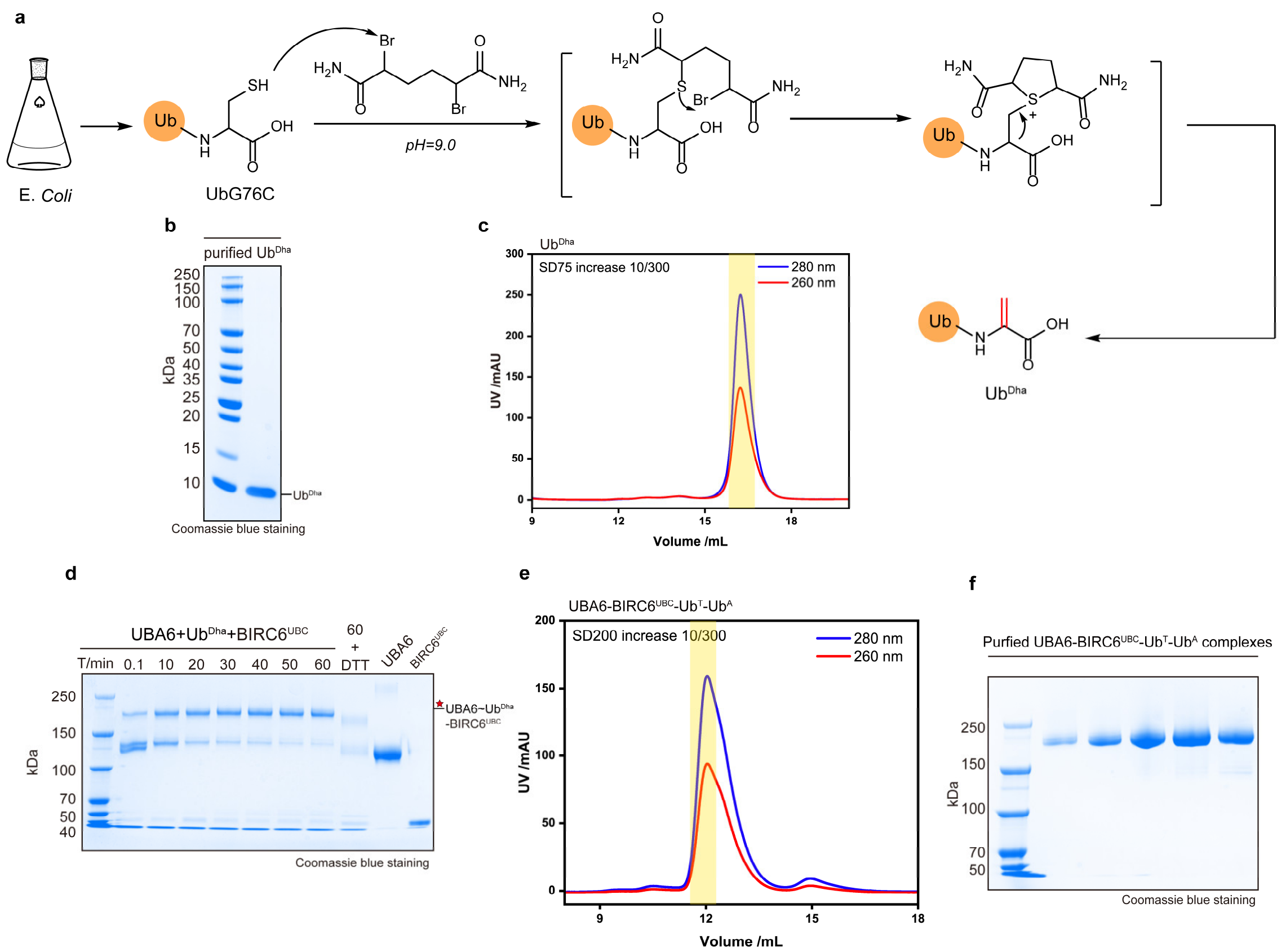

Supplementary Fig. 1 | Semi-synthesis of Ub^{Dha} probe and preparation of UBA6-BIRC6^{UBC}-Ub^{Dha} complex. a, Schematic of the semi-synthesis of Ub^{Dha} from recombinant UbG76C. **b**, SDS-PAGE analysis of purified Ub^{Dha}. **c**, Representative size-exclusion chromatography (SEC) of Ub^{Dha} purification. **d**, SDS-PAGE analysis of the preparation of UBA6-BIRC6^{UBC}-Ub^{Dha} complex at indicated times. Time points=0.1, 10, 20, 30, 40, 50, 60min. **e**, Representative size-exclusion chromatography of UBA6-BIRC6^{UBC}-Ub^{Dha} complex. **f**, SDS-PAGE analysis of UBA6-BIRC6^{UBC}-Ub^{Dha} complex.

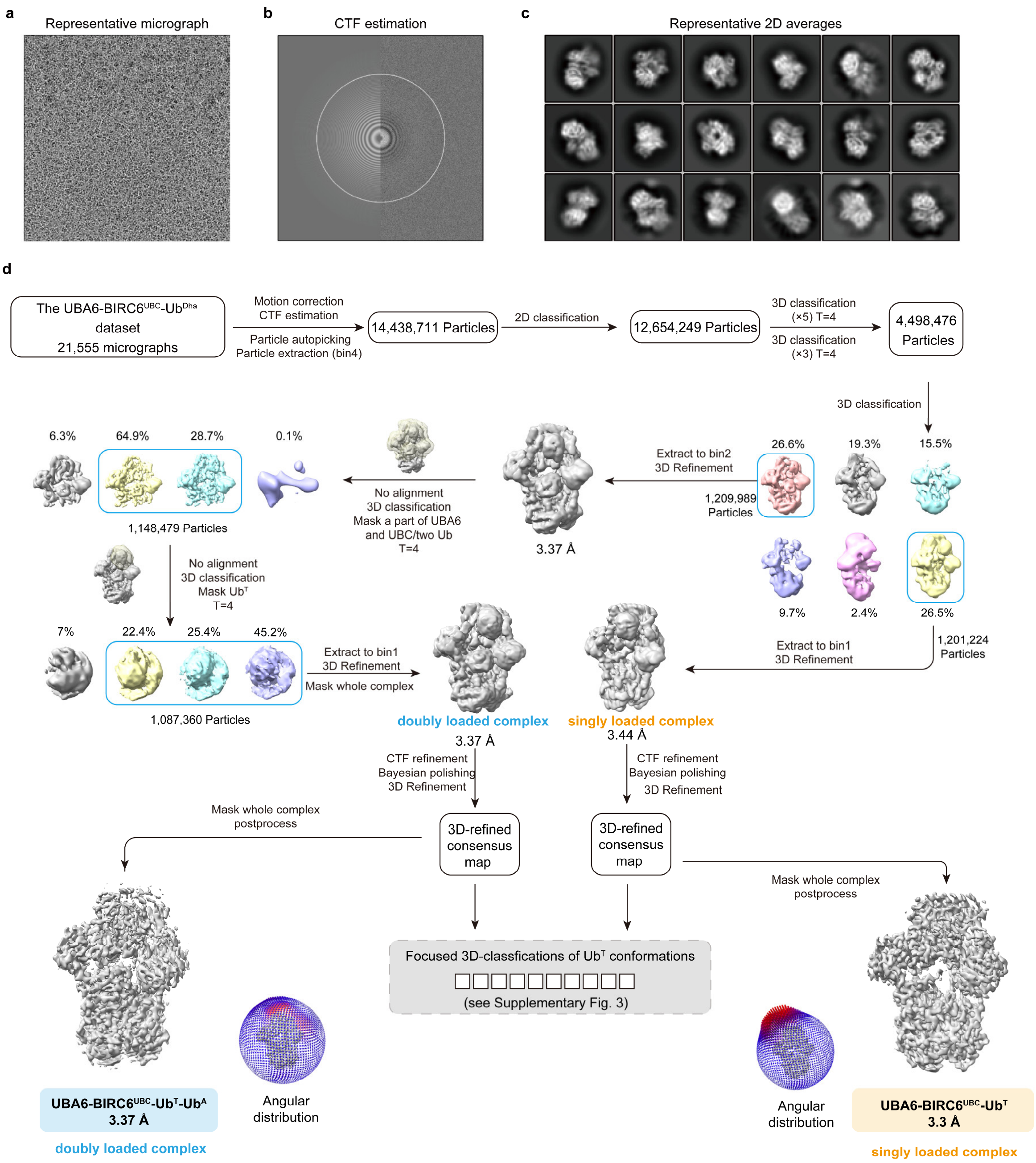

Supplementary Fig. 2 | Cryo-EM data collection and processing of UBA6-BIRC6^{UBC}-Ub^{Dha} complexes. **a**, A representative micrograph from the UBA6-BIRC6^{UBC}-Ub^{Dha} dataset. **b**, CTF estimation of the micrograph shown in **a**. **c**, Representative 2D averages of the UBA6-BIRC6^{UBC}-Ub^{Dha} complex. **d**, Cryo-EM processing flowchart of the UBA6-BIRC6^{UBC}-Ub^{Dha} dataset. The distribution of the Euler angles within UBA6-BIRC6^{UBC}-Ub^{T}-Ub^{A} complex and UBA6-BIRC6^{UBC}-Ub^{T} complex have been shown.

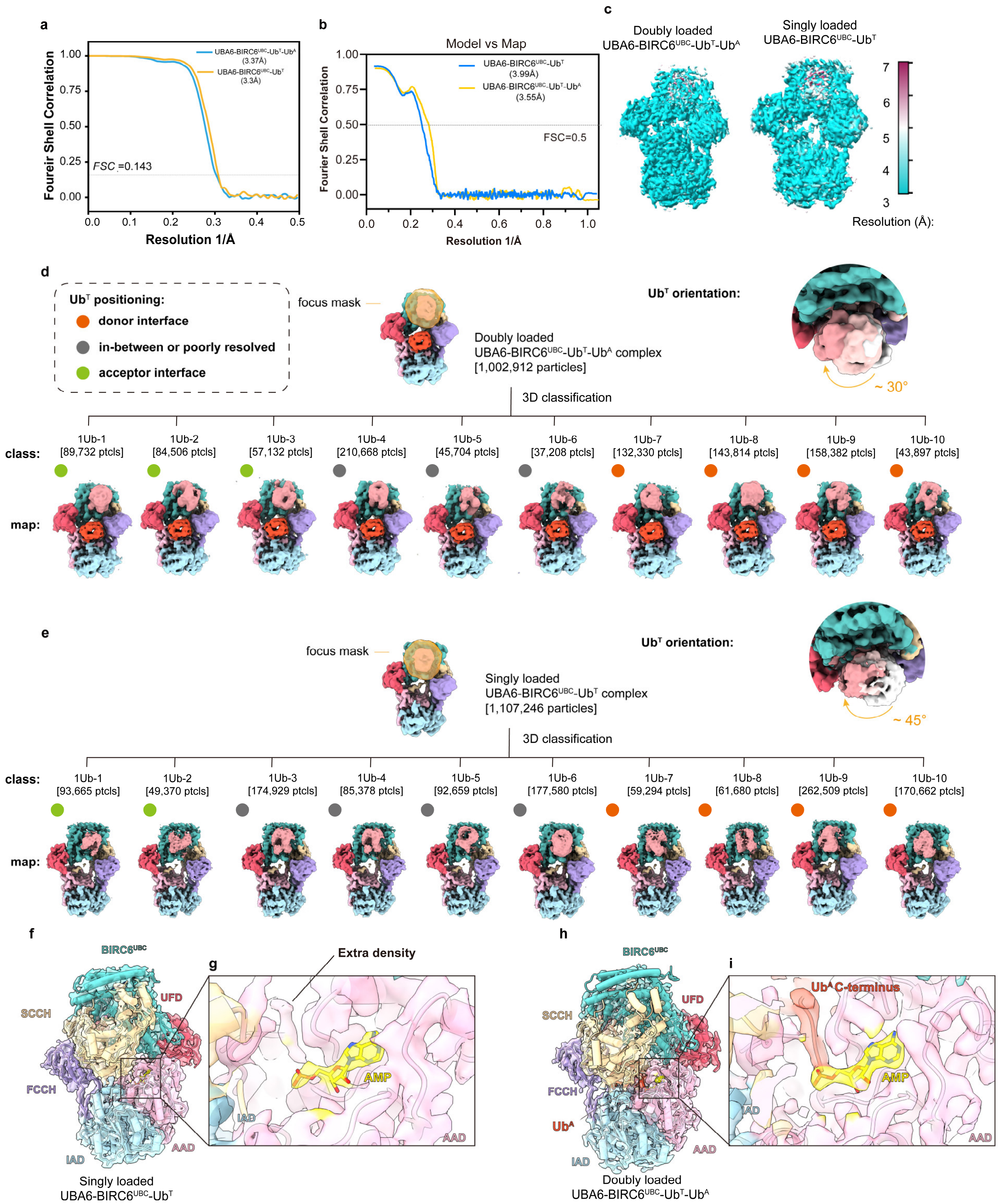

Supplementary Fig. 3 | Cryo-EM density of the UBA6-BIRC6^{UBC}-Ub^{Dha} complexes and focused Ub^{T} in UBA6-BIRC6^{UBC}-Ub^{Dha} complexes. a, Fourier shell correlation (FSC) curves of the masked map after Relion postprocessing. The resolution was determined by the FSC=0.143 criterion. **b**, Map-versus-model evaluation of the UBA6-BIRC6^{UBC}-Ub^{T}-Ub^{A} and UBA6-BIRC6^{UBC}-Ub^{T} complexes. **c**, The local resolution of the density of the map calculated by Relion. **d-e**, Cryo-EM reconstructions and corresponding models of Ub^{T} states resulting from 3D classification without image realignment. A focus mask was applied to the Ub^{T} region in both doubly- and single-loaded complexes. The

corresponding orientation of Ub^T was shown at right. **f-i**, Density and model views of the ATP catalytic pocket of UBA6 in the singly and doubly loaded UBA6-BIRC6 complexes. In both structures, the pocket contains density that is well fitted by AMP, and extra density adjacent to AMP likely corresponds to the C-terminal tail of adenylated ubiquitin.

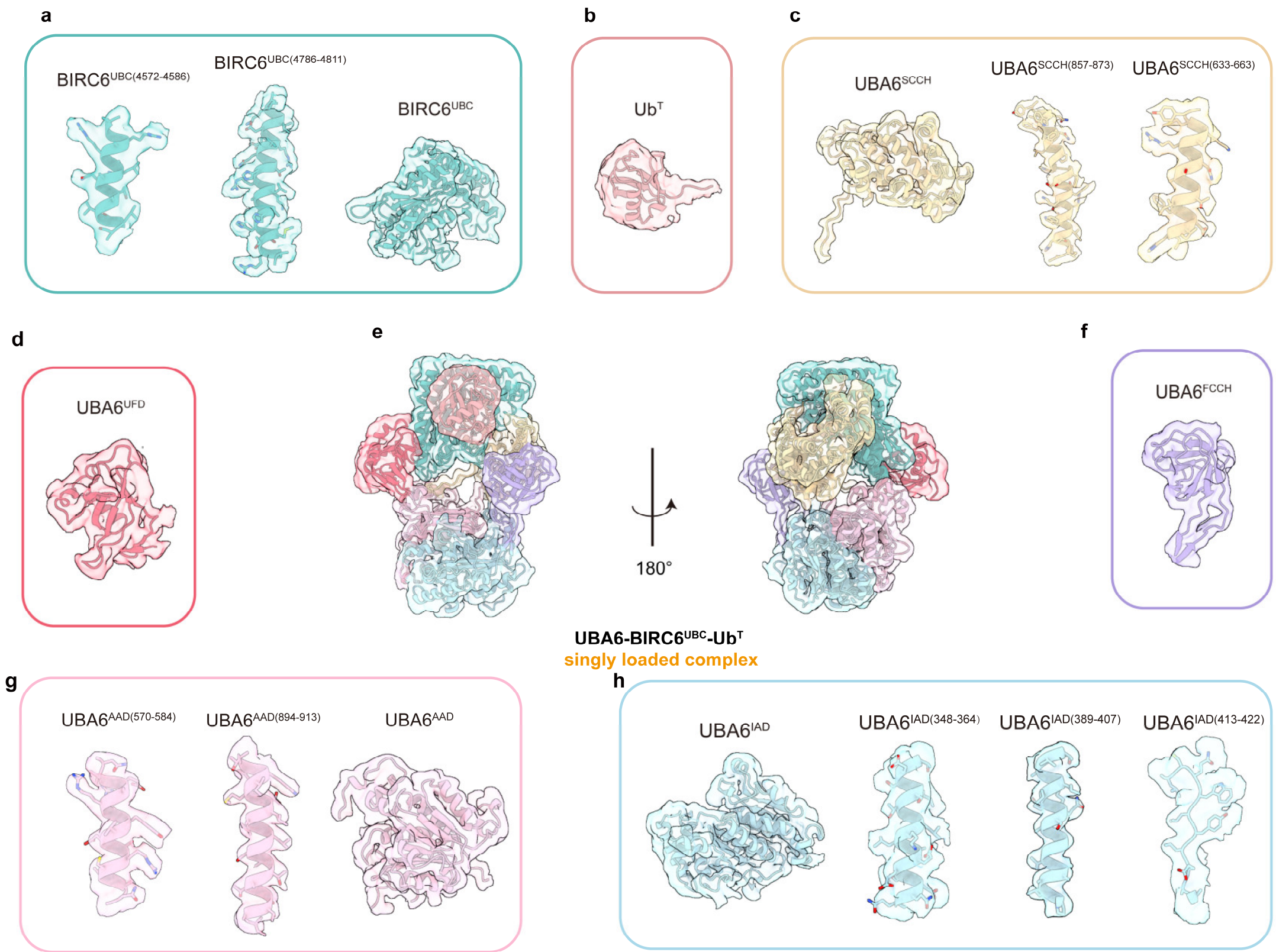

Supplementary Fig. 4 | Cryo-EM density and model of the singly loaded UBA6-BIRC6^{UBC}-Ub^{T} complex. a-h. Representative regions of cryo-EM densities for BIRC6^{UBC} (4520-4857) (**a**), Ub^{T} (1-76) (**b**), UBA6^{SCCH} (613-888) (**c**), UBA6^{UFD} (943-1051) (**d**), overall structure of singly loaded UBA6-BIRC6^{UBC}-Ub^{T} complex (**e**), UBA6^{FCCH} (203-297) (**f**), UBA6^{AAD} (430-612, 889-942) (**g**), UBA6^{IAD} (40-202, 298-429) (**h**).

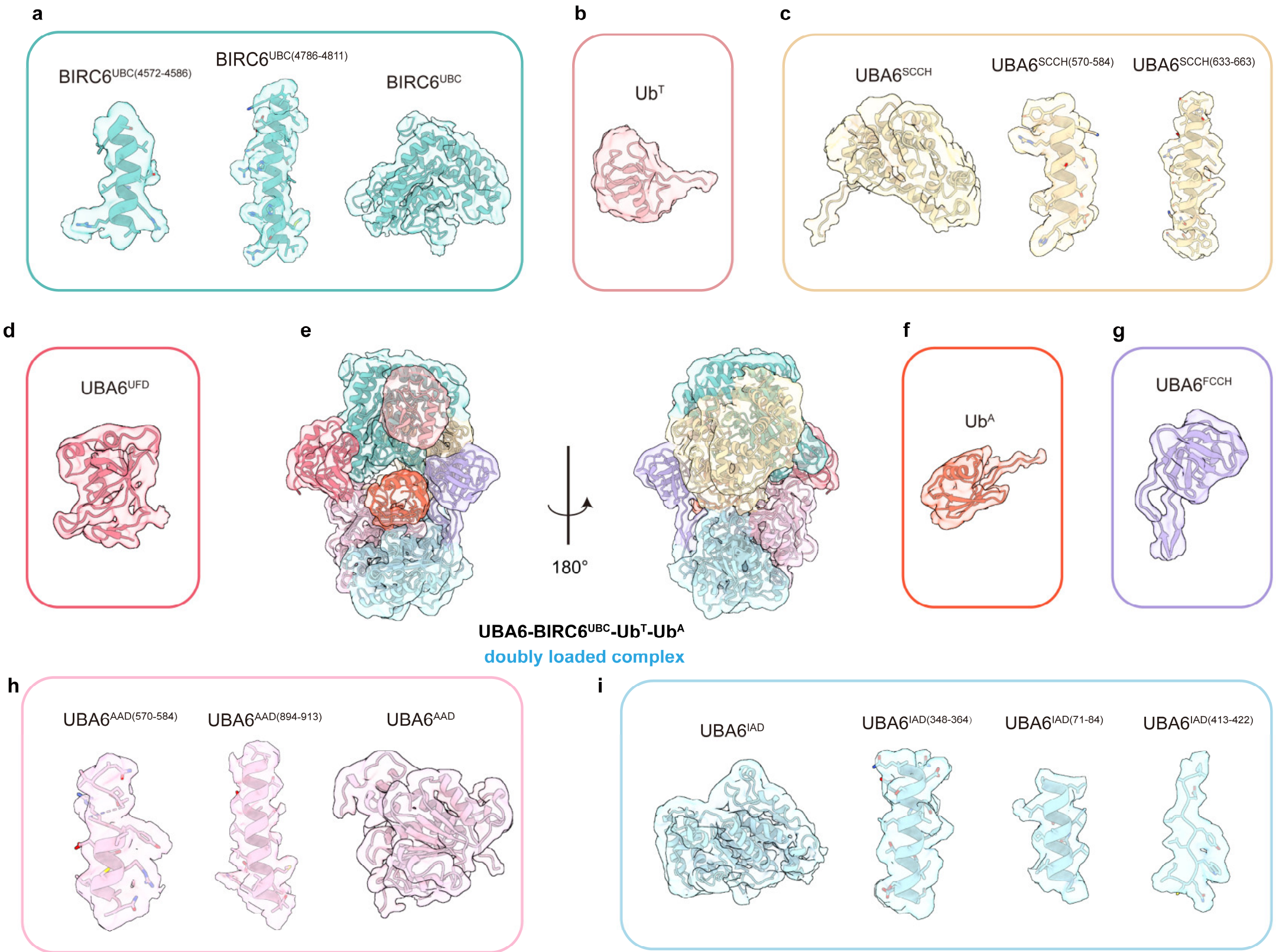

Supplementary Fig. 5 | Cryo-EM density and model of the doubly loaded UBA6-BIRC6^{UBC}-Ub^{T}-Ub^{A} complex. a-i. Representative regions of cryo-EM densities for BIRC6^{UBC} (4520-4857) (**a**), Ub^{T} (1-76) (**b**), UBA6^{SCCH} (613-888) (**c**), UBA6^{UFD} (943-1051) (**d**), overall structure of doubly loaded UBA6-BIRC6^{UBC}-Ub^{T} complex (**e**), Ub^{T} (1-76) (**f**), UBA6^{FCCH} (203-297) (**g**), UBA6^{AAD} (430-612, 889-942) (**h**), UBA6^{IAD} (40-202, 298-429) (**i**).

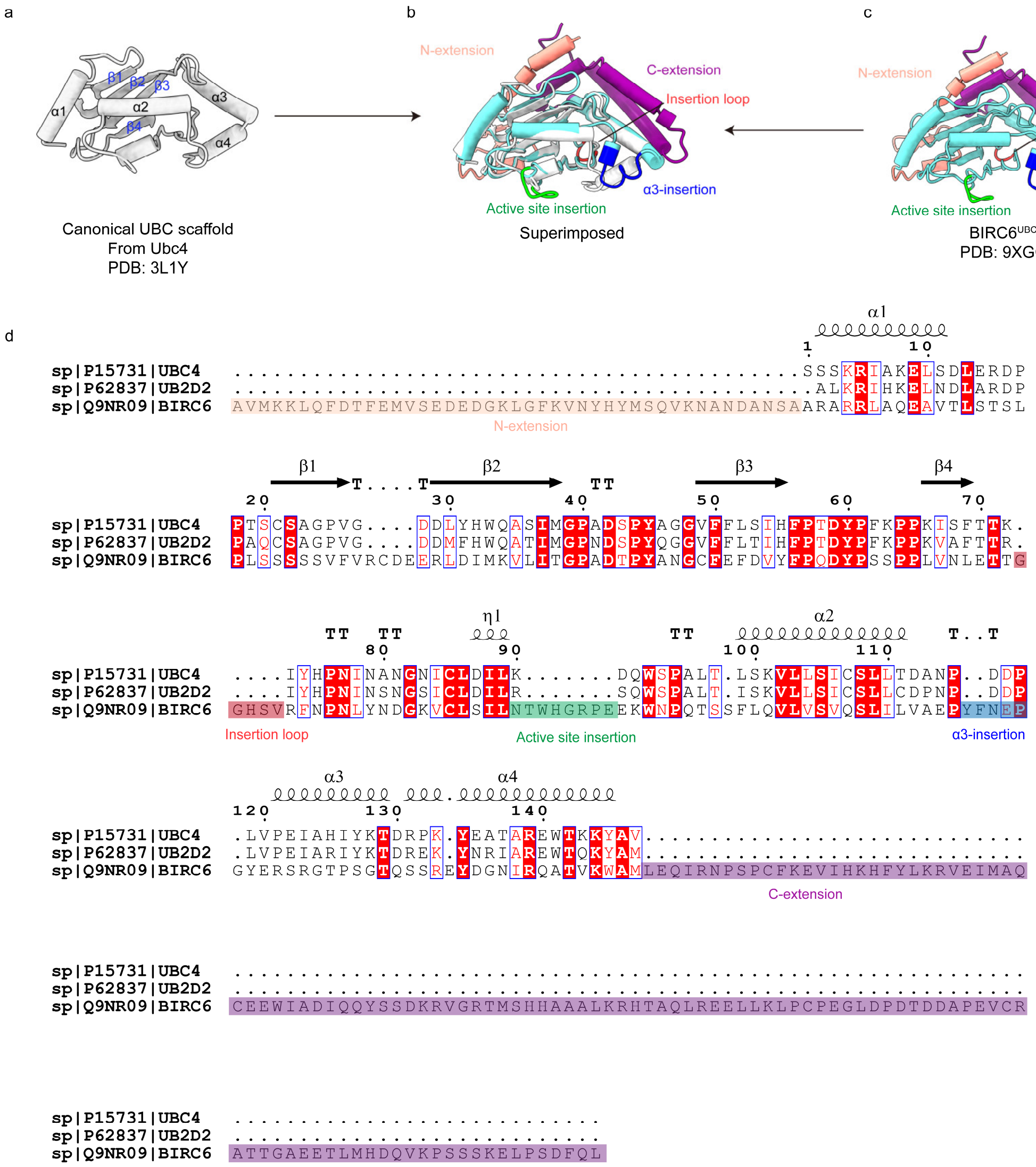

Supplementary Fig. 6 | Comparison of the canonical UBC domain and BIRC6' s UBC domain. a-c, Cartoon depictions of the canonical UBC scaffold from Ubc4 (**a**, PDB: 3L1Y) and the BIRC6's UBC domain (**c**, PDB: 3CEG). **b**, Structural alignment of the crystal structure of Ubc4 and BIRC6^{UBC} determined in this study. **d**, Sequence alignment of representative UBC domains, with unique secondary structures from BIRC6^{UBC} highlighted.

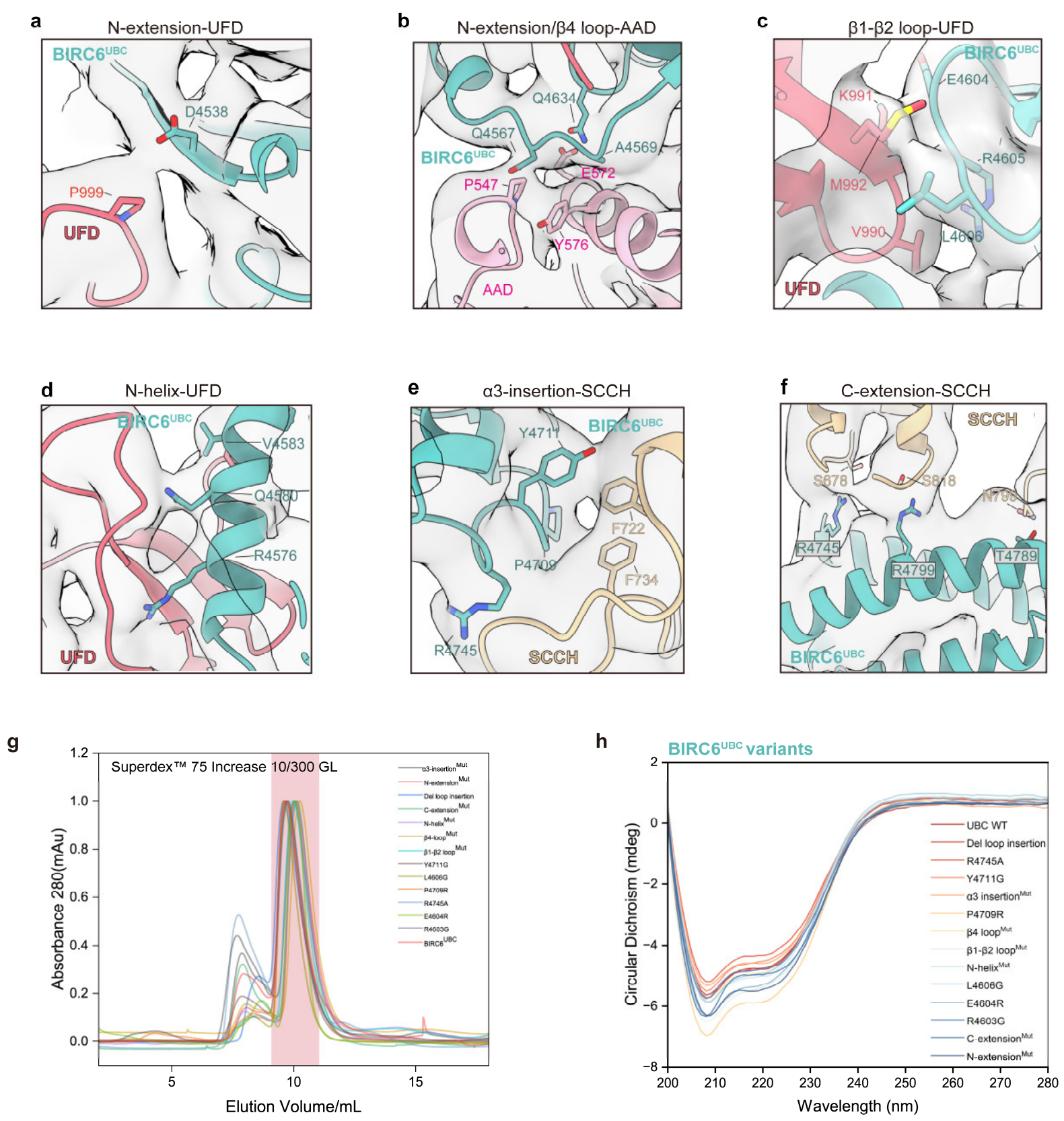

Supplementary Fig. 7 | Analysis of the interactions between UBA6 and BIRC6^{UBC}. a, Close-up view of the interface between BIRC6^{UBC} N-extension (residue D4538) and UBA6^{UFD} (residue P999). **b**, Close-up view of the interface between BIRC6^{UBC} N-extension and β4 loop (residues Q4634, Q4567, A4569) and UBA6^{AAD} (Y576, P547, E572). **c**, Close-up views of the interface between BIRC6^{UBC} β1-β2 loop (residues E4604, R4605, L4606) and UBA6^{UFD} (residues V990, K991, M992). **d**, Close-up view of the interface between the BIRC6^{UBC} N-helix (residues R4576, Q4580, V4583) and UBA6^{UFD} (residue V997). **e**, Close-up views of the interface between the BIRC6^{UBC} α3-insertion (residues P4709, Y4711, R4745) and UBA6^{SCCH} (residues F722, F734). **f**, Close-up views of the interface of the BIRC6^{UBC} C-extension (residues R4745, R4799, T4789) and UBA6^{SCCH} (residues S678, S818, N799). Cryo-EM density and the corresponding structural model are shown, with interacting residues represented as sticks. **g**, Size exclusion chromatography (SEC) profiles of all BIRC6^{UBC} mutants. **h**, Circular dichroism (CD) spectra of all BIRC6^{UBC} mutants.

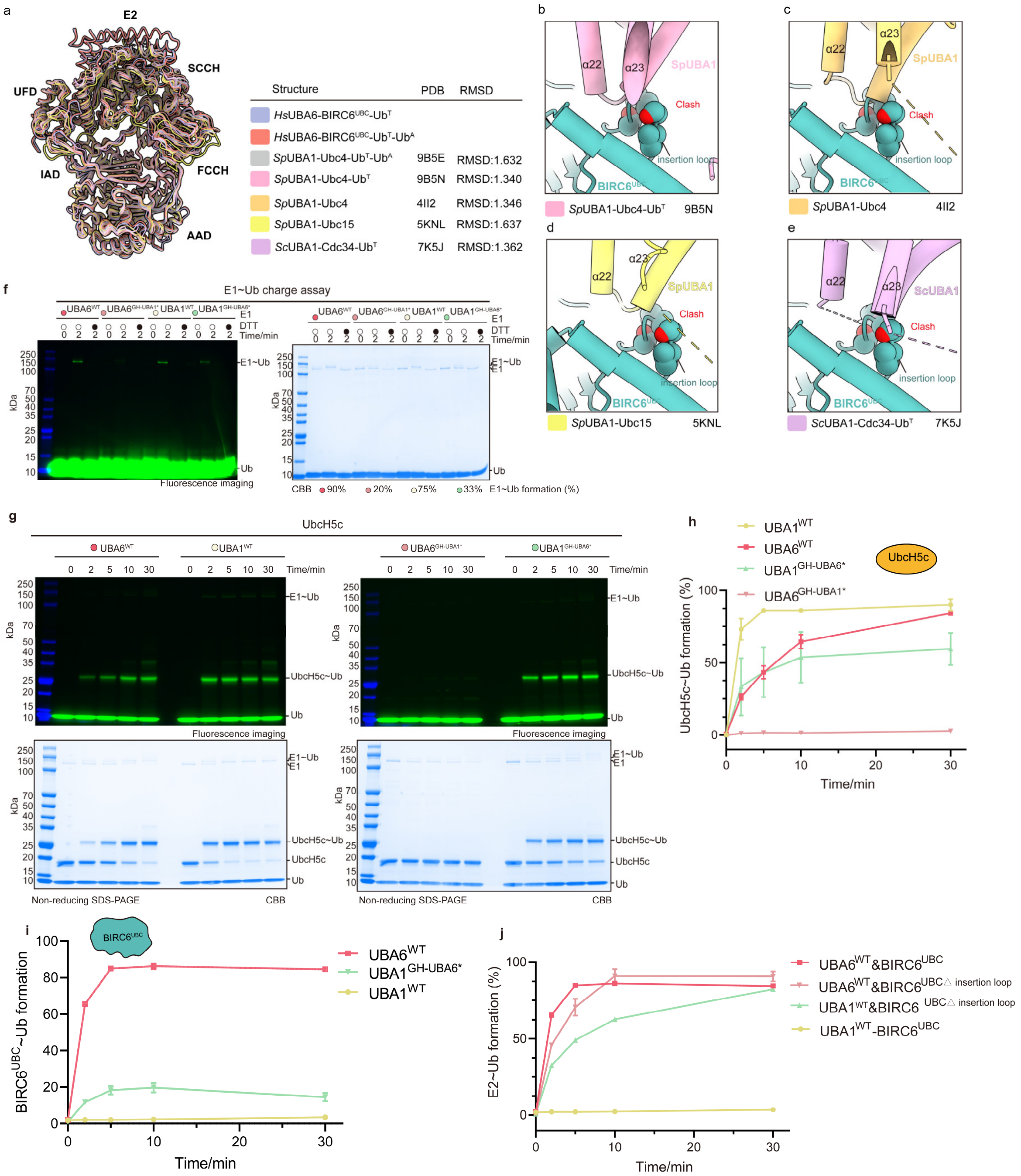

Supplementary Fig. 8 | Structural analysis and validation of the molecular basis of UBA6 specificity for BIRC6^{UBC}. a, Structural alignment of the UBA6-BIRC6^{UBC}-Ub complexes (this study) with previously determined UBA1-E2-Ub structures. **b-e**, Structural alignments reveal steric clashes between the insertion loop of BIRC6^{UBC} and the α22-α23 helix of UBA1 across different UBA1-E2 complexes (PDB codes indicated). **f**, E1~Ub charging assay comparing UBA6^{WT}, UBA1^{WT}, UBA6$^{GH\text{-}UBA1*}$ and UBA1$^{GH\text{-}UBA6*}$. Reaction time: 2 min. **g**, E1-E2 transthioesterification assays using UBA6^{WT}, UBA1^{WT}, UBA6$^{GH\text{-}UBA1*}$ or UBA1$^{GH\text{-}UBA6*}$ as the E1 enzyme and UbcH5c as the E2 enzyme. Time points: 0, 2, 5, 10, and 30 min. **h**, Quantification of UbcH5c~Ub formation in (g) for UBA6^{WT}, UBA1^{WT}, UBA6$^{GH\text{-}UBA1*}$ or UBA1$^{GH\text{-}UBA6*}$. **i**, Quantification of BIRC6^{UBC} ~Ub formation for UBA6^{WT}, UBA1^{WT}, and UBA1$^{GH\text{-}UBA6*}$ from the transthioesterification assays shown in **Fig. 4h**. **j**,

Quantification of $BIRC6^{UBC}$~Ub and $BIRC6^{UBC\Delta insertion\ loop}$~Ub formation for $UBA6^{WT}$, $UBA1^{WT}$, from the assays shown in **Fig. 4i**. Data are presented as mean ± s.d. from two independent biological experiments. In these biochemical assays, fluorescently labelled ubiquitin was used, and both fluorescent signals and the corresponding Coomassie Brilliant Blue-stained gels are shown; quantification was based on the Coomassie Brilliant Blue-stained gels and calculated as the percentage of Ub-charged E2 enzyme relative to total E2 enzyme.

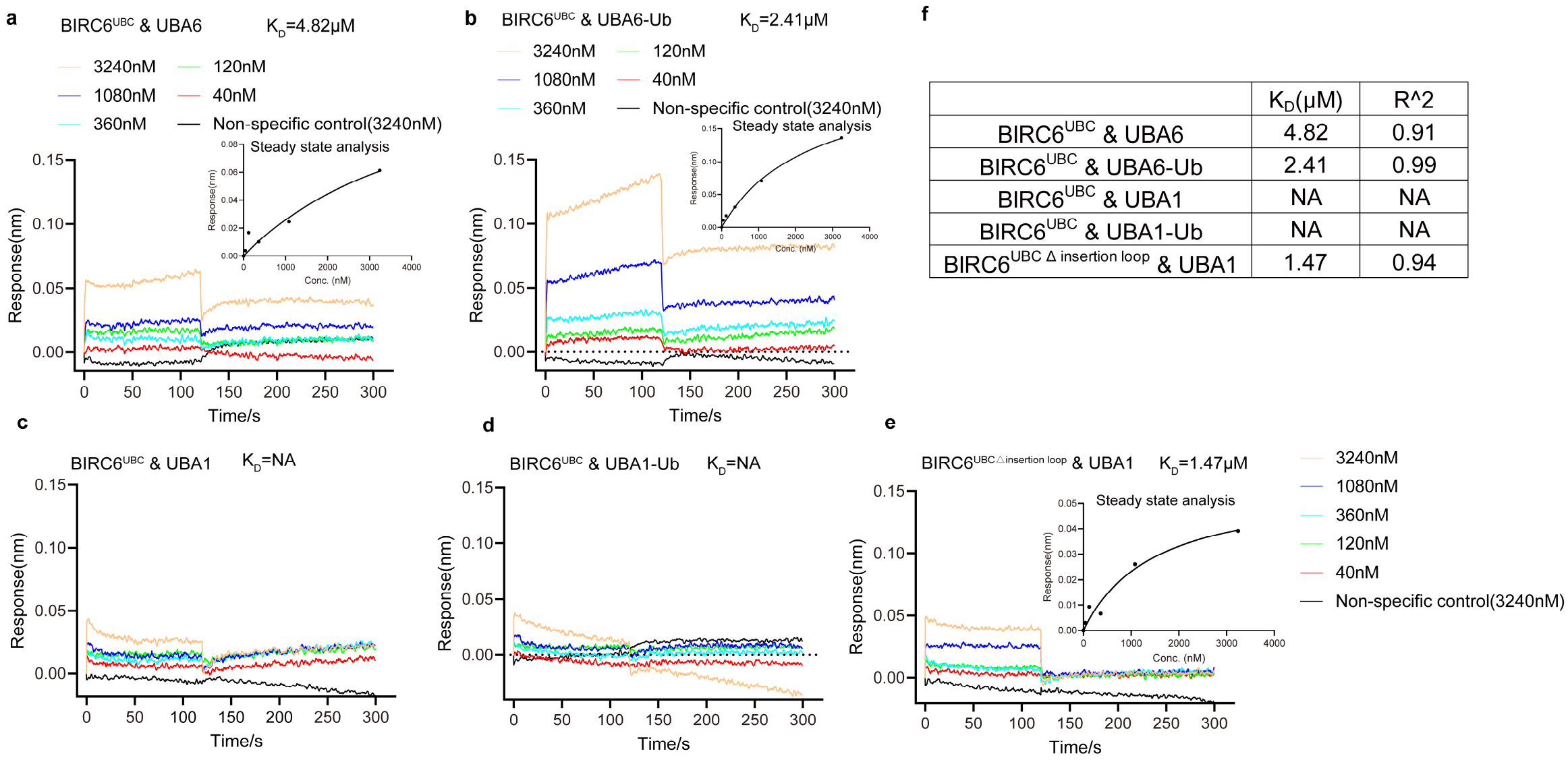

	K_D(μM)	R^2
BIRC6^UBC & UBA6	4.82	0.91
BIRC6^UBC & UBA6-Ub	2.41	0.99
BIRC6^UBC & UBA1	NA	NA
BIRC6^UBC & UBA1-Ub	NA	NA
BIRC6^UBC Δ insertion loop & UBA1	1.47	0.94

Supplementary Fig. 9 | BLI binding measurements of BIRC6^{UBC} and variants to E1 and Ub-loaded E1 using His1K biosensor. a-e, BLI analysis of binding of BIRC6^{UBC} to UBA6 (**a**), UBA6-Ub (**b**), or UBA1 (**c**), UBA1-Ub (**d**) and of BIRC6$^{UBC\Delta insertion\ loop}$ to UBA1 (**e**). Concentrations of BIRC6^{UBC} and BIRC6$^{UBC\Delta insertion\ loop}$ used were 40, 120, 360, 1080, and 3240 nM. Right panels in (a), (c), and (e) show binding curves fitted by steady-state analysis. NA = Not available; binding curve could not be obtained by steady-state analysis within the tested concentration range. **f**. Summary of K_D values derived from BLI assays.

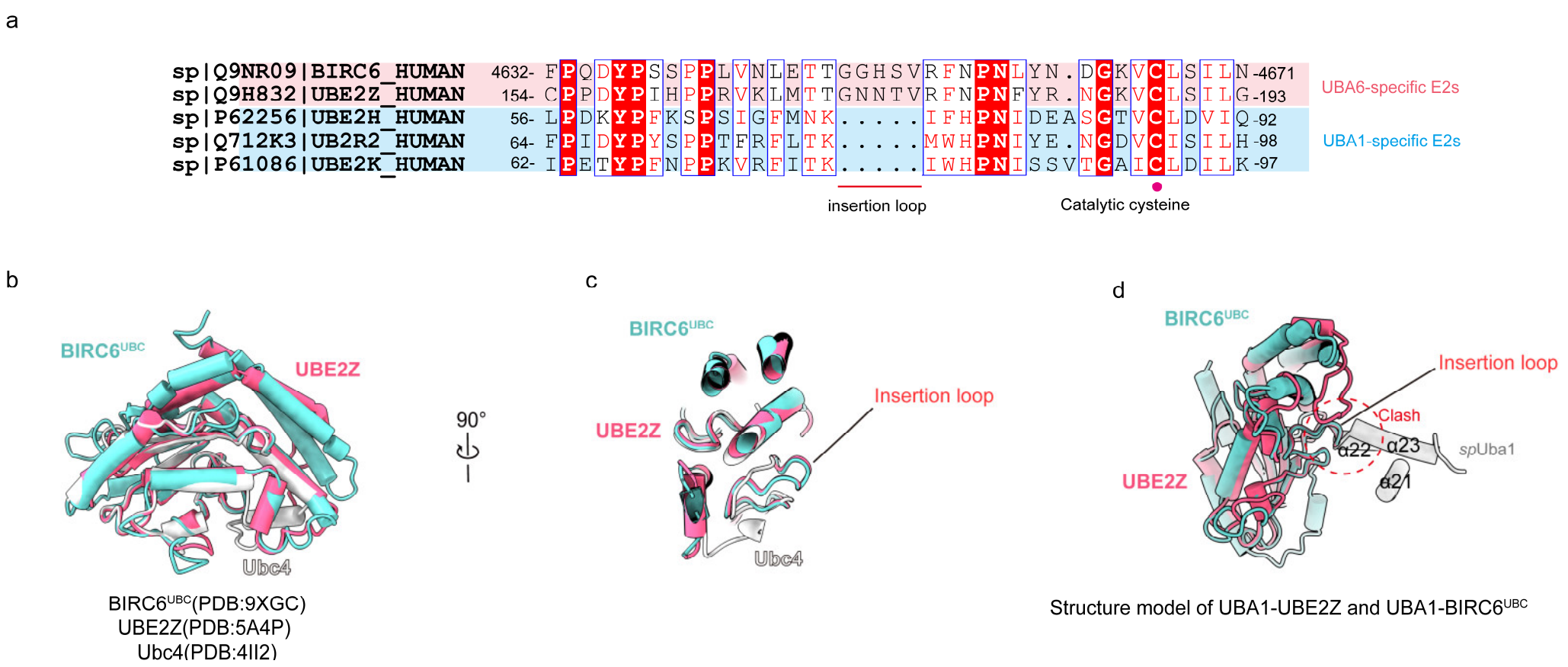

Supplementary Fig. 10 | Structural and sequence alignments of UBE2Z and BIRC6^{UBC}. a, Sequence alignment of UBA6-specific and UBA1-specific E2s. **b-c**, Structural alignment of the crystal structure of UBE2Z (PDB: 5A4P), Ubc4 (PDB: 4II2) and BIRC6^{UBC} determined in this study. **d**, Steric clash between UBA1 and UBE2Z may involve in the insertion loop of UBE2Z and the α22-α23 helix of UBA1.

2. Supplementary table 1: Oligonucleotides used for Cloning

Description	Sequence	Purpose
UBA6_C625A_f	cgtttgccaccctgaagtcctttccggc	Quikchange mutagenesis
UBA6_C625A_r	cagggtggcaaacgggatctcctcctccg	Quikchange mutagenesis
UBA6_Gate Helix(UBA1)_gene-f	caataaattctggcaagaccccaagtttgtggagcg	Homologous recombination
UBA6_Gate Helix(UBA1)_gene-r	gagaccaattacgcggtcgctgcagcaccaggc	Homologous recombination
UBA6_Gate Helix(UBA1)_Vector_f	cacaaacttggggtcttgccagaatttattgaacaaggacggc	Homologous recombination
UBA6_Gate Helix(UBA1)_Vector_r	ctggtgctgcagcgaccgcgtaattggtctcaatgtgttg	Homologous recombination
UBA6_C625S_f	cgttttccaccctgaagtcctttccggc	Quikchange mutagenesis
UBA6_C625S_r	cagggtggaaaacgggatctcctcctccg	Quikchange mutagenesis
UBA1_C623S_f	ccatctccaccctgaagaacttccctaatgcc	Quikchange mutagenesis
UBA1_C623S_r	gggtggagatggggatggacttctcaggtg	Quikchange mutagenesis
UBA1_Gate Helix(UBA6)_gene-f	caaccagtacctcacaacctatagcagcgccgagg	Homologous recombination
UBA1_Gate Helix(UBA6)_gene-r	cagcccaggtctgtgggcgacgagacaacagtttgatcac	Homologous recombination
UBA1_Gate Helix(UBA6)_Vector-f	ggcgctgctataggttgtgaggtactggttgacattttctgc	Homologous recombination
UBA1_Gate Helix(UBA6)_Vector-r	ctgttgtctcgtcgcccacagacctgggctgactg	Homologous recombination
UBC_del insertion loop-f	ggaaactacccgcttcaaccccaacctgtataacg	Quikchange mutagenesis
UBC_del insertion loop-r	gttgaagcgggtagtttccaaattgacaaggggcg	Quikchange mutagenesis
UBC_C147A_f	ggcaaagttgcactctcaattctgaatacatggcatggaagac	Quikchange mutagenesis
UBC_C147A_r	gagagtgcaactttgccgtcgttatacaggttg	Quikchange mutagenesis
UBC_N-extension-f	ctaacgatagaaatagtgcagctagggctcggc	Quikchange

		mutagenesis
UBC_N-extension-r	ctgcactatttctatcgttagcgtttttgacctggctc	Quikchange mutagenesis
UBC_β4 loop_f	cttcccagctgattacccttccagcccgcc	Quikchange mutagenesis
UBC_β4 loop_r	gtaatcagctgggaagtaaacgtcaaactcgaagcatc	Quikchange mutagenesis
UBC_E4604R_f	gatgtgatgaaagaagattggatattatgaaagttctgatcaccggc	Quikchange mutagenesis
UBC_E4604R_r	cataatatccaatcttctttcatcacatctcacgaagactgaactactg	Quikchange mutagenesis
UBC_R4605G_f	gaagagggcttggatattatgaaagttctgatcaccggccc	Quikchange mutagenesis
UBC_R4605G_r	cataatatccaagccctcttcatcacatctcacgaagactgaactactg	Quikchange mutagenesis
UBC_L4606G_f	gaagagagaggcgatattatgaaagttctgatcaccggcccc	Quikchange mutagenesis
UBC_L4606G_r	cataatatcgcctctctcttcatcacatctcacgaagactgaactac	Quikchange mutagenesis
UBC_P4709R_f	ctttaacgagaggggttacgagaggtctcgaggcac	Quikchange mutagenesis
UBC_P4709R_r	cgtaacccctctcgttaaagtaaggttccgccacg	Quikchange mutagenesis
UBC_Y4711G_f	cctggtggcgagaggtctcgaggcacaccatc	Quikchange mutagenesis
UBC_Y4711G_r	cctctcgccaccaggctcgttaaagtaaggttccg	Quikchange mutagenesis
UBC_R4745A_f	gaacagattgctaatccctctccttgcttcaaggagg	Quikchange mutagenesis
UBC_R4745A_r	gagggattagcaatctgttcgagcatagcccacttc	Quikchange mutagenesis
