# Supplementary material for: Steric Gating Contributes to the Preferential Ubiquitin Charging of BIRC6 by UBA6 versus UBA1
Source: Nat Commun. 2026 Aug 29;17:10349. doi: 10.1038/s41467-026-75898-6 (PMC13623948; doi:10.1038/s41467-026-75898-6)
Supplement: Supplementary file 2 [file 41467_2026_75898_MOESM2_ESM.pdf]

nature portfolio

Corresponding author(s): Huasong Ai, Lei Liu

Last updated by author(s): Jul 3, 2026

Reporting Summary

Nature Portfolio wishes to improve the reproducibility of the work that we publish. This form provides structure for consistency and transparency in reporting. For further information on Nature Portfolio policies, see our Editorial Policies and the Editorial Policy Checklist.

Statistics

For all statistical analyses, confirm that the following items are present in the figure legend, table legend, main text, or Methods section.

n/a	Confirmed	
☐	☒	The exact sample size (*n*) for each experimental group/condition, given as a discrete number and unit of measurement
☐	☒	A statement on whether measurements were taken from distinct samples or whether the same sample was measured repeatedly
☐	☒	The statistical test(s) used AND whether they are one- or two-sided<br>*Only common tests should be described solely by name; describe more complex techniques in the Methods section.*
☒	☐	A description of all covariates tested
☒	☐	A description of any assumptions or corrections, such as tests of normality and adjustment for multiple comparisons
☐	☒	A full description of the statistical parameters including central tendency (e.g. means) or other basic estimates (e.g. regression coefficient) AND variation (e.g. standard deviation) or associated estimates of uncertainty (e.g. confidence intervals)
☐	☒	For null hypothesis testing, the test statistic (e.g. *F*, *t*, *r*) with confidence intervals, effect sizes, degrees of freedom and *P* value noted<br>*Give P values as exact values whenever suitable.*
☒	☐	For Bayesian analysis, information on the choice of priors and Markov chain Monte Carlo settings
☒	☐	For hierarchical and complex designs, identification of the appropriate level for tests and full reporting of outcomes
☒	☐	Estimates of effect sizes (e.g. Cohen's *d*, Pearson's *r*), indicating how they were calculated

Our web collection on statistics for biologists contains articles on many of the points above.

Software and code

Policy information about availability of computer code

Data collection	Cryo-EM data are collected on a 300kV Titan Krios microscope with a Falcon4;<br>Gel Imaging: Image Lab Touch Software-2.4.0.03; Octet® R8 system (Sartorius); Chirascan Plus spectrometer (Applied Photophysics, Leatherhead, Surrey, United Kingdom)
Data analysis	Cryo-EM: Motion Cor2(1.2.6), RELION-3.1.1, KaiZhang's gctf(v1.18).<br>Model Building: PHENIX(1.19.2), COOT(0.8.2).<br>Molecular Visualization: ChimeraX(1.7.1).<br>GraphPad Prism (9.5); Image Lab-6.0.1; Octet BLI Analysis 12. Software (Sartorius); Origin(2024)

For manuscripts utilizing custom algorithms or software that are central to the research but not yet described in published literature, software must be made available to editors and reviewers. We strongly encourage code deposition in a community repository (e.g. GitHub). See the Nature Portfolio guidelines for submitting code & software for further information.

Data

Policy information about availability of data

All manuscripts must include a data availability statement. This statement should provide the following information, where applicable:

- Accession codes, unique identifiers, or web links for publicly available datasets
- A description of any restrictions on data availability
- For clinical datasets or third party data, please ensure that the statement adheres to our policy

The cryo-EM maps and atomic model of singlely-loaded UBA6–BIRC6UBC–UbT complex and doublely-loaded UBA6–BIRC6UBC–UbT–UbA complex have been deposited in the Electron Microscopy Data Bank (EMDB: www.ebi.ac.uk/pdbe/emdb/) and Protein Data Bank (PDB:www.rcsb.org) under accession codes: EMD-66842 and PDB 9XGB (singlely-loaded UBA6–BIRC6UBC–UbT complex), EMD- 66843 and PDB 9XGC (doublely-loaded UBA6–BIRC6UBC–UbT–UbA complex), respectively.

Research involving human participants, their data, or biological material

Policy information about studies with human participants or human data. See also policy information about sex, gender (identity/presentation), and sexual orientation and race, ethnicity and racism.

Reporting on sex and gender	This part is not included in this study.
Reporting on race, ethnicity, or other socially relevant groupings	This part is not included in this study.
Population characteristics	This part is not included in this study.
Recruitment	This part is not included in this study.
Ethics oversight	This part is not included in this study.

Note that full information on the approval of the study protocol must also be provided in the manuscript.

Field-specific reporting

Please select the one below that is the best fit for your research. If you are not sure, read the appropriate sections before making your selection.

☒ Life sciences ☐ Behavioural & social sciences ☐ Ecological, evolutionary & environmental sciences

For a reference copy of the document with all sections, see nature.com/documents/nr-reporting-summary-flat.pdf

Life sciences study design

All studies must disclose on these points even when the disclosure is negative.

Sample size	No statistical methods were used to predetermine the sample size. Biochemical experiments producing qualitative results were performed independently twice or more to ensure reproducibility. For cryo-EM,the sample sizes were determined by available electron microscopy measuring time and density of the particles on the sample grids, the representative micrograph was shown, with other micrographs exhibiting similar particle density and dispersion.
Data exclusions	No data were excluded from the analysis.
Replication	The in vitro transthioesterification assays were performed in three independent biological replicates. Bio�layer interferometry (BLI) and circular dichroism (CD) were not repeat as both techniques have high intrinsic reproducibility. Structure determination does not require replication, the 3D reconstitution of particles at different angles gives consistent results.<br>All replication attempts were successful.
Randomization	Randomization was not relevant to this study because no grouped samples were involved.
Blinding	Blinding was not relevant to this study because no grouped samples were involved.

Reporting for specific materials, systems and methods

We require information from authors about some types of materials, experimental systems and methods used in many studies. Here, indicate whether each material, system or method listed is relevant to your study. If you are not sure if a list item applies to your research, read the appropriate section before selecting a response.

Materials & experimental systems

n/a	Involved in the study
☒	☐ Antibodies
☐	☒ Eukaryotic cell lines
☒	☐ Palaeontology and archaeology
☒	☐ Animals and other organisms
☒	☐ Clinical data
☒	☐ Dual use research of concern
☒	☐ Plants

Methods

n/a	Involved in the study
☒	☐ ChIP-seq
☒	☐ Flow cytometry
☒	☐ MRI-based neuroimaging

Eukaryotic cell lines

Policy information about cell lines and Sex and Gender in Research

Cell line source(s)	HEK293F(Expi293F)cells from Thermo Fisher Scientific (Cat#A14528).
Authentication	None of the cell lines used were authenticated.
Mycoplasma contamination	Confirm that all cell lines tested negative for mycoplasma contamination.
Commonly misidentified lines (See ICLAC register)	No commonly misidentified cell lines were used.

Plants

Seed stocks	This part is not included in this study.
Novel plant genotypes	This part is not included in this study.
Authentication	This part is not included in this study.
