# Supplementary material for: Steric Gating Contributes to the Preferential Ubiquitin Charging of BIRC6 by UBA6 versus UBA1
Source: Nat Commun. 2026 Aug 29;17:10349. doi: 10.1038/s41467-026-75898-6 (PMC13623948; doi:10.1038/s41467-026-75898-6)
Supplement: Supplementary file 3 [file 41467_2026_75898_MOESM3_ESM.pdf]

nature portfolio

Peer Review File

Steric Gating Contributes to the Preferential Ubiquitin Charging of BIRC6 by UBA6 versus UBA1

Corresponding Author: Professor Huasong Ai

This file contains all reviewer reports in order by version, followed by all author rebuttals in order by version.

Version 0:

Reviewer comments:

Reviewer #1

(Remarks to the Author)
In this manuscript by Tong et al., the authors explore the structural mechanism that underlies the specificity of BIRC6 for the E1 enzyme UBA6 not UBA1. They solve the cryo EM structure of the UBC domain of BIRC6 in complex with UBA6 that is singly or doubly charged with ubiquitin. Together with binding activity assays they show that UBA6 contains structural features that differ from UBA1 make UBA6 suitable for interaction with BIRC6 for efficient charging. This work significantly advances our understanding of E1 E2 selectivity justifies publication in Nature Communications after the points below are addressed.
recent paper on UBA6 specificity for E2 enzymes has appeared (PMID 41350950). The authors should discuss their findings in the light of this study. While the new paper may reduce the novelty of the present work to some extent, the current manuscript still provides important novel insights that justify publication. In the Discussion, please elaborate on the differences between the binding affinities of UBA6 BIRC6 reported in your manuscript those reported in the study with PMID 41350950. This is major difference between the papers. It has direct impact on the mechanistic interpretation therefore needs to be clearly addressed.
The authors suggest that the insertion loop in the BIRC6 UBC domain clashes with the gate loop of UBA1. Consistent with this, introducing the UBA6 gate loop into UBA1 restores activity (Fig. 4J), UBA1 can charge the BIRC6 UBC domain lacking the insertion loop (Fig. 4H). If this is the reason that UBA1 does not function with BIRC6, the authors should also test whether UBA6 containing the UBA1 gate loop can work with the BIRC6 UBC domain lacking the insertion loop. This experiment would strengthen their model that specificity is determined by the combined effects of the gate loop the insertion loop.
In Figure 3I, the authors suggest that the mutations reduce charging because they weaken the interactions between the proteins. However, one cannot rule out the possibility that some mutations disrupt the overall structure of the protein, which could also reduce charging. Please provide insight into whether the mutant proteins remain properly folded.
In Figure 5A, the authors test the binding of BIRC6 to UBA1 or UBA6 suggest that BIRC6 has similar affinity for both. They further propose that the reason BIRC6 is not functional with UBA1 is not lack of binding but restricted movement of the UFD, which blocks charging. If that is the case, then UBA1~Ub should also be able to bind BIRC6. Please repeat the binding experiments shown in Figure 5A using UBA1~Ub UBA6~Ub. This will clarify whether the charged forms of the E1s display similar binding affinities to BIRC6.
In the in vitro transthioesterification assays using fluorescently labeled Ub, please show the full gel so that charging of the E1 is also visible. This is especially important in the gels where BIRC6 charging does not occur, since confirming that the E1 is charged is essential for interpreting these results.
In Figure 5B, the authors suggest that the decreased charging of BIRC6 with Ub by UBA6 in the presence of UBA1 is due to binding of UBA1 to BIRC6, thereby sequestering BIRC6 from UBA6. However, based on Figure 5B, one cannot exclude the possibility that inhibition arises because UBA1 becomes charged with Ub, which reduces the pool of free Ub. To address this, the authors should repeat the experiment using catalytically inactive UBA1.

Reviewer #2

(Remarks to the Author)
The paper by Tong colleagues reports the structure of the non-canonical ubiquitin-activating E1 enzyme, UBA6, bound to the E2 BIRC6. Mammals encode two genes for ubiquitin activating E1 enzymes: UBA1 UBA6 while UBA1 is seen as the

most relevant E1 for performing most cellular ubiquitination events the roles of UBA6 are not well understood.

By generating ubiquitin dehydroalanine probe, the authors cleverly trap ubiquitin, UBA6, BIRC6 as ternary complex use these structures to understand why BIRC6 can only work with UBA6, not UBA1.

study into ubiquitin E1-E2 specificity was recently published in NSMB (PMID: 41350950) that captured structures of BIRC6 bound to UBA6 ubiquitin through an identical strategy. Importantly, that study reports different modes of BIRC6 interaction specificity with UBA6. Some of the conclusions from this manuscript do not agree with the other published study.

This manuscript under review lacks rigorous controls suffers from several instances of data overinterpretation. There is also lack of transparency of different assay conditions used which would alter the conclusions reached. There is no source data of uncropped gels or coomassie-stained gels for validation of loading the reader is left to qualitatively interpret fluorescent loading, sometimes between different gels. There are also numerous mistakes in the writing presentation of the manuscript (for example multiple occasions of BRIC6 instead of BIRC6).

In its present form, I cannot recommend this manuscript for publication would expect to see major revisions to address the discrepancies in binding between UBA1 UBA6 the proposed mutations that are believed to alter E1 specificity of BIRC6.

Specific major points are listed below:

1. For the two states obtained (single or doubly loaded UBA6-BIRC6), it would be helpful for the authors to compare if there were any differences in the position of BIRC6 in the two states. In addition, it was not clear whether ATP was observed in the adenylation domain for the singly-loaded BIRC6-UBA6. Given recent insights from the Lima lab showed coupling of ubiquitin transfer to the adenylation domain (PMID: 39143218) the authors should investigate any differences between the two states.

2. No maps/models have been provided by the authors to confirm quality. It appears from Figure 2d that the E1 Cys is in the wrong configuration to be forming thioester bond to ubiquitin. The authors need to correct this demonstrate that they can clearly model the relevant bonding distances angles in their EM maps. It thus raises whether the rest of the maps have been correctly interpreted.

3. How was fluorescence quantified in Figure 3h? In all instances throughout the paper, there are no uncropped gels showing the other components present or relevant Coomassie-stained gels confirming equal loading.

4. The authors describe several interfaces between BIRC6 UBA6, notably within the UBA6 UFD domain, make mutations within BIRC6 to test the contribution of each of the interfaces. Some of the mutations are conservative loss of functional groups whereas others are charge-swap in some instances destabilising (introduction of glycine). Can the authors provide rationale for the choice of mutations made? Also, very few are single amino acid substitutions but rather mutations of several residues at time - which residues within the b1-b2 loop or a3 insertion particularly affect the BIRC6-UBA6 interaction?

5. The authors describe “gate helix” within the SCCH domain of UBA1 that is absent in UBA6 as providing steric clash preventing BIRC6 function. They then make deletion of the equivalent region in BIRC6 measure transthiolation activity observe BIRC6 functioning with UBA1 at levels comparable to UBA6. Transfer from UBA1 onto mutant BIRC6 should be quantified compared to transfer from UBA6 onto WT/mutant BIRC6.

The authors then make corresponding deletions/insertions in UBA1 UBA6 to suggest specificity mechanisms. Extended Figure 8 b, c, show that the activity of the E1s for loading ubiquitin is reduced with both UBA1 UBA6 mutants. Extreme caution should therefore be applied when interpreting any effects on transfer to E2s from these E1 mutants. Reference to this figure description of the different activities of these E1 mutants is omitted from the main text. The authors need to include more controls to show the mutated E1s are still functional with other E2s that do not contain the insertion loop identified in BIRC6. to determine if general E2 transfer has been altered.

An additional important point, the methods section states these specificity swap assays were performed at 37 C for 30min compared to R.T. for 2min for structural mutants. It is therefore misleading to suggest any of these mutations have altered specificity when left for 15x longer. Quantification of transfer at 2 min should be performed.

6. The authors used microscale thermophoresis to demonstrate that BIRC6 bound to UBA6 UBA1 with equal affinity. Here it will be important for the authors to validate this through orthogonal means. The other published paper has used fluorescence polarisation to detect the interaction between BIRC6 UBA6, reporting nanomolar interaction between the two, no interaction between BIRC6 UBA1. Can the authors reconcile their findings? The authors should consider switching the label onto the smaller protein of the interaction pair (i.e. BIRC6) see if they observe similar MST response. In addition, they should consider testing the binding of another cross-reactive E2 to UBA1 UBA6.

Also, is the binding of BIRC6 to either UBA1 or UBA6 altered by the presence of ubiquitin loaded onto the E1? Given the author's current MST setup, this will be fairly easy to test may reveal interesting observations.

7. The authors attempt to further demonstrate binding of UBA1 to BIRC6 by means of competition assay where UBA1 is titrated into UBA6-BIRC6 reaction. Figure 7b is confusing in that there is no ubiquitin loaded on UBA6 in the absence of

UBA1, which is strange. Can the authors clarify this?

The authors should determine if UBA1 is outcompeting UBA6 loading of BIRC6 due to the consumption of ATP in the assay caused by increasing UBA1 concentrations. Does an inactive UBA1 (either adenylation incompetent, or thiolation inactive) also out-compete UBA6-dependent transfer onto BIRC6? Furthermore, if BIRC6 is binding to UBA1 in catalytic incompetent state as suggested, an effect on UBA1-functioning E2s would be observed when BIRC6 is titrated into UBA1 reactions containing other E2s. This should be demonstrated by the authors.

8. Finally, the authors generate model whereby the UFD domain from their BIRC6 cryoEM structure is docked onto the UFD domain of PDB ID 4NNJ to reveal conformational difference of UFD-BIRC6 that prevents BIRC6 full engagement with UBA1. I find the rationale for this confusing since in Extended Figure 8 there is little RMSD difference between the author's structure reported E1-E2 structures, which are mostly UBA1-derived. What is the overall RMSD between the entire E1 of 4NNJ the reported UBA6-BIRC6 structure? It is known that the UFD domain of E1s can adopt different conformations, so the rationale for taking an E1 not bound to an E2 superimposing just the UFD domains seems flawed.

Other points

Line 15. BIRC6 is not "specifically activated" by UBA6 as written by the authors. E2s are not activated by E1s. There are several instances throughout the text where this is referred to.

Lines 59 62. It is not clear what the apoptotic pathway being referred to is. In addition, reference 31 describes the role of BIRC6 in regulating autophagy, not programmed cell death.

Line 95. This sentence requires citation of work demonstrating double-loaded E1 is required for maximum ubiquitin transfer onto an E2.

Line 102. It is unclear what the authors mean by "the atomic-level precision of protein chemical synthesis" the associated citations 36-41 are irrelevant to this manuscript.

Line 142. The authors should acknowledge the Lima lab first used focused 3D classification for resolving ubiquitin transfer states observed when using the PSAN probe approach for capturing ubiquitin transfer from UBA1 to Ubc4 (PMID: 39143218).

Line 150. Lacking reference for the activity of double-loaded ubiquitin E1.

Line 218. In the interests of transparency, the authors should highlight that the gate helix E1 chimeras have lower thiolation activity (Extended Figure 8). In addition, the authors should demonstrate that transfer of ubiquitin on UBA1/6 dual active E2s is not impaired.

The methods section outlines differences in reaction conditions used for the transthiolation assays. This should be clearly stated in figure legends e.g. Figures 1, & need to highlight the different lengths of time the assays were performed for. i.e. structural activities probed after 2minutes, whereas specificity switch mutations were visualised after 30-minute reaction.

Version 1:

Reviewer comments:

Reviewer #1

(Remarks to the Author)
The authors addressed all my concerns, I support publication in Nature Communications.

Reviewer #2

(Remarks to the Author)
The authors have made good attempt to respond to both reviewers' comments. However, I continue to have concerns about the interpretation of the affinity measurements that the authors use to underpin their conclusions.

1. The authors have now included second technique (BLI) to measure E1-BIRC6 affinity that gives very different affinities. While I appreciate that differences in affinities can arise between orthogonal techniques, I disagree with the authors' suggestion that 3-10 fold difference in affinities is still acceptable. Notably in their revised BLI data the difference for BIRC6 binding to UBA6 is almost 17-fold different between MST BLI (23 µM versus 1.3 µM). Equally concerning is the reciprocal experiment performed by the authors in Rev. Fig 12c,d (incidentally which is not included in the manuscript but only in the rebuttal) where the authors claim to observe Kd of 5.6 µM when the label is on BIRC6 UBC. It is not clear how the authors could determine Kd from this fit since at the concentration range used the fraction bound does not reach 1 the quality of the data points is noisy.

2. The authors now record an affinity between BIRC6 Ub-loaded E1. Once again there is discrepancy between the two

techniques. The increase in affinity for BIRC6 to the UBA6-Ub loaded state observed by MST (15 fold increase) is interesting. However, by BLI, the increase observed is less significant (approximately 1.6 fold). The authors conclude that this makes UBA6 the preferred E1. Given the authors stated that 3-10 fold difference in affinities is acceptable for concluding comparable affinity, how can the authors conclude that 1.6 fold difference by BLI is significant? Furthermore, can the authors explain mechanistically how Ub-loaded UBA6 would bind better to UBA6 than Ub-loaded UBA1?

3. Related to 1) 2) I am not sure the authors should be so confident on the significance of specific interactions given the large differences between affinity measurements between MST BLI? The authors note 1.5-fold difference in binding of BIRC6 UBC lacking the insertion loop to UBA1, compared to wild-type BIRC6, but this effect is very modest compared to the difference of BIRC6 binding to UBA6-Ub, hence it is likely not affinity-driven differences. more relevant interpretation would be the SCCH domain positions BIRC6 into correct orientation for transthioesterification to occur, the clash between the BIRC6 insertion loop the UBA1 SCCH prevents proper orientation.

4. The authors argue that the differences in binding between UBA1 UBA6 of BIRC6 by Riechmann et al is due to the different techniques used. I agree with this point but still do not understand how complex of ~1µM Kd with similar kinetic properties with regards to on off rates as observed in BLI (Figure 5c,d) can still not bind in analytical SEC observed by Riechmann et al. The authors should not state that this complements the Riechmann et al. study because there is still discrepancy between the two.

5. The molecular basis for specificity between BIRC6 UBA6 is described as the complementary interactions between the gate helix of UBA6 an insertion loop within BIRC6. The authors now show improved biochemistry in order to validate this model but it should be noted that the effects are moderate as while removing the insertion loop from BIRC6 has the strongest effect towards UBA1 activity, albeit with reduced kinetic effects (Figure 4i, Extended Figure 8j), the reciprocal experiment of replacing the gate helix in UBA1 with that of UBA6 only restored activity to 25% with BIRC6 (Figure 4h, Extended Figure 8i). Given that other papers (Riechmann et al Nayak et al) have shown the contribution of both the SCCH UFD for driving BIRC6 UBE2Z specificity, is it likely that including this mutation in UBA6-swapped UFD domain would further enhance activity towards BIRC6?

6. Both reviewers asked about the competition assay, the authors present additional data in the rebuttal with UBA1 mutants that cannot bind ATP, or cannot undergo thiolation. However, the data has been removed from the manuscript. Is this the intention? The data in Rev. Figure (and 15) is anyway confusing as the quantification of the assay in Rev. Figure 4b actually shows that UBA1 is only slightly inhibiting UBA6-BIRC6 loading is partially dependent on UBA1's ability to adenylate Ub, i.e. it is competing for ATP in the reaction mixture. This appears to contrast the original data presented by the authors (Rev. Figure 14).
Furthermore, the authors state UBA1 UBA6 bind to BIRC6 with comparable affinities therefore why is 60-fold excess of UBA1 to UBA6 required to observe 20% inhibition, this would not make sense if both UBA1 UBA6 have equivalent binding affinities to BIRC6?

Version 2:

Reviewer comments:

Reviewer #2

(Remarks to the Author)
I thank the authors for having the courage to revisit some of their earlier experiments on BLI MST to identify non-specific interactions that were occurring. Based on these observations, the authors have revised their narrative have removed some of the BLI all the MST data. Their overall model of steric gating is still supported by biochemical validation provides additional insights that complement two recent papers describing the specificity of UBA6. Overall I have no further comments recommend publication of the manuscript.

Open Access This Peer Review File is licensed under a Creative Commons Attribution 4.0 International License, which permits use, sharing, adaptation, distribution and reproduction in any medium or format, as long as you give appropriate credit to the original author(s) and the source, provide a link to the Creative Commons license, and indicate if changes were made.
In cases where reviewers are anonymous, credit should be given to 'Anonymous Referee' and the source.
The images or other third party material in this Peer Review File are included in the article's Creative Commons license, unless indicated otherwise in a credit line to the material. If material is not included in the article's Creative Commons license and your intended use is not permitted by statutory regulation or exceeds the permitted use, you will need to obtain permission directly from the copyright holder.
To view a copy of this license, visit https://creativecommons.org/licenses/by/4.0/

Reviewer #1:

In this manuscript by Tong et al., the authors explore the structural mechanism that underlies the specificity of BIRC6 for the E1 enzyme UBA6 and not UBA1. They solve the cryo EM structure of the UBC domain of BIRC6 in complex with UBA6 that is singly or doubly charged with ubiquitin. Together with binding and activity assays they show that UBA6 contains structural features that differ from UBA1 and make UBA6 suitable for interaction with BIRC6 and for efficient charging. This work significantly advances our understanding of E1 and E2 selectivity and justifies publication in Nature Communications after the points below are addressed.

Response: We thank the reviewer for the positive comments.

A recent paper on UBA6 specificity for E2 enzymes has appeared (PMID 41350950). The authors should discuss their findings in the light of this study. While the new paper may reduce the novelty of the present work to some extent, the current manuscript still provides important and novel insights that justify publication. In the Discussion, please elaborate on the differences between the binding affinities of UBA6 and BIRC6 reported in your manuscript and those reported in the study with PMID 41350950. This is a major difference between the papers. It has a direct impact on the mechanistic interpretation and therefore needs to be clearly addressed.

Response: We thank the reviewer for acknowledging the value of our work. In the recent studies by Reichmann et al. (*Nat. Struct. Mol. Biol.* 2026, 33, 464–478; PMID 41350950) and Nayak et al. (*Nat. Commun.* 2026, PMID 41764162), domain swap experiments between UBA1 and UBA6 demonstrated that both the UFD and SCCH domains are critical for the specificity of BIRC6 toward UBA6. Through structural and biochemical analyses, Riechmann et al. further elucidated the molecular mechanism by which the UFD domain of UBA6 contributes to this specificity. Our study complements these findings by revealing the role of the SCCH domain. The gate helix within the UBA6 SCCH domain adopts a distinct conformation structurally configured to accommodate an insertion loop of BIRC6^{UBC}. In contrast, the corresponding helix in UBA1 extends inward toward the E2, causing steric clashes with the insertion loop of BIRC6. **Together, these findings are not in conflict with those reported by Riechmann et al. but rather complement them.** Both the UFD and SCCH domains are critical for UBA6–BIRC6 specificity, with the UFD governing partner selection and the SCCH optimizing catalytic geometry. Together with the work by Riechmann et al., our studies provide a more complete mechanistic understanding of UBA6-specific BIRC6 recognition.

Regarding the differences in binding affinities between our work and that of Riechmann et al. Riechmann et al. reported that BIRC6^{UBC} co-eluted with UBA6 but not with UBA1 in size-exclusion chromatography (SEC). Using fluorescence polarization (FP), they further determined the binding affinity between BIRC6^{UBC} and UBA6 to be approximately 200 ± 58 nM; binding to UBA1 was not assessed. In contrast, our MicroScale Thermophoresis (MST) experiments yielded K_D values of 23 ± 4 μM and 13 ± 4 μM for BIRC6^{UBC} binding to UBA6 and UBA1, respectively. Additionally, our newly performed Bio-Layer Interferometry (BLI) measurements gave K_D values of 1.32 ± 0.02 μM and 1.27 ± 0.02 μM, respectively (**Rev. Fig. 1a, b, c d, i**).

1. Differences in affinity values obtained by FP, MST, and BLI

Different binding affinity techniques often yield distinct numerical values due to differences in

their underlying principles. Factors such as buffer composition, temperature, and whether the interaction occurs in free solution or on a surface also contribute to these variations. Typically, differences within a 3- to 10-fold range are considered acceptable. Here, we employed two orthogonal methods (MST and BLI) to assess binding affinity. We therefore consider our results to be robust and meaningful.

2. Why SEC showed no co-elution despite comparable binding affinities

SEC and direct affinity measurements probe different aspects of protein interactions. SEC reflects complex stability and hydrodynamic behavior, capturing how long the complex persists and its overall shape. In contrast, MST and BLI measure equilibrium binding affinity, representing how tightly the proteins interact at the initial binding step. Thus, while our MST and BLI data indicate similar apparent affinities of BIRC6^{UBC} for both UBA1 and UBA6, these measurements capture the binding energy at the initial interaction. SEC reports on complex stability during prolonged separation and may be influenced by conformational states that affect complex persistence. The two sets of findings are not contradictory but rather provide complementary perspectives.

3. Effect of ubiquitin-loaded E1 on binding

We further assessed the binding of BIRC6^{UBC} to UBA1-Ub and UBA6-Ub by both MST and BLI (**Rev. Fig. 1a, c, d, e, f, i, j**). The results showed that when using oxyester-linked UBA1-Ub and UBA6-Ub conjugates to mimic the catalytically charged E1 state (where "-" denotes the oxyester linkage), BIRC6 binding to UBA6-Ub was significantly enhanced, with an approximately 9-fold increase by MST and a 2-fold increase by BLI, whereas binding to UBA1-Ub remained unchanged. This indicates that under catalytically active conditions, BIRC6^{UBC} preferentially binds UBA6-Ub, consistent with the conclusion by Riechmann et al. that a stable interaction occurs only with its cognate E1, UBA6.

Moreover, truncation of the insertion loop in BIRC6^{UBC} (BIRC6$^{UBC\Delta insertion\ loop}$) enhanced binding to UBA1-Ub, further supporting a critical role for the BIRC6 insertion loop in mediating UBA6 specificity (**Rev. Fig. 1b, g, h, i, j**).

Based on these results, we have further refined our mechanistic model. BIRC6 preferentially binds its cognate E1, UBA6-Ub, over UBA1-Ub. The gate helix of UBA6 adopts a distinct conformation that is structurally configured to accommodate the insertion loop of BIRC6^{UBC}. This refined model has been incorporated into the revised *Discussion section*, where we also provide a detailed comparison with the findings reported by Riechmann et al. and Nayak et al.

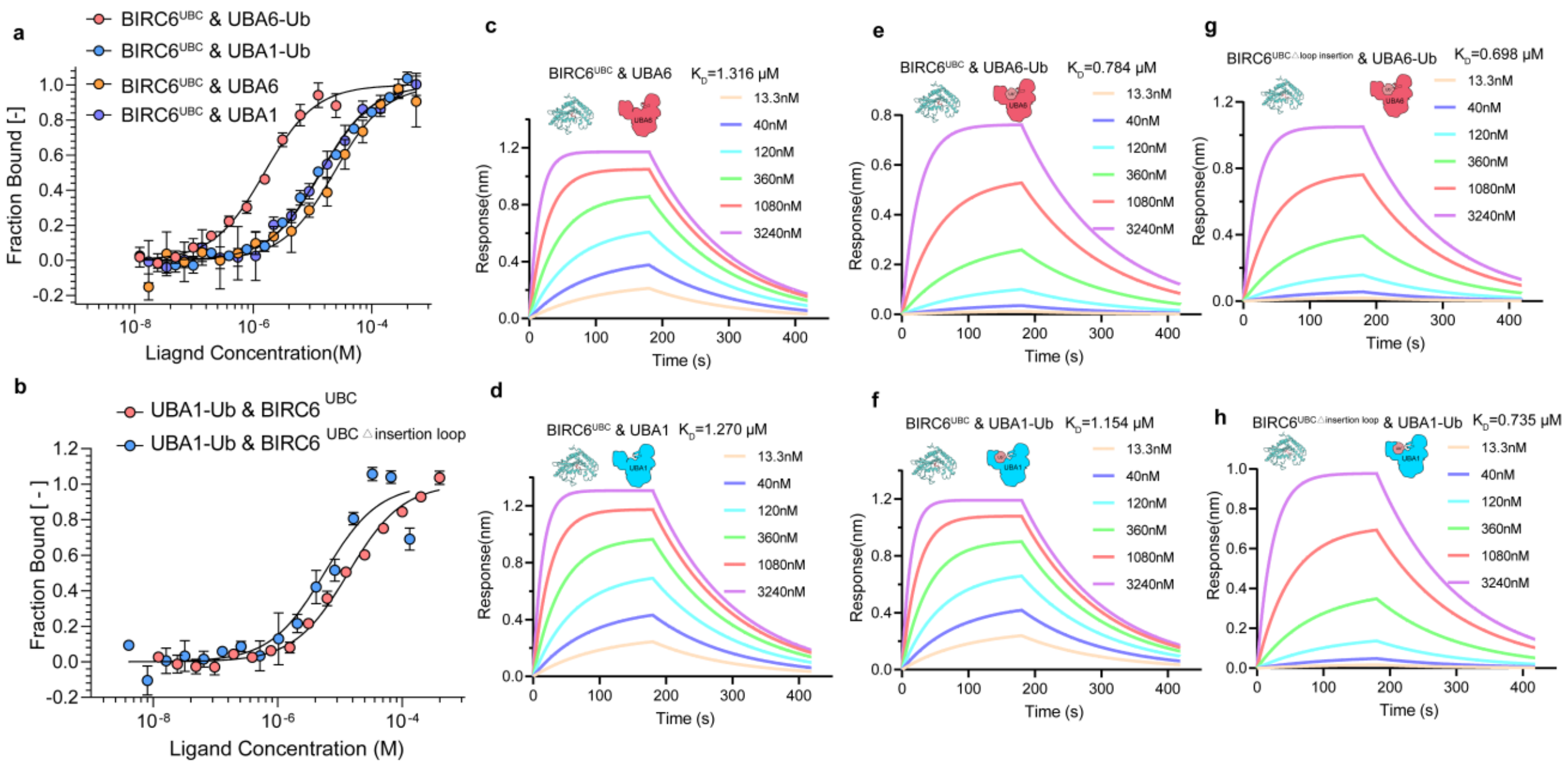

i

Summary of MST binding affinity data

Group	$BIRC6^{UBC}$	E1	K_D(μM)	K_D confidence (μM)
1	WT	UBA6	23.02	±4.29
2	WT	UBA1	14.00	±1.66
3	WT	UBA6-Ub	1.53	±0.22
4	WT	UBA1-Ub	13.58	±1.6
5	△insertion loop	UBA1-Ub	5.61	±2.11

j

Summary of BLI binding affinity data

Group	$BIRC6^{UBC}$	E1	K_D(μM)	K_D error (μM)
1	WT	UBA6	1.316	0.0218
2	WT	UBA1	1.27	0.0198
3	WT	UBA6-Ub	0.784	0.011
4	WT	UBA1-Ub	1.154	0.019
5	△insertion loop	UBA6-Ub	0.698	0.0123
6	△insertion loop	UBA1-Ub	0.735	0.0109

Rev. Fig. 1 | Binding of $BIRC6^{UBC}$ and $BIRC6^{UBC\Delta insertion\ loop}$ variant to free E1s and ubiquitin-loaded E1 enzymes. a, Microscale thermophoresis (MST) analysis of binding of $BIRC6^{UBC}$ to UBA6-Ub, UBA1-Ub, UBA1 and UBA6. MST binding data points are presented as mean ± s.d. of technical triplicates. Binding affinities determined from two independent experiments. **b,** MST analysis of binding of $BIRC6^{UBC}$ and $BIRC6^{UBC\ \Delta insertion\ loop}$ to UBA1-Ub. The fluorescent tracer in the MST assays was the labelled E1 protein or the corresponding E1-Ub species. **c-h**, Bio-layer interferometry (BLI) analysis of binding of $BIRC6^{UBC}$ to UBA6 (**c**), UBA1 (**d**), UBA6-Ub (**e**) or UBA1-Ub (**f**) , and of the $BIRC6^{UBC\Delta insertion\ loop}$ to UBA6-Ub (g) and UBA1-Ub (h). **i,** Summary of dissociation constants derived from MST curve fitting. **j,** Summary of dissociation constants derived from BLI assays. Binding affinities determined from two independent experiments.

The authors suggest that the insertion loop in the BIRC6 UBC domain clashes with the gate loop of UBA1. Consistent with this, introducing the UBA6 gate loop into UBA1 restores activity (Fig. 4J), and UBA1 can charge the BIRC6 UBC domain lacking the insertion loop (Fig. 4H). If this is the reason that UBA1 does not function with BIRC6, the authors should also test whether UBA6 containing the UBA1 gate loop can work with the BIRC6 UBC domain lacking the insertion loop. This experiment would strengthen their model that specificity is determined by the combined effects of the gate loop and the insertion loop.

Response: We thank the reviewer for this insightful suggestion. Following the reviewer's suggestion, we expressed the $UBA6^{GH\text{-}UBA1*}$ chimera, in which the gate helix of UBA6 was replaced with that of UBA1. However, as shown in **Rev. Fig. 2a**, this chimeric E1 was catalytically inactive in the initial E1~Ub thioester formation assay. Consistently, using the promiscuous E2 UbcH5c (which accepts ubiquitin from both wild-type UBA1 and UBA6), $UBA6^{GH\text{-}UBA1*}$ failed to catalyze UbcH5c~Ub formation in a E1-E2 transthioesterification assay (**Rev. Fig. 2b, d**). Thus, the $UBA6^{GH\text{-}UBA1*}$ chimera

is defective in the ubiquitin transfer cascade, precluding any further transthioesterification assays with $BIRC6^{UBC\text{-}\Delta insertion\ loop}$.

Although we could not perform the exact experiment requested, we completed the reciprocal combinatorial test. By performing a time-course quantitative comparison of the transthioesterification activity of $UBA1^{WT}$, $UBA6^{WT}$, and $UBA1^{GH\text{-}UBA6*}$(the gate helix of UBA1 was replaced with that of UBA6) toward the $BIRC6^{UBC}$. Notably, although the $UBA1^{GH\text{-}UBA6*}$ chimera exhibited a 50% reduction in E1 activity compared to $UBA1^{WT}$, it partially restored transthioesterification activity toward to $BIRC6^{UBC}$, achieving approximately 25% of the efficiency observed with $UBA6^{WT}$, whereas $UBA1^{WT}$ showed no detectable activity (**Rev. Fig. 2c-e**). Furthermore, truncation of the insertion loop in the $BIRC6^{UBC}$ ($BIRC6^{UBC\Delta insertion\ loop}$), partially rescued UBA1-mediated ubiquitin charging, recovering approximately 60% of the activity relative to the UBA6–$BIRC6^{UBC\Delta insertion\ loop}$ pair (**Rev. Fig. 2f, g**).

In conclusion, while the loss of $UBA6^{GH\text{-}UBA1*}$ activity precludes the specific combination requested, the positive result from the $UBA1^{GH\text{-}UBA6*}$+$BIRC6^{UBC}$ experiment, together with the $UBA1^{WT}$+$BIRC6^{UBC\Delta insertion\ loop}$ collectively support the model that the gate helix and insertion loop are as important contributors of UBA6–BIRC6 specificity. We have revised the manuscript to acknowledge this limitation while presenting the new data that could be obtained.

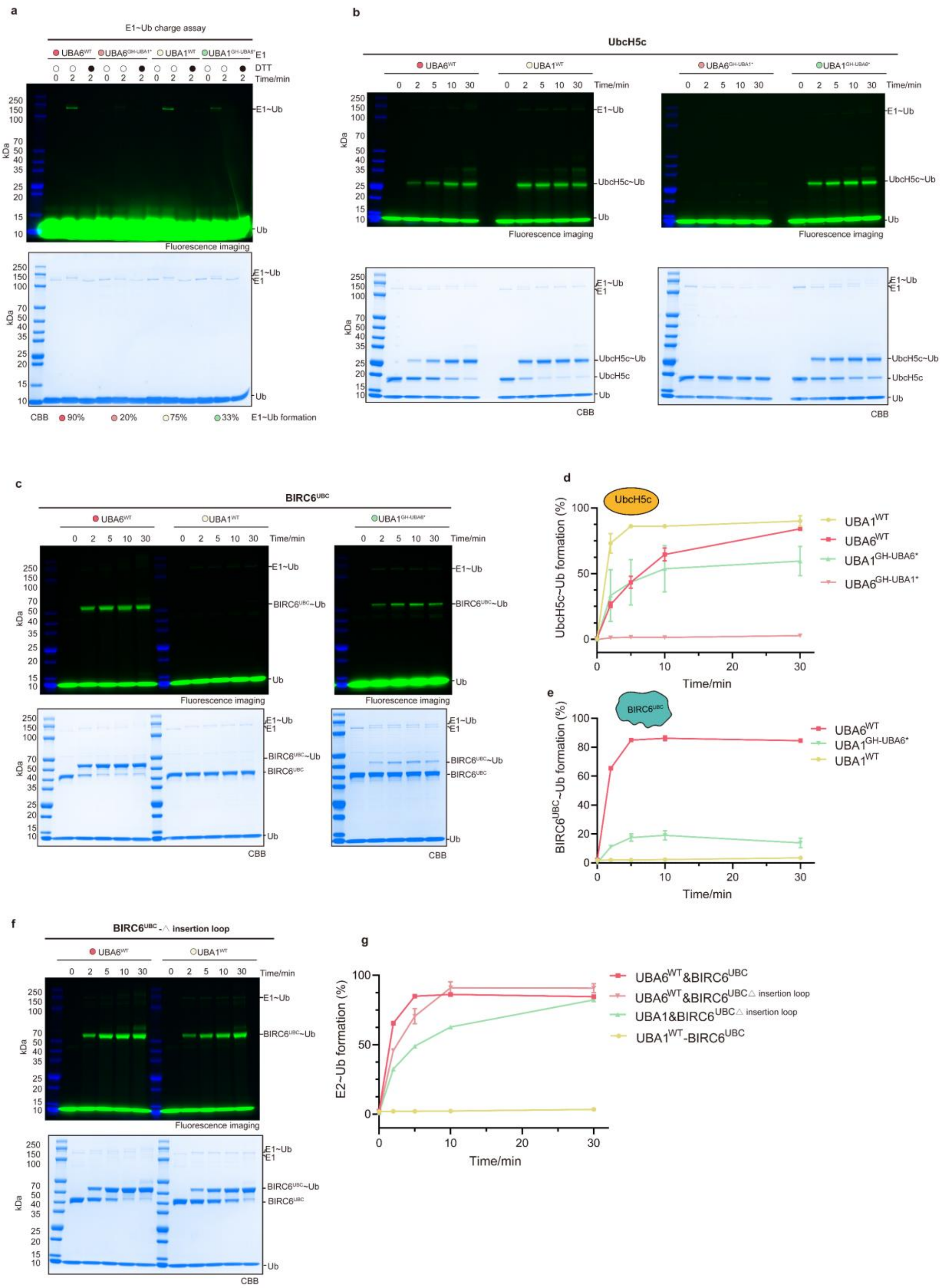

Rev. Fig. 2 | Enzymatic activities of E1 gate-helix chimeras and BIRC6^{UBC} variants. **a**, E1~Ub charging assay comparing UBA6^{WT}, UBA1^{WT}, UBA6$^{GH-UBA1*}$ and UBA1$^{GH-UBA6*}$. Reaction time: 2 min. **b**, E1-E2 transthioesterification assays using UBA6^{WT}, UBA1^{WT}, UBA6$^{GH-UBA1*}$ or UBA1$^{GH-UBA6*}$as the E1 enzyme and UbcH5c as the E2 enzyme. Time points: 0, 2, 5, 10, and 30 min. **c**, E1-E2 transthioesterification assays using UBA6^{WT}, UBA1^{WT} or UBA1$^{GH-UBA6*}$ as the E1 enzyme and BIRC6^{UBC} as the E2 enzyme. Time points: 0, 2, 5, 10, and 30 min. **d-e**, Quantification of the Ubch5c~Ub formation in (**b**) and the BIRC6^{UBC}~Ub in (**c**), respectively. Data

are presented as mean ± s.d. from two independent biological experiments. **f**, E1-E2 transthioesterification assays using UBA6^{WT} or UBA1^{WT} as the E1 enzyme and BIRC6$^{UBC\Delta insertion\ loop}$ as the E2 enzyme. Time points: 0, 2, 5, 10, and 30 min. **g**, Quantification of the BIRC6^{UBC}/BIRC6$^{UBC\Delta insertion\ loop}$~Ub formation for the indicated E1-E2 pairs. Data are presented as mean ± s.d. from two independent biological experiments. In these biochemical assays, fluorescently labelled ubiquitin was used, and both fluorescent signals and the corresponding Coomassie Brilliant Blue-stained gels are shown; quantification was based on the Coomassie Brilliant Blue-stained gels and calculated as the percentage of Ub-charged E2 enzyme relative to total E2 enzyme.

In Figure 3I, the authors suggest that the mutations reduce charging because they weaken the interactions between the proteins. However, one cannot rule out the possibility that some mutations disrupt the overall structure of the protein, which could also reduce charging. Please provide insight into whether the mutant proteins remain properly folded.

Response: We thank the reviewer for raising this important point. Our size exclusion chromatography (SEC) profiles showed that all BIRC6^{UBC} mutants exhibited elution volumes like that of the WT BIRC6^{UBC} (**Rev. Fig. 3a**). Moreover, we performed circular dichroism (CD) spectroscopy on both WT and BIRC6^{UBC} mutant. The CD spectra of the mutants were comparable to that of the WT BIRC6^{UBC}, demonstrating that they retain their native secondary structure (**Rev. Fig. 3b**).

Taken together, these data demonstrate that all mutant proteins remain properly folded. Therefore, the observed reduction in charging activity can be attributed to the specific weakening of interactions at the protein-protein interface, rather than to global structural perturbations.

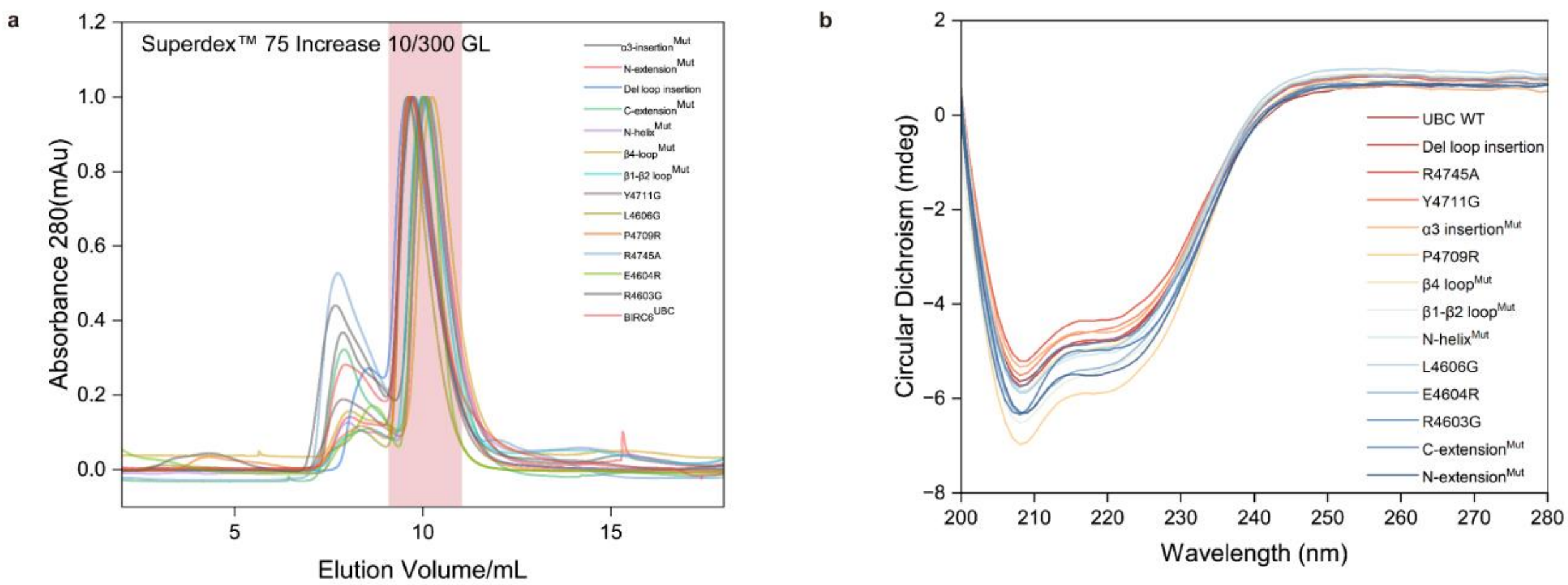

Rev. Fig. 3 | Biophysical characterization of BIRC6^{UBC} variants. a, Size exclusion chromatography (SEC) profiles of all BIRC6^{UBC} mutants. **b**, Circular dichroism (CD) spectras of all BIRC6^{UBC} mutants.

In Figure 5A, the authors test the binding of BIRC6 to UBA1 or UBA6 and suggest that BIRC6 has similar affinity for both. They further propose that the reason BIRC6 is not functional with UBA1 is not lack of binding but restricted movement of the UFD, which blocks charging. If that is the case, then UBA1~Ub should also be able to bind BIRC6. Please repeat the binding experiments shown in Figure 5A using UBA1~Ub and UBA6~Ub. This will clarify whether the charged forms of the E1s display similar binding affinities to BIRC6.

Response: We thank the reviewer for this valuable suggestion, which is important for refining our proposed model. Because the E1~Ub thioester is unstable and prone to hydrolysis, we prepared oxyester-linked UBA1-Ub and UBA6-Ub conjugates (where "-" denotes the oxyester linkage). Using microscale thermophoresis (MST), we further compared the binding affinities of the BIRC6^{UBC} for

UBA1-Ub and UBA6-Ub. The results showed that BIRC6^{UBC} bound UBA6-Ub with an affinity of approximately 1.53±0.22μM, which is about 9-fold stronger than its affinity for UBA1-Ub (13.58 ±1.6μM) (**Rev. Fig. 1a, b**). In addition, we performed orthogonal binding assays using bio-layer interferometry (BLI) to measure the binding affinities between BIRC6^{UBC} and UBA1, UBA6, UBA1-Ub, and UBA6-Ub. While BIRC6^{UBC} bound UBA1 and UBA6 with comparable affinities, it showed stronger binding to UBA6-Ub than to UBA1-Ub (**Rev. Fig. 1c, d, e, f, j**). **These results indicate that BIRC6 preferentially binds the catalytically active form of its cognate E1-Ub conjugate, UBA6-Ub, but not UBA1-Ub.**

Based on these findings, we have revised our model as follows: BIRC6 preferentially binds cognate UBA6-Ub over UBA1-Ub. Subsequently, the gate helix of UBA6 adopts a distinct conformation that is structurally configured to accommodate the insertion loop of BIRC6^{UBC}, thereby enabling efficient transfer of activated ubiquitin from the UBA6 to BIRC6^{UBC}.

In the in vitro transthioesterification assays using fluorescently labeled Ub, please show the full gel so that charging of the E1 is also visible. This is especially important in the gels where BIRC6 charging does not occur, since confirming that the E1 is charged is essential for interpreting these results.

Response: We thank the reviewer for this important point regarding data transparency. In response, we have made the following revisions to all in vitro transthioesterification assays presented in the manuscript:

- **Full Gels:** We now show the complete, uncropped fluorescence gels, including the region corresponding to E1~Ub charging. This ensures that the activity of the E1 is clearly visible for every condition.
- **Loading Controls:** Corresponding Coomassie Blue-stained gels are now included directly below each fluorescent gel to confirm equal protein loading and sample integrity. Moreover, the Coomassie staining also clearly visualizes the E1~Ub species.
- **Uncropped Images:** All original, uncropped gel images have been provided in the Source Data for full transparency.

In Figure 5B, the authors suggest that the decreased charging of BIRC6 with Ub by UBA6 in the presence of UBA1 is due to binding of UBA1 to BIRC6, thereby sequestering BIRC6 from UBA6. However, based on Figure 5B, one cannot exclude the possibility that inhibition arises because UBA1 becomes charged with Ub, which reduces the pool of free Ub. To address this, the authors should repeat the experiment using a catalytically inactive UBA1.

Response: We thank the reviewer for raising this point. To address the concern that inhibition may arise from UBA1 consuming free ubiquitin or ATP, we generated a catalytically inactive UBA1 C623A mutant (active-site Cys623 mutated to Ala), an ATP-binding-deficient mutant referred to as UBA1 ATP mutant (containing D576A, K528E, and R515A mutations), and used UBA1 WT as a control. We tested their effects on the UBA6–BIRC6^{UBC} transthioesterification reaction. The results showed that all three UBA1 variants (C623A, ATP mutant, and WT) inhibited BIRC6^{UBC}~Ub formation to varying extents **(Rev. Fig. 4a, b)**. This indicates that the inhibitory effect is not due to sequestration of free ubiquitin and ATP by UBA1.

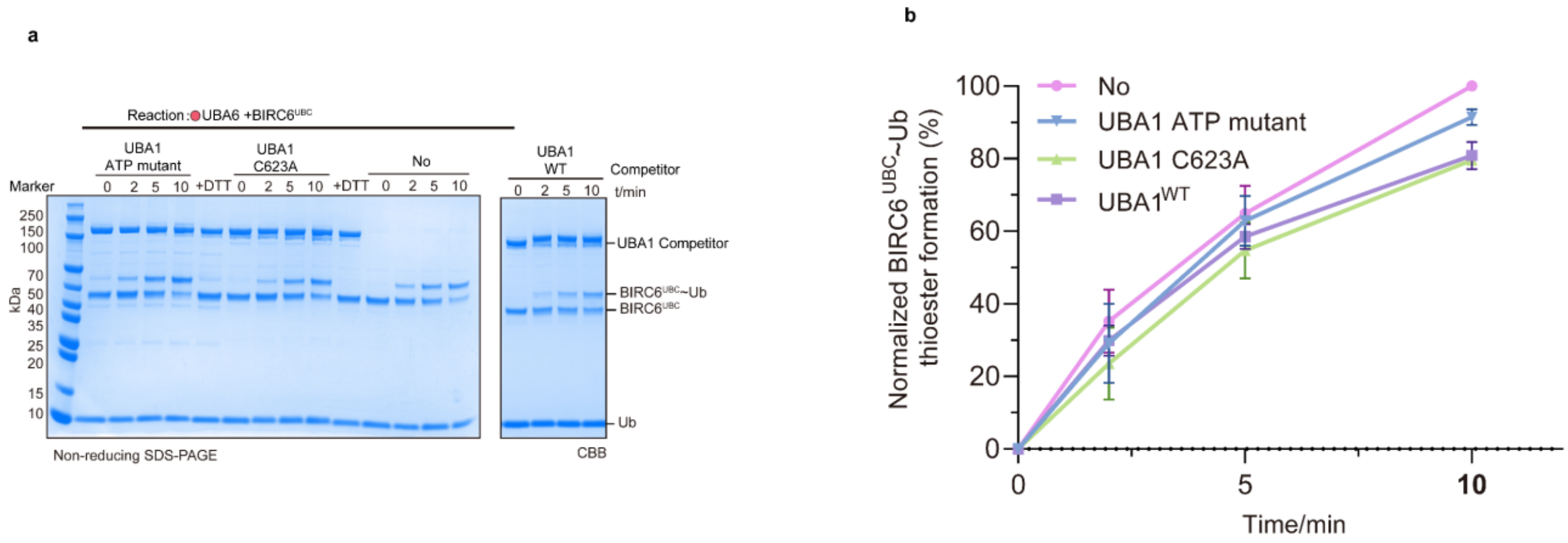

Rev. Fig. 4 | UBA1 variants compete with UBA6 in BIRC6^{UBC} transthioesterification. a, Biochemical analysis of competition between UBA6 and UBA1 variants during BIRC6^{UBC} transthioesterification. UBA1 variants include BA1 C623A mutant (active-site Cys623 mutated to Ala), UBA1 ATP mutant (ATP-binding-deficient, containing D576A, K528E, R515A mutations), and UBA1^{WT}. Reactions contained 0.1 μM UBA6 and 5 μM of UBA1^{WT}, UBA1 C623A, or UBA1 ATP mutant. Time points: 0, 2, 5, 10 min. **b,** Quantification of the BIRC6^{UBC}~Ub shown in (a). Data are presented as mean ± s.d. from two independent biological experiments. Quantification was performed on Coomassie Brilliant Blue-stained gels, and the percentage of BIRC6^{UBC}~Ub was calculated as the ratio of the charged form to total BIRC6^{UBC} (uncharged + charged). Relative activities were then obtained by normalizing to UBA1^{WT}.

Reviewer #2:

The paper by Tong and colleagues reports the structure of the non-canonical ubiquitin-activating E1 enzyme, UBA6, bound to the E2 BIRC6. Mammals encode two genes for ubiquitin activating E1 enzymes: UBA1 and UBA6 and while UBA1 is seen as the most relevant E1 for performing most cellular ubiquitination events the roles of UBA6 are not well understood. By generating a ubiquitin dehydroalanine probe, the authors cleverly trap ubiquitin, UBA6, and BIRC6 as a ternary complex and use these structures to understand why BIRC6 can only work with UBA6, not UBA1. A study into ubiquitin E1-E2 specificity was recently published in NSMB (PMID: 41350950) that captured structures of BIRC6 bound to UBA6 and ubiquitin through an identical strategy. Importantly, that study reports different modes of BIRC6 interaction and specificity with UBA6. Some of the conclusions from this manuscript do not agree with the other published study.

Response: We thank the reviewer for raising this point and for acknowledging the value of our work. We have carefully read the recent study by Riechmann et al. published in NSMB (*Nat. Struct. Mol. Biol.* **2026,** 33, 464–478; PMID 41350950) and have incorporated a detailed discussion of it into our revised manuscript.

Our study does not propose a fundamentally different model but instead **focuses on a distinct yet cooperative aspect of UBA6–BIRC6 specificity.** Both Riechmann et al. and Nayak et al. used domain swap experiments between UBA1 and UBA6 to demonstrate that both the UFD and SCCH domains are critical for BIRC6 specificity toward UBA6 (*Nat. Struct. Mol. Biol.* **2026,** 33, 464–478; PMID 41350950; *Nat. Commun.* 2026, PMID 41764162). Through structural and biochemical analyses, Riechmann et al. further elucidated the molecular mechanism by which the UFD domain of UBA6 contributes to this specificity.

Our study complements these findings by revealing the role of the SCCH domain. The gate helix within the UBA6 SCCH domain adopts a distinct conformation structurally configured to accommodate an insertion loop of $BIRC6^{UBC}$. In contrast, the corresponding helix in UBA1 extends inward toward the E2, causing steric clashes with the insertion loop of BIRC6. **Thus, our work is complementary rather than contradictory to that of Riechmann et al.** Together, the two studies elucidate distinct layers of the UBA6–BIRC6 specificity mechanism, with the UFD governing partner selection and the SCCH optimizing catalytic geometry, thereby providing a more complete mechanistic framework.

This manuscript under review lacks rigorous controls and suffers from several instances of data overinterpretation. There is also a lack of transparency of different assay conditions used which would alter the conclusions reached. There is no source data of uncropped gels or coomassie-stained gels for validation of loading and the reader is left to qualitatively interpret fluorescent loading, sometimes between different gels. There are also numerous mistakes in the writing and presentation of the manuscript (for example multiple occasions of BRIC6 instead of BIRC6). In its present form, I cannot recommend this manuscript for publication and would expect to see major revisions to address the discrepancies in binding between UBA1 and UBA6 and the proposed mutations that are believed to alter E1 specificity of BIRC6.

Response: We thank the reviewer for their thorough and constructive critique, which has helped us

improve the rigor and transparency of our manuscript. We have carefully addressed each point as follows:

1. Lack of rigorous controls and data overinterpretation

We acknowledge the reviewer's concern regarding experimental rigor. To address this, we have made the following revisions:

Additional controls:

1) For the biochemical validation of the gate helix and insertion loop that determine UBA6–BIRC6^{UBC} pairing specificity (**Fig. 4h, i**), we followed the reviewer's suggestion and included E1~Ub charging assays as well as experiments using the general E2 UbcH5c to comprehensively assess the activity changes of the four E1 chimeras (**Extended Fig. 8f**). Moreover, we included multiple time points to compare activity changes, avoiding false-positive results from single time-point measurements.
2) For MST binding assays, we included the general E2 UbcH5c as a control to confirm that the assay can detect physiologically relevant E1–E2 interactions. We also performed orthogonal binding measurements using bio-layer interferometry (BLI).

These additions ensure that all conclusions are drawn from appropriately controlled experiments.

Revised interpretation: We have carefully re-examined our conclusions and modified several instances where the language was overly speculative. For example, in the *Abstract*, we revised the phrase "*is dictated by*" to "*play a key role in*" to better reflect the cooperative and non-exclusive nature of the identified structural determinants. In the *Results* section, we changed "*establish*" to "*suggest*" when describing the gate helix contribution. Additionally, we replaced "*confirm*" with "*support*" in the statement regarding the binding preference of BIRC6 for UBA6~Ub to avoid overinterpretation of the data.

2. Lack of transparency regarding assay conditions

We apologize for the insufficient description of experimental conditions. In the revised Methods and figure legend sections, we now provide detailed protocols for each assay, including:

- Buffer compositions and pH
- Reaction times points and temperatures
- Protein concentrations for each component
- Detection methods
- Replicates and statistical analysis
- Data processing and quantification methods

We believe this level of detail now allows readers to fully reproduce our experiments.

3. Uncropped gels and Coomassie-stained loading controls

For all biochemical experiments presented in the manuscript, we now display the full fluorescence gel images with the corresponding Coomassie-stained gels shown directly below to confirm equal loading. All uncropped original gel images have been compiled and are provided in the **Source data**.

Quantitative interpretation: All comparisons and quantifications between conditions are now performed within the same gel. Quantification was performed by densitometry of Coomassie-stained bands, with at least two independent replicates for each experiment.

4. Writing errors and presentation

We sincerely apologize for the typographical errors, including the repeated misspelling of BIRC6 as "BRIC6." We have carefully proofread the entire manuscript and corrected all such errors. All figure legends have been checked for consistency with the revised figures.

5. Reconciliation of binding discrepancies between UBA1 and UBA6

Regarding the differences in binding affinities, we acknowledge that this is the most notable distinction between the studies. Riechmann et al. reported by size-exclusion chromatography that BIRC6 forms a stable complex only with UBA6, whereas no stable complex was detected with UBA1. Using fluorescence polarization (FP), they further measured the binding affinity between $BIRC6^{UBC}$ and UBA6 to be approximately 200 ± 58 nM; binding to UBA1 was not assessed. In contrast, our MST experiments yielded K_D values of 23 ± 4 μM of $BIRC6^{UBC}$ binding to UBA6. Additionally, our newly performed BLI measurements gave K_D values of 1.316 ± 0.02 μM.

(1) Differences in affinity values obtained by FP, MST, and BLI

Different binding affinity techniques often yield distinct numerical values due to differences in their underlying principles. Factors such as buffer composition, temperature, and whether the interaction occurs in free solution or on a surface also contribute to these variations. Typically, differences within a 3- to 10-fold range are considered acceptable. Here, we employed two orthogonal methods (MST and BLI) to assess binding affinity. We therefore consider our results to be robust and meaningful.

(2) Why SEC showed no co-elution despite comparable binding affinities

SEC and direct affinity measurements probe different aspects of protein interactions. SEC reflects complex stability and hydrodynamic behavior, capturing how long the complex persists and its overall shape. In contrast, MST and BLI measure equilibrium binding affinity, representing how tightly the proteins interact at the initial binding step. Thus, while our MST and BLI data indicate similar apparent affinities of $BIRC6^{UBC}$ for both UBA1 and UBA6, these measurements capture the binding energy at the initial interaction. SEC reports on complex stability during prolonged separation and may be influenced by conformational states that affect complex persistence. The two sets of findings are not contradictory but rather provide complementary perspectives.

(3) Effect of ubiquitin-loaded E1 on binding

We further assessed the binding of $BIRC6^{UBC}$ to UBA1~Ub and UBA6~Ub by both MST and BLI (**Rev. Fig. 5 a, c, d, e, f, i, j**). The results showed that when using oxyester-linked UBA1-Ub and UBA6-Ub conjugates to mimic the catalytically charged E1 state (where "-" denotes the oxyester linkage), BIRC6 binding to UBA6-Ub was enhanced, with an approximately 9-fold increase by MST and a 2-fold increase by BLI, whereas binding to UBA1-Ub remained unchanged. This indicates that under catalytically active conditions, $BIRC6^{UBC}$ preferentially binds UBA6-Ub, consistent with the conclusion by Riechmann et al. that a stable interaction occurs only with its cognate E1, UBA6.

Moreover, truncation of the insertion loop in BIRC6^{UBC} enhanced binding to UBA1-Ub, further supporting a critical role for the BIRC6 insertion loop in mediating UBA6 specificity (**Rev. Fig. 5 b, g, h, i, j**).

Based on these results, we have further refined our mechanistic model. BIRC6 preferentially binds its cognate E1, UBA6-Ub, over UBA1-Ub. The gate helix of UBA6 adopts a distinct conformation that is structurally configured to accommodate the insertion loop of BIRC6^{UBC}. This refined model has been incorporated into the revised ***Discussion section.*** We have also cited the studies by Riechmann et al. and Nayak et al., and explicitly state that together they reveal distinct molecular contributors of UBA6–BIRC6 specificity, collectively advancing our understanding of E1–E2 specificity regulation.

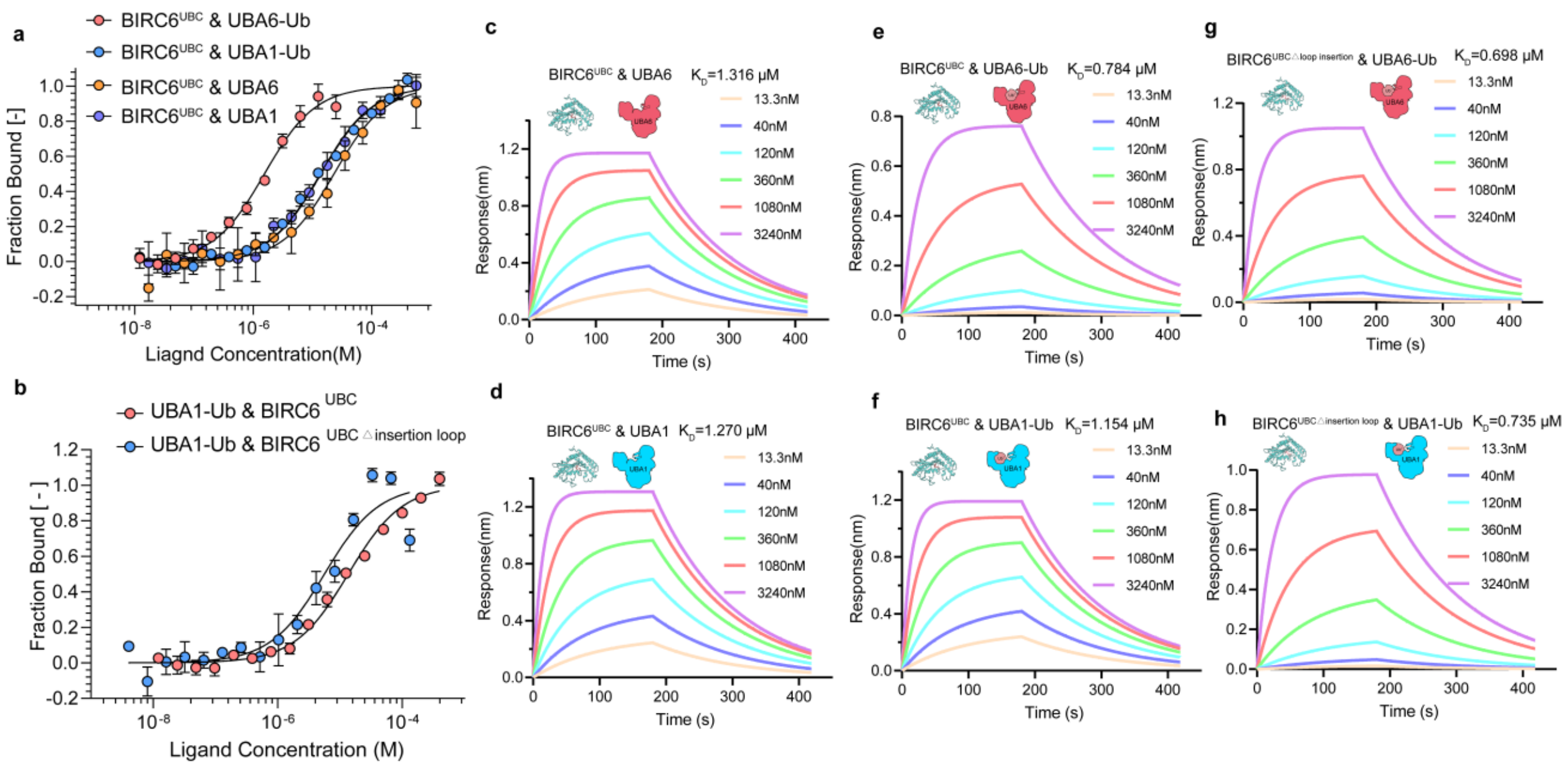

i

Summary of MST binding affinity data

Group	BIRC6^{UBC}	E1	K_D(μM)	K_D confidence (μM)
1	WT	UBA6	23.02	±4.29
2	WT	UBA1	14.00	±1.66
3	WT	UBA6-Ub	1.53	±0.22
4	WT	UBA1-Ub	13.58	±1.6
5	△insertion loop	UBA1-Ub	5.61	±2.11

j

Summary of BLI binding affinity data

Group	BIRC6^{UBC}	E1	K_D(μM)	K_D error (μM)
1	WT	UBA6	1.316	0.0218
2	WT	UBA1	1.27	0.0198
3	WT	UBA6-Ub	0.784	0.011
4	WT	UBA1-Ub	1.154	0.019
5	△insertion loop	UBA6-Ub	0.698	0.0123
6	△insertion loop	UBA1-Ub	0.735	0.0109

Rev. Fig. 5 | Binding of BIRC6^{UBC} and BIRC6$^{UBC\Delta insertion\ loop}$ variant to free E1s and ubiquitin-loaded E1 enzymes. a, Microscale thermophoresis (MST) analysis of binding of BIRC6^{UBC} to UBA6-Ub, UBA1-Ub, UBA1 and UBA6. MST binding data points are presented as mean ± s.d. of technical triplicates. Binding affinities determined from two independent experiments. **b,** MST analysis of binding of BIRC6^{UBC} and BIRC6$^{UBC\ \Delta insertion\ loop}$ to UBA1-Ub. The fluorescent tracer in the MST assays was the labelled E1 protein or the corresponding E1-Ub species. **c-h**, Bio-layer interferometry (BLI) analysis of binding of BIRC6^{UBC} to UBA6 (**c**), UBA1 (**d**), UBA6-Ub (**e**) or UBA1-Ub (**f**) , and of the BIRC6$^{UBC\Delta insertion\ loop}$ to UBA6-Ub (g) and UBA1-Ub (h). **i,** Summary of dissociation constants derived from MST curve fitting. **j,** Summary of dissociation constants derived from BLI assays. Binding affinities determined from two independent experiments.

6. Proposed mutations that alter E1 specificity of BIRC6

To address concerns that the observed specificity effects might arise from general defects in E1 activity rather than specific interface disruptions, we quantitatively evaluated the activities of UBA1,

UBA6, UBA6$^{GH\text{-}UBA1*}$, and UBA1$^{GH\text{-}UBA6*}$ using E1~Ub charging assays and E1-E2 transthioesterification assays with the promiscuous E2 UbcH5c as a control (**Rev. Fig. 6a, b, d**). The UBA6$^{GH\text{-}UBA1*}$ chimera was inactive in both assays, precluding further analysis. In contrast, the UBA1$^{GH\text{-}UBA6*}$ chimera retained approximately 50% of wild-type UBA1 activity toward UbcH5c, indicating that its general E1 function is only partially impaired. Despite this reduction, UBA1$^{GH\text{-}UBA6*}$ partially restored transthioesterification toward BIRC6^{UBC}, achieving about 25% of UBA^{WT} efficiency, whereas UBA1^{WT} showed no detectable activity (**Rev. Fig. 6c, e**).

Furthermore, deletion of the BIRC6^{UBC} insertion loop (BIRC6$^{UBC\Delta insertion\ loop}$) did not compromise its intrinsic transthioesterification activity toward UBA6, but it partially rescued UBA1-mediated ubiquitin charging (**Rev. Fig. 6f, g**). In conclusion, although the loss of UBA6$^{GH\text{-}UBA1*}$ E1 activity precluded reciprocal validation, both UBA1$^{GH\text{-}UBA6*}$ and BIRC6$^{UBC\Delta insertion\ loop}$ partially restored transthioesterification activity, collectively supporting a model in which the gate helix and insertion loop are critical contributors of UBA6–BIRC6 ubiquitin transfer specificity.

We believe these revisions have substantially improved the manuscript and thank the reviewer again for their valuable input.

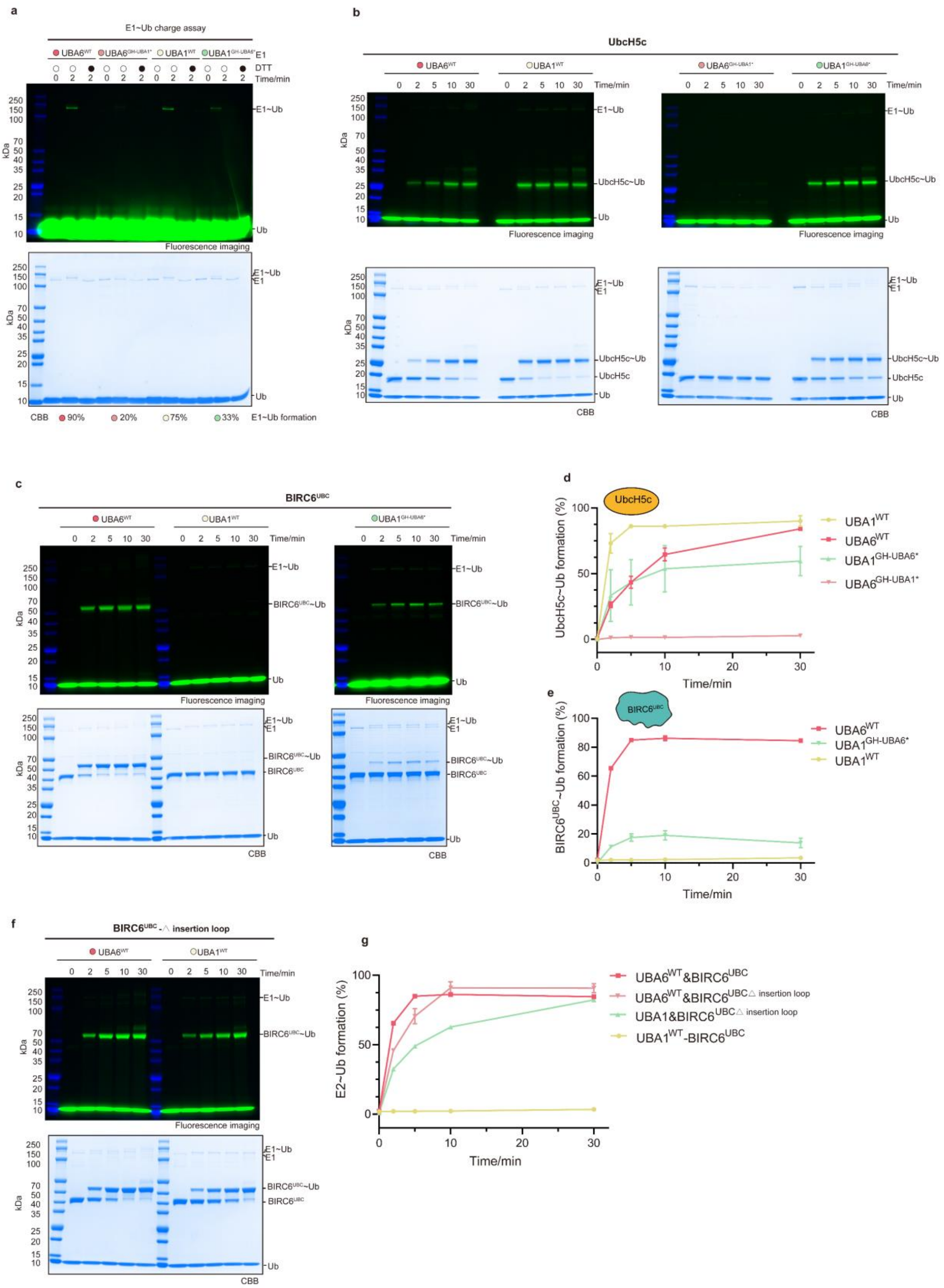

Rev. Fig. 6 | Enzymatic activities of E1 gate-helix chimeras and BIRC6^{UBC} variants. a, E1~Ub charging assay comparing UBA6^{WT}, UBA1^{WT}, UBA6$^{GH\text{-}UBA1*}$ and UBA1$^{GH\text{-}UBA6*}$. Reaction time: 2 min. **b**, E1-E2 transthioesterification assays using UBA6^{WT}, UBA1^{WT}, UBA6$^{GH\text{-}UBA1*}$ or UBA1$^{GH\text{-}UBA6*}$as the E1 enzyme and UbcH5c as the E2 enzyme. Time points: 0, 2, 5, 10, and 30 min. **c**, E1-E2 transthioesterification assays using UBA6^{WT}, UBA1^{WT} or UBA1$^{GH\text{-}UBA6*}$ as the E1 enzyme and BIRC6^{UBC} as the E2 enzyme. Time points: 0, 2, 5, 10, and 30 min. **d-e**, Quantification of the Ubch5c~Ub formation in (**b**) and the BIRC6^{UBC}~Ub in (**c**), respectively. Data

are presented as mean ± s.d. from two independent biological experiments. **f**, E1-E2 transthioesterification assays using UBA6^{WT} or UBA1^{WT} as the E1 enzyme and BIRC6$^{UBC\Delta insertion\ loop}$ as the E2 enzyme. Time points: 0, 2, 5, 10, and 30 min. **g**, Quantification of the BIRC6^{UBC}/BIRC6$^{UBC\Delta insertion\ loop}$~Ub formation for the indicated E1-E2 pairs. Data are presented as mean ± s.d. from two independent biological experiments. In these biochemical assays, fluorescently labelled ubiquitin was used, and both fluorescent signals and the corresponding Coomassie Brilliant Blue-stained gels are shown; quantification was based on the Coomassie Brilliant Blue-stained gels and calculated as the percentage of Ub-charged E2 enzyme relative to total E2 enzyme.

Specific major points are listed below:

1. For the two states obtained (single or doubly loaded UBA6-BIRC6), it would be helpful for the authors to compare if there were any differences in the position of BIRC6 in the two states. In addition, it was not clear whether ATP was observed in the adenylation domain for the singly-loaded BIRC6-UBA6. Given recent insights from the Lima lab showed coupling of ubiquitin transfer to the adenylation domain (PMID: 39143218) the authors should investigate any differences between the two states.

Response: We thank the reviewer for these insightful suggestions. Structural alignment of the singly and doubly loaded UBA6–BIRC6 complexes, using the UBA6 core as a reference, revealed that the position of the BIRC6^{UBC} domain is nearly identical in both states, with a root-mean-square deviation (RMSD) of 0.633 Å over Cα atoms **(Rev. Fig. 7a)**.

Next, we carefully inspected the density in the ATP catalytic pocket of UBA6 for both complexes. In both structures, the pocket contains extra density that fits AMP well but cannot be modeled as ATP **(Rev. Fig. 7b-e)**. This probably indicates that ATP has been fully hydrolyzed to AMP in the captured states. Additionally, in the singly loaded structure, we observed an extra density adjacent to the AMP that likely corresponds to the C-terminal tail of the adenylated ubiquitin (Ub^{A}).

These observations indicate both the singly and doubly loaded UBA6–BIRC6 complexes we captured closely resemble Cluster 5 of the doubly loaded complexes reported by Kochańczyk et al. (*Nature*, **2024**, 633, 216–223, PMID: 39143218) **(Rev. Fig. 7f)**. In this state, ATP is fully hydrolyzed to AMP, PPi•Mg^{2+} is absent, Ub^{A} is adenylated, and Ub^{T} occupies the donor position. This is consistent with the mechanism proposed by Kochańczyk et al., in which Ub^{A} adenylation and PPi•Mg^{2+} release occur concurrently and are mechanistically coupled with transthiolation. Our structures, captured at an intermediate step in this coupled cycle, provide independent structural support for this model in the context of the UBA6–BIRC6-specific E1–E2 pairing.

We have added a description of these findings to the Results sections of the revised manuscript and have cited the relevant paper (*Nature*, **2024**, 633, 216–223, PMID: 39143218).

"*Inspection of the ATP catalytic pocket in both UBA6–BIRC6^{UBC}–Ub^{T} and UBA6–BIRC6^{UBC}–Ub^{T}–Ub^{A} structures revealed additional density that is compatible with AMP. Furthermore, in the singly loaded state, an extra density likely corresponding to the C-terminal tail of Ub^{A}. These features indicate that these two complexes we captured closely resemble Cluster 5 of the previously reported doubly loaded E1–E2 states (PDB: 9B5I), in which ATP is fully hydrolyzed to AMP, PPi•Mg^{2+} is absent, Ub^{A} is adenylated, and Ub^{T} occupies the donor position. This configuration is consistent with a mechanism wherein Ub^{A} adenylation and PPi•Mg^{2+} release occur concomitantly and are coupled with transthiolation.*"

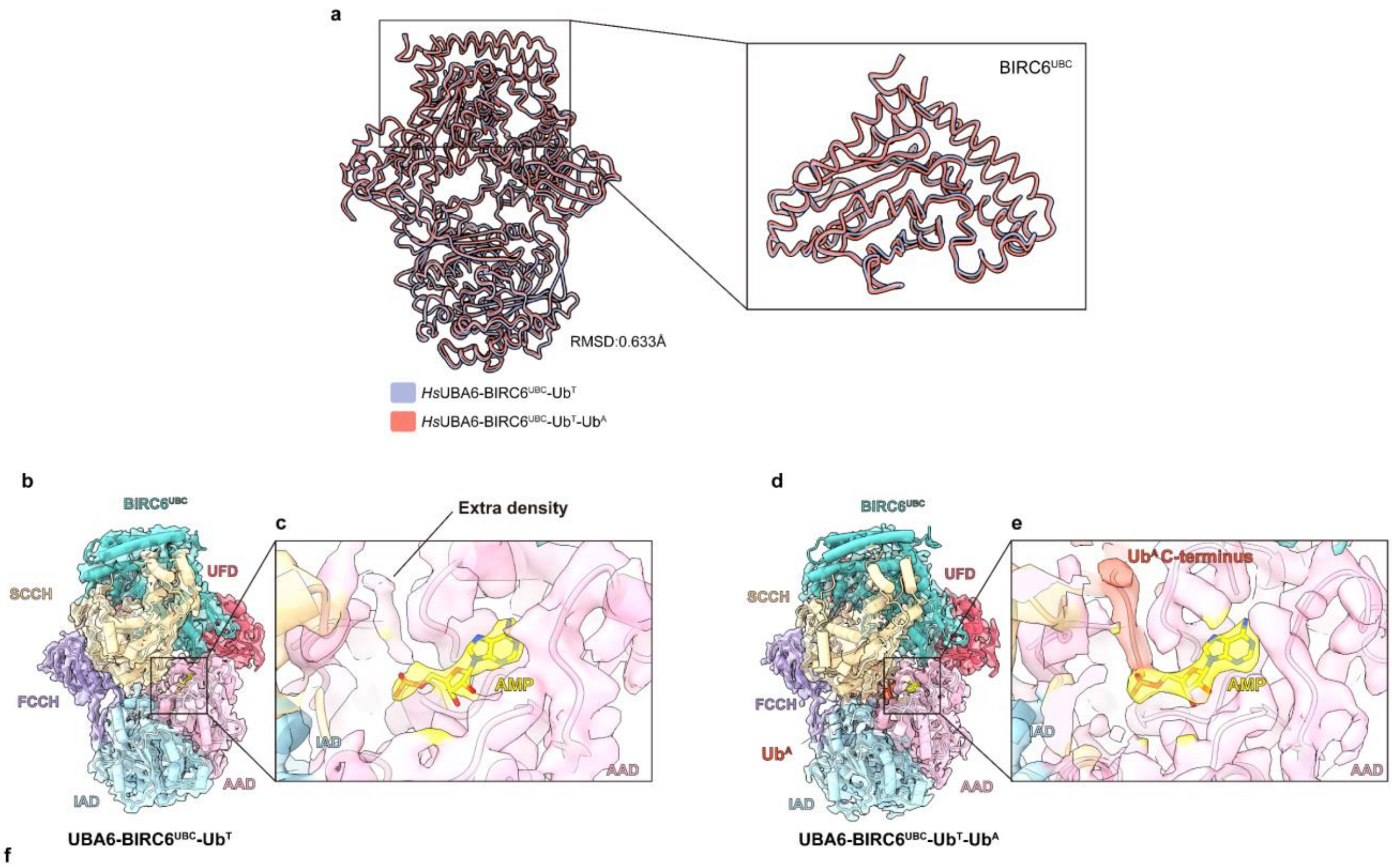

f

[editorial note: third party material redacted]

From PMID: 39143218(For structural comparison only)

Rev. Fig. 7 | Structural analysis of singly and doubly loaded UBA6-BIRC6 complexes. a, Structural alignment of the singly and doubly loaded UBA6–BIRC6 complexes using the UBA6 core as the reference, showing that the BIRC6^{UBC} adopts a nearly identical position in the two states (RMSD = 0.633 Å). **b-e**, Density and model views of the ATP catalytic pocket of UBA6 in the singly and doubly loaded UBA6-BIRC6 complexes. In both structures, the pocket contains density that is well fitted by AMP, and extra density adjacent to AMP likely corresponds to the C-terminal tail of adenylated ubiquitin. **f**, The cluster 5 of the doubly loaded complexes reported by Kochańczyk et al. (*Nature*, **2024**, 633, 216–223, PMID: 39143218)

2. No maps/models have been provided by the authors to confirm quality. It appears from Figure 2d that the E1 Cys is in the wrong configuration to be forming a thioester bond to ubiquitin. The authors need to correct this and demonstrate that they can clearly model the relevant bonding distances and angles in their EM maps. It thus raises whether the rest of the maps have been correctly interpreted.

Response: We thank the reviewer for this observation regarding the structural model quality. We have carefully re-examined our maps and models to address these concerns.

1. Re-modeling of UBA6 C625 side chain

We have now re-built this residue using the higher-resolution local map around the active site.

As shown in **(Rev. Fig. 8a)**, the revised model places the C625 thiol group in a conformation that is fully consistent with the density and compatible with the geometry required for thioester bond formation.

2. Comprehensive model validation

To address the broader concern about model quality, we now provide the following validation metrics and figures:

- Model vs. map correlation: We have generated map-versus-model evaluation figures for UBA6-BIRC6^{UBC}-Ub^{T}-Ub^{A} and UBA6-BIRC6^{UBC}-Ub^{T} structures **(Rev. Fig. 8b)**. In all cases, the density clearly supports the traced backbone and side chain placements.
- Fourier shell correlation (FSC): The overall resolution of the map is 3.3 Å and 3.37 Å at FSC = 0.143 (gold-standard FSC curve shown in **Extended Figure 3a**).
- Model geometry: More than 95% of residues are in favored regions of the Ramachandran plot, with almost no outliers. Additional model statistics, including MolProbity scores and RMSD values for bond lengths and angles, are provided in **Table 1**.

We have updated the model coordinates and deposited them in the PDB. The revised model files and associated validation figures are now included in the revised manuscript.

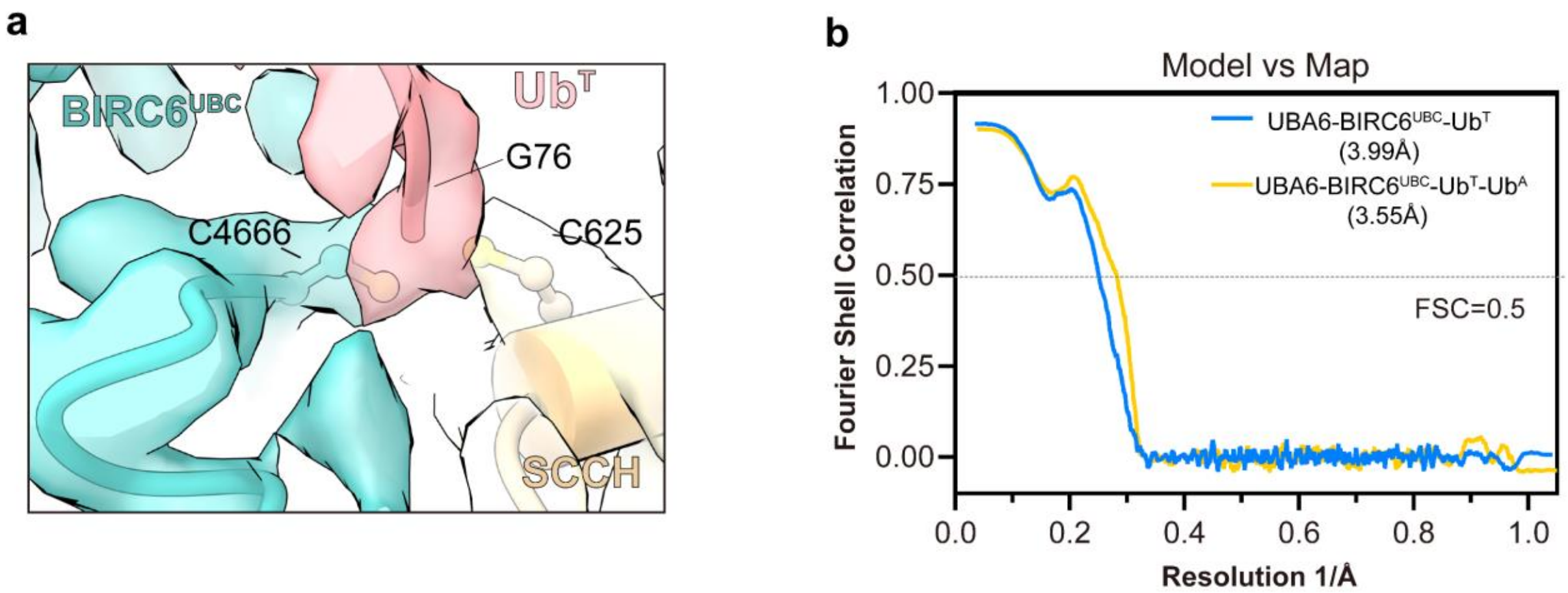

Rev. Fig. 8 | Structural validation of the UBA6-BIRC6^{UBC} model. a, Close-up view of the local cryo-EM density and fitted model at the transthiolesterification center, showing the revised placement of the UBA6 C625 side chain. **b,** Map-versus-model evaluation of the UBA6-BIRC6^{UBC}-Ub^{T}-Ub^{A} and UBA6-BIRC6^{UBC}-Ub^{T}complexes.

3. How was fluorescence quantified in Figure 3h? In all instances throughout the paper, there are no uncropped gels showing the other components present or relevant Coomassie-stained gels confirming equal loading.

Response: We apologize for the lack of detailed quantification description in the previous version. For *Figure 3h*, quantification was performed directly from the Coomassie-stained gels of the same gels used for fluorescence imaging. The efficiency of BIRC6^{UBC}~Ub thioester formation was calculated as the ratio of BIRC6^{UBC}~Ub to total BIRC6^{UBC}. Relative thioester formation was then obtained by normalizing each mutant to wild-type BIRC6^{UBC}. We have now provided a detailed description of the quantification methods for all experiments in both the figure legends and the Methods section.

To improve transparency, we have now displayed all relevant gels in full, with the corresponding

Coomassie-stained gels shown directly below to confirm equal loading **(Rev. Fig. 9)**. Uncropped original gel images for all experiments have also been compiled and provided in the Source data.

4. The authors describe several interfaces between BIRC6 and UBA6, notably within the UBA6 UFD domain, and make mutations within BIRC6 to test the contribution of each of the interfaces. Some of the mutations are conservative loss of functional groups whereas others are charge-swap and in some instances destabilising (introduction of glycine). Can the authors provide a rationale for the choice of mutations made? Also, very few are single amino acid substitutions but rather mutations of several residues at a time - which residues within the b1-b2 loop or a3 insertion particularly affect the BIRC6-UBA6 interaction?

Response: We thank the reviewer for the thoughtful comments on our mutagenesis strategy. All mutations were designed based on the structural interfaces identified in the UBA6–BIRC6 complex. Conservative alanine replacements were introduced to remove specific side-chain interactions (e.g., hydrogen bonds) while minimally perturbing the overall structure; charge-reversal mutations were used to target electrostatically complementary interfaces observed in the structure; and glycine substitutions were introduced to remove side chains, thereby disrupting van der Waals contacts at the interface. We acknowledge that glycine substitutions may affect local stability, but all mutants were verified to be properly folded by size-exclusion chromatography (SEC), and representative mutants were additionally confirmed by circular dichroism spectroscopy (CD) (**Rev. Fig. 9a, b**).

Regarding the use of multi-residue mutations, because the interaction interfaces involve multiple residues rather than isolated hot spots, single substitutions often yield only weak effects. We therefore initially mutated several exposed residues within each region in combination to assess their overall functional contribution. To more precisely map the critical determinants, we subsequently performed single-residue substitutions at key positions within the β1–β2 loop (E4604R, R4605G, L4606G) and the α3 insertion (Y4711G, P4709R, R4745A). The Y4711G mutation resulted in a 60% reduction in BIRC6^{UBC}~Ub formation, whereas P4709R and R4745A showed minimal effects. Mutations within the β1–β2 loop (E4604R, R4605G, L4606G) each reduced Ub charging activity by 10–20%. These experiments revealed that Y4711 within the α3 insertion is the primary contributor to hydrophobic packing, whereas residues E4604, R4605, and L4606 in the β1–β2 loop collectively contribute to the interaction **(Rev. Fig. 9c, d)**. These additional data have been incorporated into the revised manuscript and the Methods section.

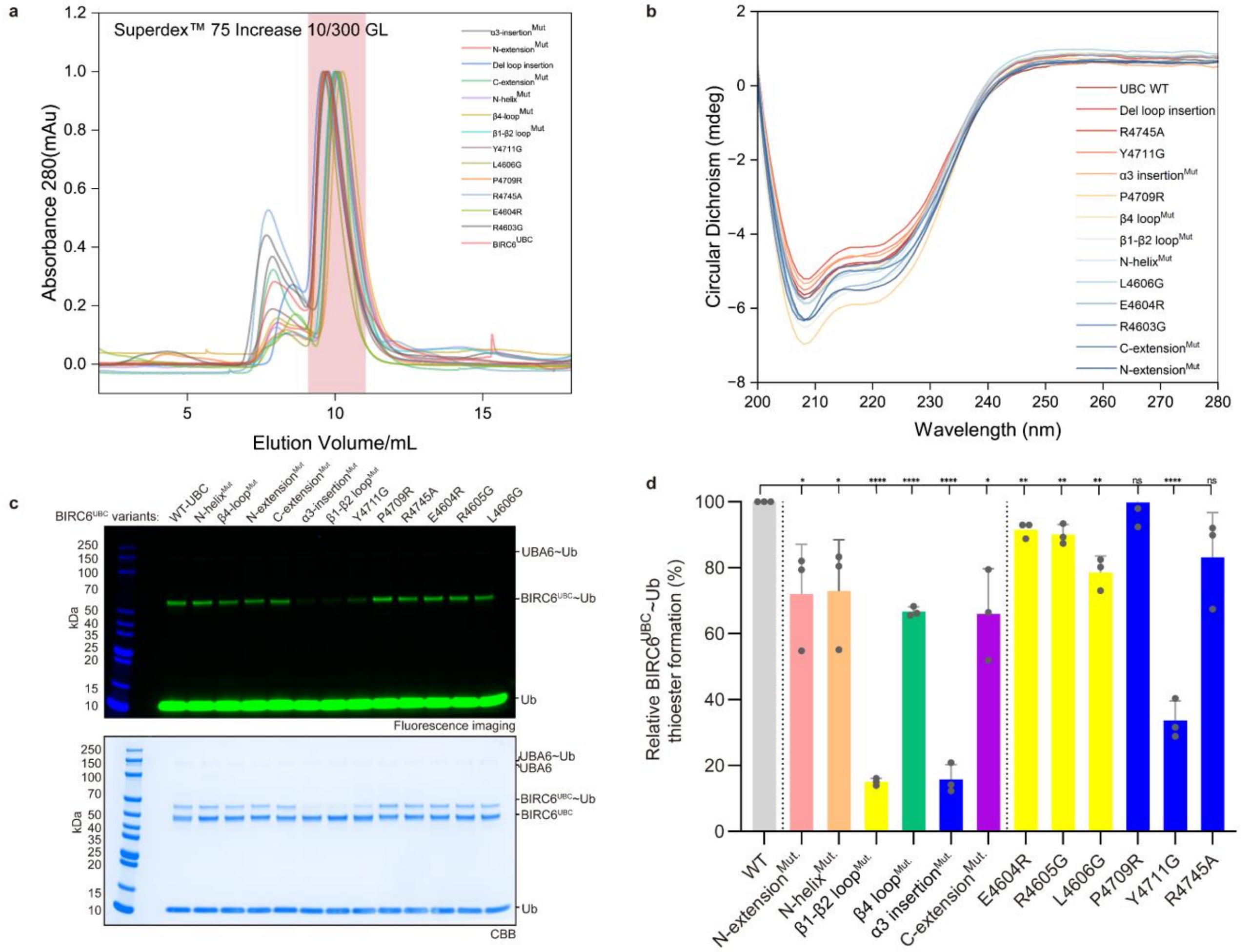

Rev. Fig. 9 | Biophysical characterization of BIRC6^{UBC} variants and biochemical validation of the interactions between UBA6 and BIRC6^{UBC}. a, Size exclusion chromatography (SEC) profiles of all BIRC6^{UBC} mutants. **b**, Circular dichroism (CD) spectra of all BIRC6^{UBC} mutants. **c**, In vitro transthioesterification assays using fluorescently labelled ubiquitin with the indicated BIRC6^{UBC} variants. The gel image is representative of three independent biological experiments. **d**, Bar graph showing the fraction of BIRC6^{UBC}~Ub presented as mean ± s. d. from n = 3 independent experiments. Each data point is shown as a black dot (data from h). A two-sample, two-tailed Student's unpaired t-test was used to calculate p values; ****$p < 0.0001$, ***$p < 0.001$, *$p < 0.05$. Reaction time: 2 min.

5. The authors describe a gate helix within the SCCH domain of UBA1 that is absent in UBA6 as providing a steric clash preventing BIRC6 function. They then make a deletion of the equivalent region in BIRC6 and measure transthiolation activity and observe BIRC6 functioning with UBA1 at levels comparable to UBA6. Transfer from UBA1 onto mutant BIRC6 should be quantified and compared to transfer from UBA6 onto WT/mutant BIRC6.

Response: We thank the reviewer for this suggestion. Following the reviewer's recommendation, we performed a time-course transthioesterification assay for both BIRC6^{UBC} and BIRC6$^{UBC\ \Delta insertion\ loop}$ with UBA1 and UBA6 to avoid potential false positives from single time-point measurements (**Rev. Fig. 10a-c**). Quantification was performed directly from the Coomassie-stained gels. The intensities of the BIRC6^{UBC} and BIRC6^{UBC}~Ub bands were measured, and the efficiency was calculated as the percentage of BIRC6^{UBC}~Ub relative to total BIRC6^{UBC}. The results showed that UBA6 exhibited comparable transthioesterification activity toward both BIRC6^{UBC} and BIRC6$^{UBC\ \Delta\ insertion\ loop}$. UBA1

showed no detectable activity toward BIRC6^{UBC}, but importantly, recovered approximately 60% of the activity toward BIRC6$^{UBC\Delta\ insertion\ loop}$ relative to the UBA6–BIRC6$^{UBC\ \Delta insertion\ loop}$ pair.

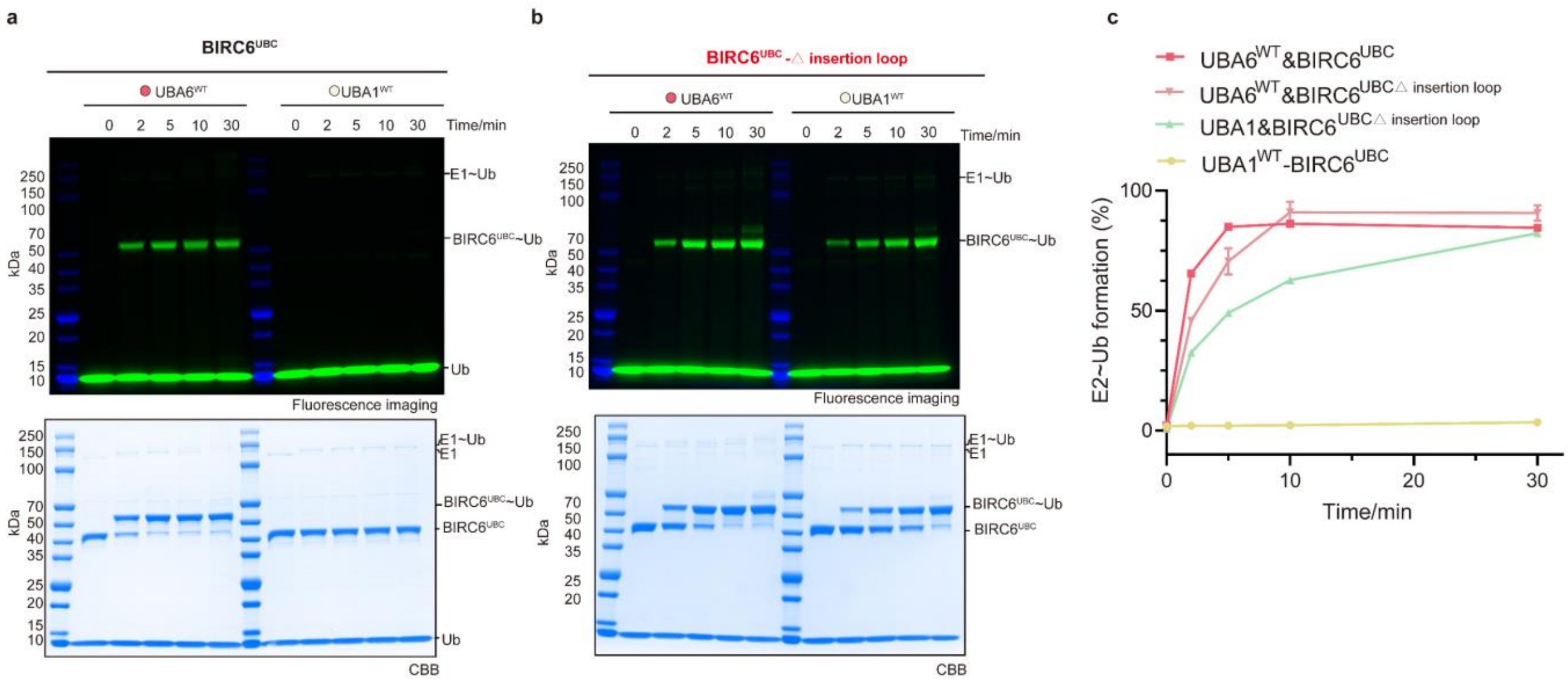

Rev. Fig. 10| E1-E2 transthioesterification assays using BIRC6^{UBC} and BIRC6$^{UBC\Delta insertion\ loop}$ variant. a-b. time-course transthioesterification assays of BIRC6^{UBC}. (**a**) and the BIRC6$^{UBC\Delta insertion\ loop}$ variant (**b**) with UBA1 or UBA6, shown by fluorescent ubiquitin signal and corresponding Coomassie Brilliant Blue-stained gels. Time point: 0, 2, 5, 10, 30min. **c**, Quantification based on the Coomassie Brilliant Blue-stained gels shown in (**a**) and **(b)**. E2~Ub formation (%) was calculated as the percentage of Ub-charged BIRC6^{UBC} species relative to total BIRC6^{UBC} protein. Data are presented as mean ± s.d. from two independent biological experiments.

The authors then make corresponding deletions/insertions in UBA1 and UBA6 to suggest specificity mechanisms. Extended Figure 8 b, c, show that the activity of the E1s for loading ubiquitin is reduced with both UBA1 and UBA6 mutants. Extreme caution should therefore be applied when interpreting any effects on transfer to E2s from these E1 mutants. Reference to this figure and description of the different activities of these E1 mutants is omitted from the main text. The authors need to include more controls to show the mutated E1s are still functional with other E2s that do not contain the insertion loop identified in BIRC6. To determine if general E2 transfer has been altered. An additional important point, the methods section states these specificity swap assays were performed at 37℃ for 30min compared to R.T. for 2min for structural mutants. It is therefore misleading to suggest any of these mutations have altered specificity when left for 15x longer. Quantification of transfer at 2 min should be performed.

Response: We thank the reviewer for pointing out this limitation. We apologize for not discussing this figure in the main text. The revisions and adjustments we have made are as follows:

1. We quantified and comprehensively evaluated the activity of the mutant E1s using E1~Ub charging assays and the promiscuous E2 UbcH5c (**Rev. Fig. 6a, b**). The results showed that the UBA6$^{GH\text{-}UBA1*}$ chimera lost both E1~Ub charging activity and the ability to catalyze UbcH5c~Ub formation, whereas the UBA1$^{GH\text{-}UBA6*}$ chimera retained approximately 50% of the activity observed for wild-type UBA1.

2. To avoid potential false positives from a single time point, we performed time-course experiments for the swap assays using five time points: 0, 2, 5, 10, and 30 min (including the 2 min

time point suggested by the reviewer). Quantification was performed on Coomassie-stained gels, and the efficiency of $BIRC6^{UBC}$~Ub formation was calculated as the ratio of the $BIRC6^{UBC}$ ~Ub band intensity to that of total $BIRC6^{UBC}$. The resulting curves showed that similar results were observed across different time points. Specifically, the $UBA1^{GH\text{-}UBA6*}$ chimera restored transthioesterification activity toward $BIRC6^{UBC}$, whereas $BIRC6^{UBC\Delta insertion\ loop}$ partially rescued UBA1-mediated ubiquitin charging **(Rev. Fig. 6c-g**).

3.We added a description of the changes in E1 activity and included the corresponding figure citation in the main text. The revised text is as follows:

" *We next engineered chimeric E1 enzymes by exchanging the gate helix. Specifically, we generated UBA6, UBA1, a chimera in which UBA6's gate helix was replaced with that of UBA1 (referred to as $UBA6^{GH\text{-}UBA1*}$), and a chimera in which UBA1's gate helix was replaced with that of UBA6 (referred to as $UBA1^{GH\text{-}UBA6*}$). E1~Ub charging assays showed that $UBA6^{GH\text{-}UBA1*}$ exhibited little to no ability to activate ubiquitin, whereas $UBA1^{GH\text{-}UBA6*}$ retained approximately 50% of the activity compared to $UBA1^{WT}$ (**Extended Fig. 8f**). Consistent with this, transthioesterification assays using the promiscuous E2 UbcH5c revealed that $UBA6^{GH\text{-}UBA1*}$ failed to catalyze UbcH5c~Ub formation, while $UBA1^{GH\text{-}UBA6*}$ remained capable of charging UbcH5c, albeit with reduced efficiency (**Extended Fig. 8g, h**). Because the $UBA6^{GH\text{-}UBA1*}$ chimera is defective in the ubiquitin transfer cascade, it precluded further transthioesterification assays with the $BIRC6^{UBC}$. Therefore, we tested only $UBA1^{GH\text{-}UBA6*}$ for its transthioesterification activity toward $BIRC6^{UBC}$. A time-course quantitative comparison of the activities of $UBA1^{WT}$, $UBA6^{WT}$, and $UBA1^{GH\text{-}UBA6*}$ toward $BIRC6^{UBC}$ showed that the $UBA1^{GH\text{-}UBA6*}$ chimera partially restored this activity, whereas $UBA1^{WT}$ showed no detectable charging of $BIRC6^{UBC}$ (**Fig. 4h, Extended Fig. 8i**).*"

6. The authors used microscale thermophoresis to demonstrate that BIRC6 bound to UBA6 and UBA1 with equal affinity. Here it will be important for the authors to validate this through orthogonal means. The other published paper has used fluorescence polarisation to detect the interaction between BIRC6 and UBA6, reporting a nanomolar interaction between the two, and no interaction between BIRC6 and UBA1. Can the authors reconcile their findings? The authors should consider switching the label onto the smaller protein of the interaction pair (i.e. BIRC6) and see if they observe a similar MST response. In addition, they should consider testing the binding of another cross-reactive E2 to UBA1 and UBA6. Also, is the binding of BIRC6 to either UBA1 or UBA6 altered by the presence of ubiquitin loaded onto the E1? Given the author's current MST setup, this will be fairly easy to test and may reveal interesting observations.

Response: We thank the reviewer for these important points regarding our binding studies. We have performed orthogonal validation and additional experiments to further substantiate our conclusions, and have included a detailed discussion of recently published data.

1. Orthogonal validation

We measured the binding affinity of $BIRC6^{UBC}$ for UBA6 and UBA1 by bio-layer interferometry (BLI). The results showed comparable affinities, with K_D values of 1.32 ± 0.02 μM and 1.27 ± 0.02 μM , respectively (**Rev. Fig. 11a-g**).

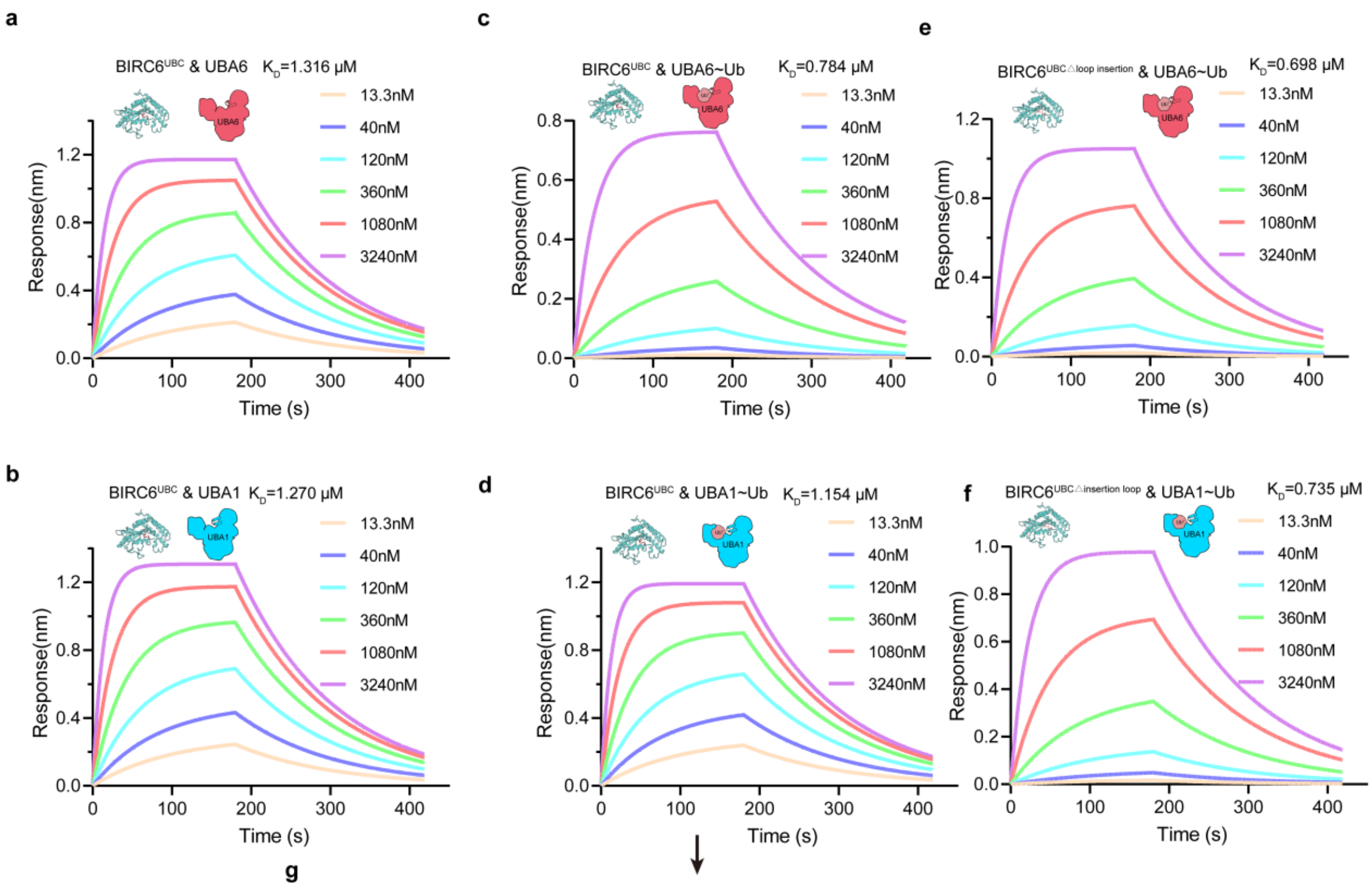

g

Summary of BLI binding affinity data

Group	BIRC6^{UBC}	E1	K_D(μM)	K_D error (μM)
1	WT	UBA6	1.316	0.0218
2	WT	UBA1	1.27	0.0198
3	WT	UBA6-Ub	0.784	0.011
4	WT	UBA1-Ub	1.154	0.019
5	△insertion loop	UBA6-Ub	0.698	0.0123
6	△insertion loop	UBA1-Ub	0.735	0.0109

Rev. Fig. 11 | Binding affinity of BIRC6^{UBC} variants to E1s or E1-Ub. a-f, Bio-layer interferometry (BLI) analysis of binding of BIRC6^{UBC} to UBA6 (**a**), UBA1 (**b**), UBA6-Ub (**c**) or UBA1-Ub (**d**) , and of the BIRC6$^{UBC\Delta insertion\ loop}$ variant to UBA6-Ub (**e**) and UBA1-Ub (**f**). **g**, Summary of dissociation constants derived from BLI assays. Binding affinities determined from two independent experiments.

2. Labeling strategy and control with a promiscuous E2

We switched the label to the smaller protein (BIRC6^{UBC}) and repeated the MST experiments. The results showed that BIRC6 still bound to both UBA1 and UBA6 with comparable affinity (1.3 μM vs 5.01 μM) (**Rev. Fig. 12c**), which is consistent with the binding affinities measured when the label was placed on the E1.

We next tested the binding of a cross-reactive E2 (UbcH5c) to UBA1 and UBA6 by MST. As expected, UbcH5c bound to both E1s with comparable affinity (**Rev. Fig. 12d**), confirming that our MST setup can detect physiologically relevant E1–E2 interactions.

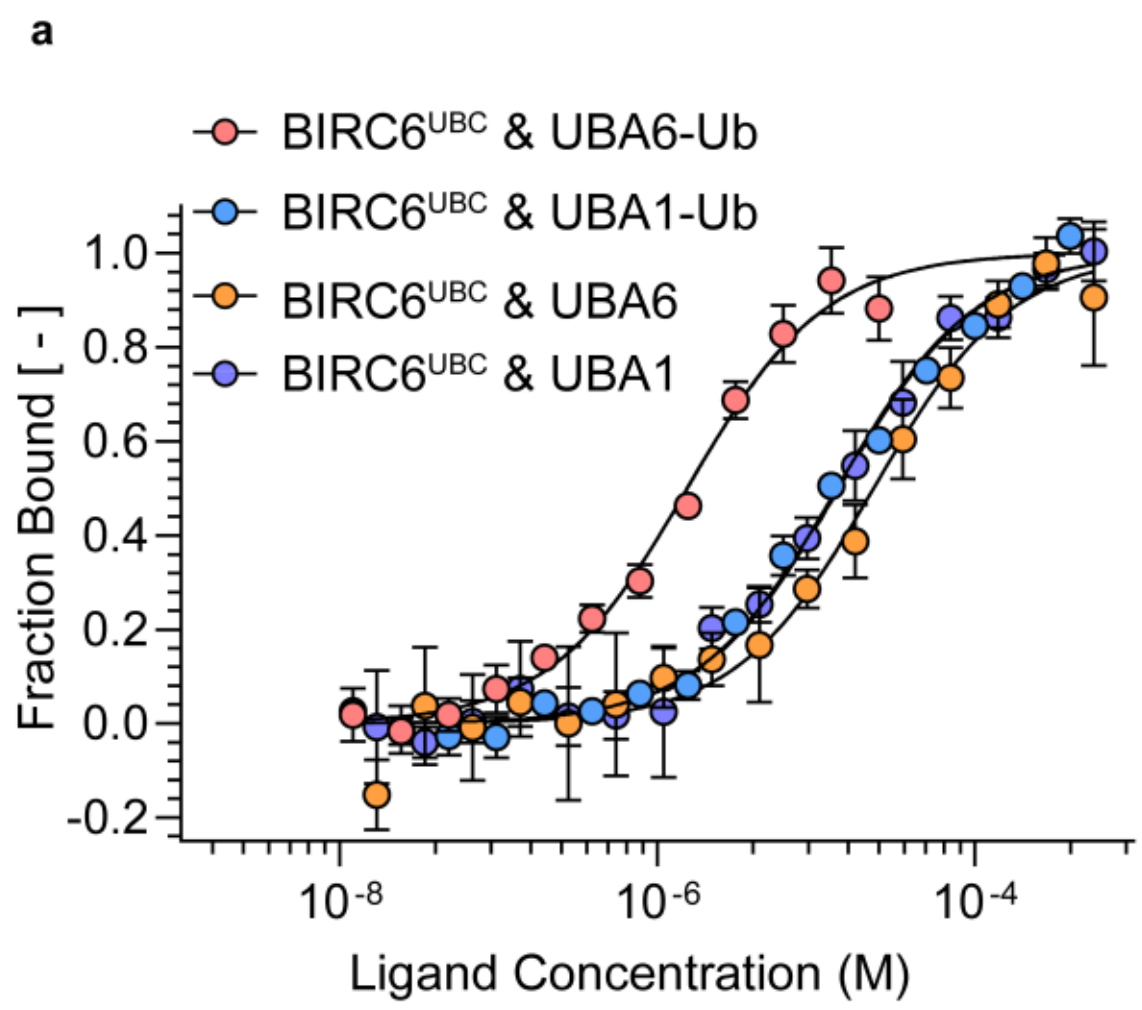

Group	BIRC6^{UBC}	E1	K_D(μM)	K_D confidence (μM)
1	WT	UBA6	23.02	±4.29
2	WT	UBA1	14.00	±1.66
3	WT	UBA6-Ub	1.53	±0.22
4	WT	UBA1-Ub	13.58	±1.6

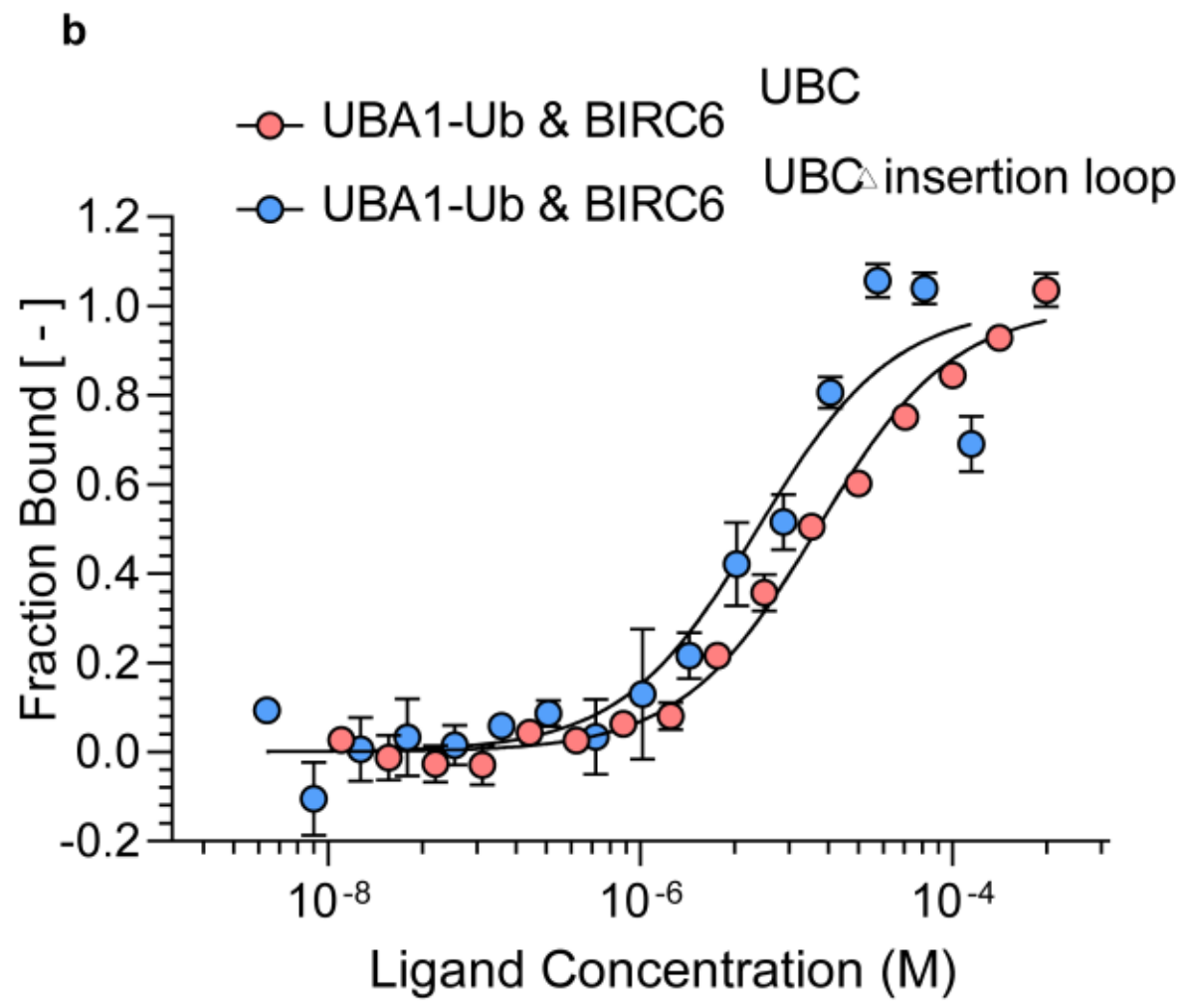

Group	BIRC6^{UBC}	E1	K_D(μM)	K_D confidence (μM)
1	WT	UBA1~Ub	13.58	±1.6
2	△insertion loop	UBA1~Ub	5.61	±2.11

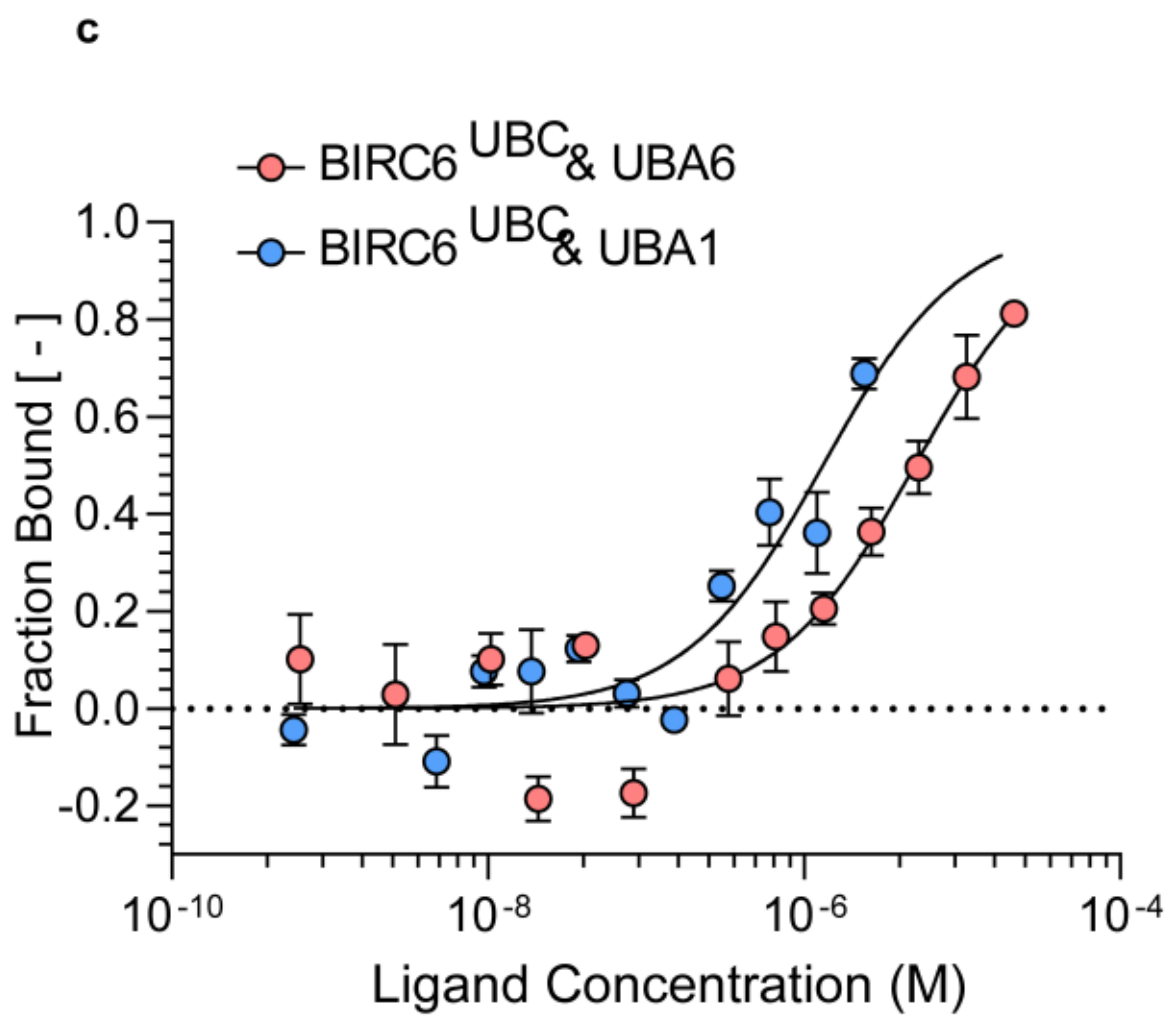

Group	BIRC6^{UBC}	E1	K_D(μM)	K_D confidence (μM)
1	WT	UBA6	5.01	±1.6
2	WT	UBA1	1.3	±2.11

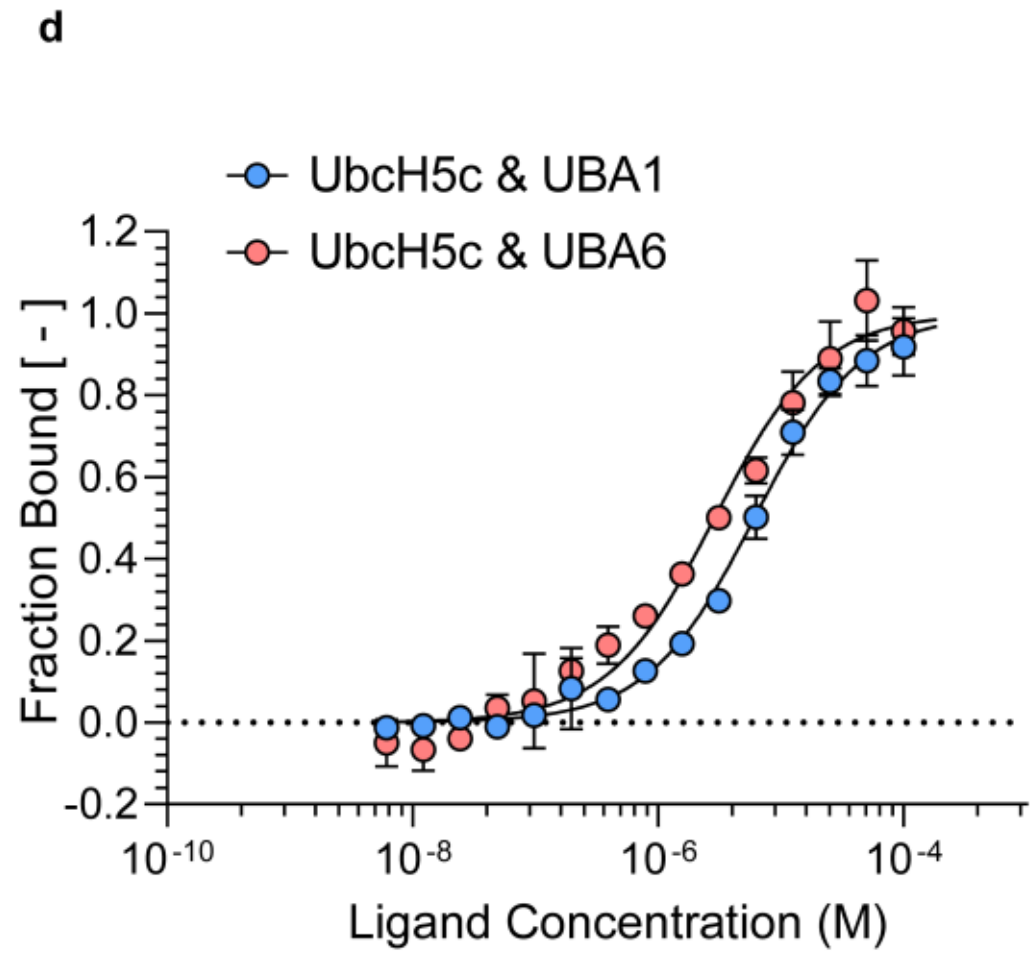

Group	E2	E1	K_D(μM)	K_D confidence (μM)
1	UbcH5c	UBA6	2.85	±0.56
2	UbcH5c	UBA1	6.04	±0.51

Rev. Fig. 12 | Microscale thermophoresis (MST) binding affinity data of BIRC6^{UBC} variants to E1s or E1-Ub. a, Microscale thermophoresis (MST) analysis of binding of BIRC6^{UBC} to E1 enzyme UBA6 or UBA1 and their ubiquitin-loaded forms. The fluorescent tracer in the MST assays was the labelled E1 protein or the corresponding E1-Ub species. **b**, MST analysis of binding of BIRC6^{UBC} or BIRC6$^{UBC\Delta insertion\ loop}$ to ubiquitin-loaded UBA1. The fluorescent was attached to the ubiquitin-loaded UBA1. **c-d**, MST analysis of binding of E2 enzyme BIRC6^{UBC} or Ubch5c to E1 enzyme UBA1 or UBA6. The E2 enzyme was fluorescently labelled. These data Data are presented as mean ± s.d. from technical triplicates. The summary of dissociation constants are shown below.

3. Reconciliation with published data

In the recent study by Riechmann et al. (*Nat. Struct. Mol. Biol.* **2026,** 33, 464–478; PMID

41350950), the authors used size-exclusion chromatography (SEC) to show that BIRC6^{UBC} co-elutes with UBA6 but not with UBA1, indicating that stable E1–BIRC6 complex formation occurs only with the cognate E1. Subsequently, they measured the binding affinity of BIRC6^{UBC} for UBA6 by fluorescence polarization (FP), reporting a K_D of approximately 0.2 μM. However, the binding affinity of BIRC6^{UBC} to UBA1 was not tested.

Our MST and BLI results both indicate that BIRC6^{UBC} binds UBA1 and UBA6 with similar affinity. We attribute this apparent discrepancy to the fact that the two experimental approaches probe different aspects of protein interactions. SEC reflects complex stability and hydrodynamic behavior (i.e., how long the complex persists and its overall shape), whereas MST and BLI measure binding affinity (i.e., how tightly the proteins interact at equilibrium). Thus, while MST and BLI data indicate similar apparent affinities of UBA1 and UBA6 for BIRC6, these measurements capture the binding energy at the initial interaction step. SEC, by contrast, reports on the stability of the complex during prolonged separation and may be influenced by conformational states that affect complex persistence. **The two sets of findings are not contradictory but rather provide complementary perspectives on the interaction.**

4. Effect of ubiquitin loaded E1 on binding

We further assessed the binding of BIRC6^{UBC} and BIRC6$^{UBC\Delta insertion\ loop}$ to UBA1~Ub and UBA6~Ub by both MST and BLI **(Rev. Fig. 11, Rev. Fig. 12a).** The results showed that when using oxyester-linked UBA1-Ub and UBA6-Ub conjugates to mimic the catalytically charged E1 state (where "-" denotes the oxyester linkage), BIRC6 binding to UBA6-Ub was significantly enhanced, with an approximately 9-fold increase by MST and a 2-fold increase by BLI, whereas binding to UBA1-Ub remained unchanged, whereas binding to UBA1-Ub remained unchanged. This indicates that under catalytically active conditions, BIRC6^{UBC} preferentially binds UBA6, consistent with the conclusion by Riechmann et al. that a stable interaction occurs only with its cognate E1, UBA6.

Moreover, truncation of the insertion loop in BIRC6^{UBC} markedly enhanced binding to UBA1-Ub, further supporting a critical role for the BIRC6 insertion loop in mediating UBA6 specificity (**Rev. Fig. 12b**).

5. Refined mechanistic model

Based on the combined experimental evidence, we have refined our mechanistic model (**Rev. Fig. 13**). BIRC6 preferentially binds its cognate E1, UBA6-Ub, over UBA1-Ub. This preference is driven, as reported by Paul and colleagues, by specific recognition of the UBA6 UFD domain. Our findings further establish that structural rearrangements of the gate helix within the UBA6 SCCH domain accommodate the insertion loop of the BIRC6^{UBC} domain, representing an additional key contributor of UBA6 specificity. This dual mechanism explains why swapping either the UFD or the SCCH domain between UBA1 and UBA6 alters BIRC6 specificity, and why combined interactions with both domains are required for BIRC6 activity.

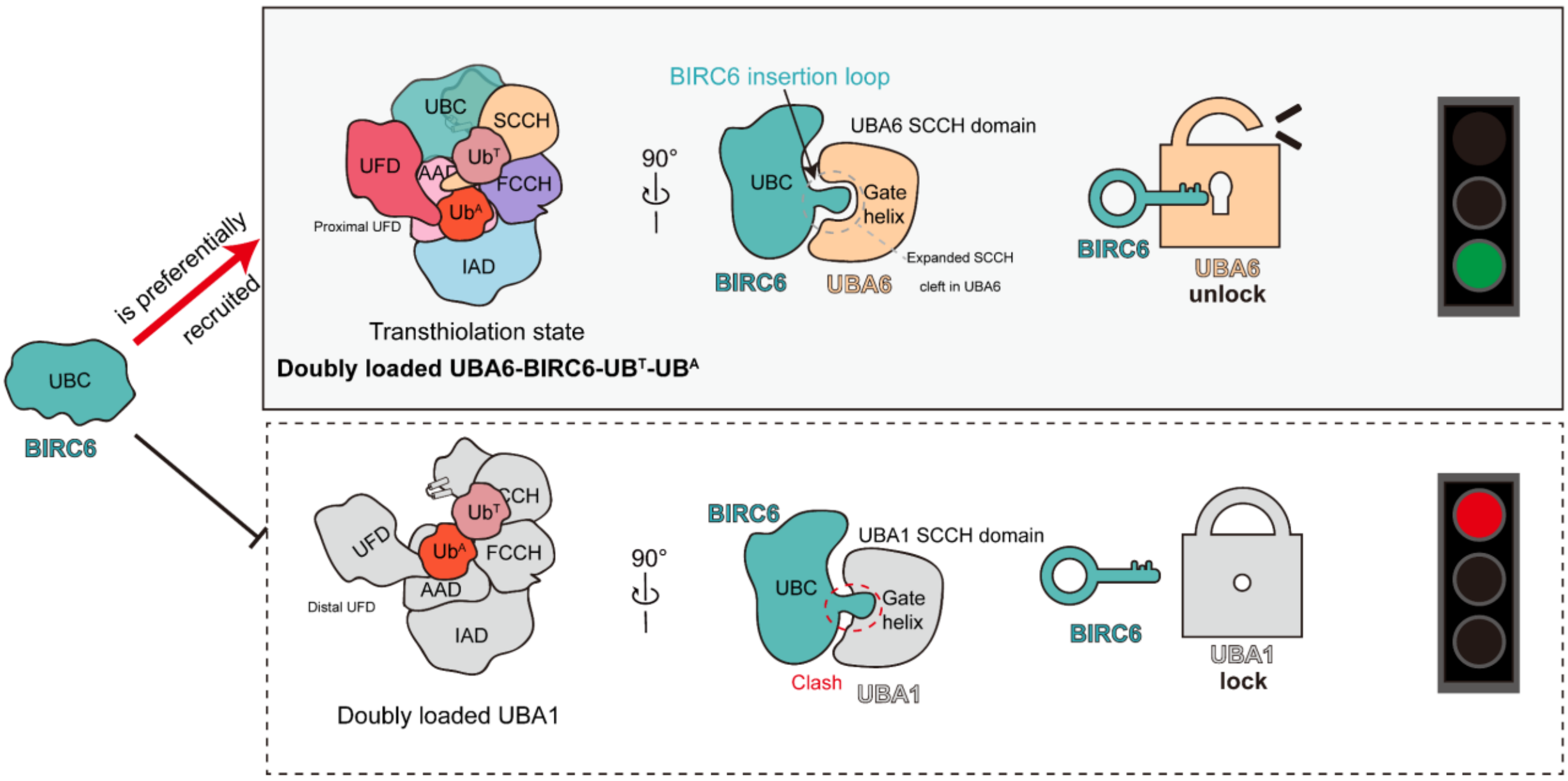

Rev. Fig. 13 | Model of BIRC6^{UBC} specificity with UBA6 over UBA1 in transthioesterification.
BIRC6 is preferentially recruited to UBA6~Ub rather than UBA1~Ub. In addition, the SCCH domain of UBA6 contains an expanded cleft, and the gate helix adopts a distinct conformation structurally configured to accommodate the insertion loop of BIRC6^{UBC}. The insertion loop fits into a compatible cavity created by the rotated gate helix, enabling catalysis (unlock). In contrast, UBA1 fails to catalyze transthioesterification due to steric clash between the insertion loop of BIRC6^{UBC} and the gate helix of UBA1, which prevents productive engagement (lock).

7. The authors attempt to further demonstrate binding of UBA1 to BIRC6 by means of a competition assay where UBA1 is titrated into a UBA6-BIRC6 reaction. Figure 7b is confusing in that there is no ubiquitin loaded on UBA6 in the absence of UBA1, which is strange. Can the authors clarify this?

Response: The absence of a visible UBA6~Ub band in the absence of UBA1 can be explained by the low UBA6 concentration used (0.1 µM), which produces a weak fluorescent signal. The band becomes detectable under high exposure (**Rev. Fig. 14a-b**).

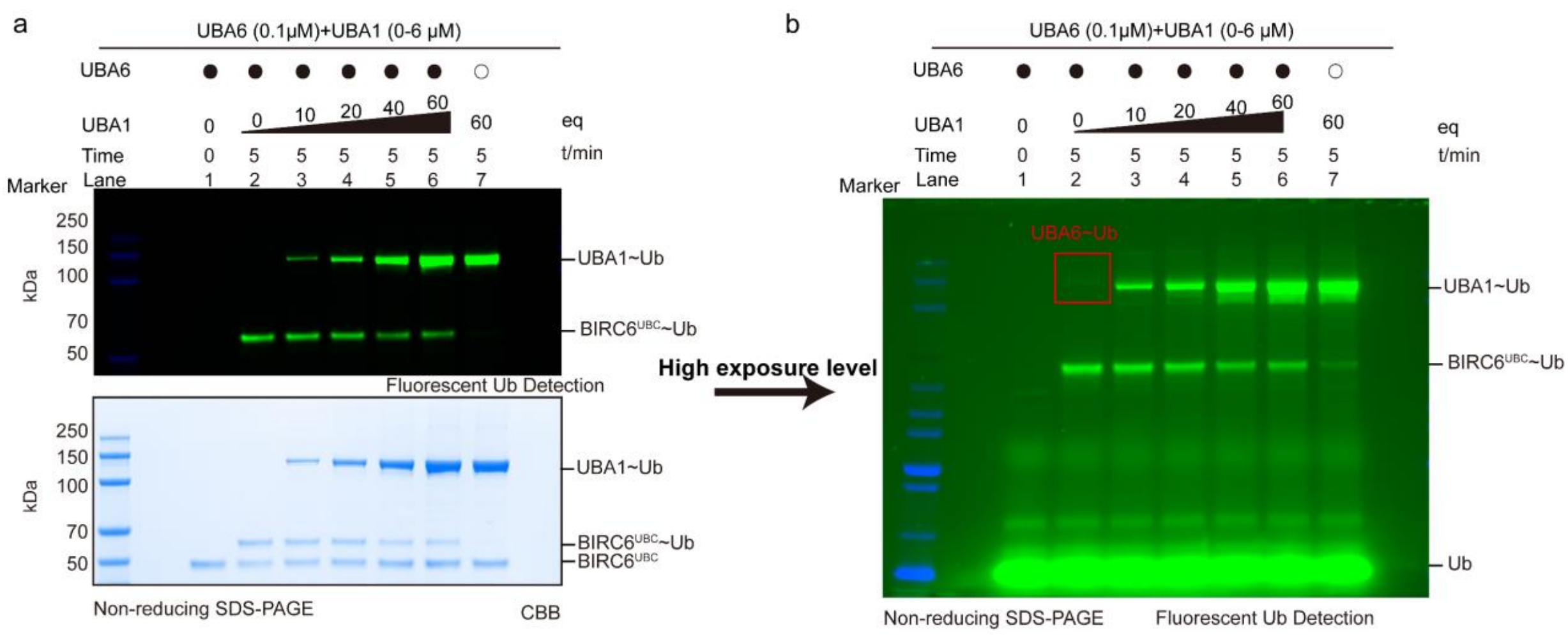

Rev. Fig. 14 | a-b, Fluorescent gel images corresponding to Fig. 5b at different exposure settings. a-b, Fluorescent gel images of Figure 7b under normal (a) and high (b) exposure conditions. A weak fluorescent UBA6~Ub signal is highlighted by a red box when 0.1 µM UBA6 concentration is used.

The authors should determine if UBA1 is outcompeting UBA6 loading of BIRC6 due to the consumption of ATP in the assay caused by increasing UBA1 concentrations. Does an inactive UBA1 (either adenylation incompetent, or thiolation inactive) also out-compete UBA6-dependent transfer onto BIRC6? Furthermore, if BIRC6 is binding to UBA1 in a catalytic incompetent state as suggested, an effect on UBA1-functioning E2s would be observed when BIRC6 is titrated into UBA1 reactions containing other E2s. This should be demonstrated by the authors.

Response: We thank the reviewer for raising this point. We expressed a UBA1 C/A mutant (active Cys623 mutated to Ala) and an ATP-binding-deficient mutant (D576A, K528E, R515A), and tested their effects on the UBA6–BIRC6^{UBC} transthioesterification reaction. The results showed that both mutants exhibited inhibition of BIRC6^{UBC}~Ub formation (**Rev. Fig. 15 a,b**). This indicates that the inhibition by WT UBA1 is not primarily mediated by competition for ATP or ubiquitin.

In addition, we performed reverse competition experiments by adding the catalytically inactive BIRC6^{UBC} C/A mutant to the UBA1–UbcH5c reaction. BIRC6^{UBC} C/A had no detectable effect on UbcH5c~Ub formation in the UBA1 system (**Rev. Fig. 15 c**). This observation may be explained by our MST binding data, which showed that UbcH5c binds UBA1 with higher affinity (6 μM) compared to BIRC6^{UBC} (13 μM) (**Rev. Fig. 12 d**). Furthermore, Riechmann et al. confirmed that BIRC6^{UBC} does not form a stable complex with UBA1 by size-exclusion chromatography. Together, these findings suggest that the relatively weak and transient interaction between BIRC6^{UBC} and UBA1 is insufficient to compete effectively with UbcH5c under the conditions tested.

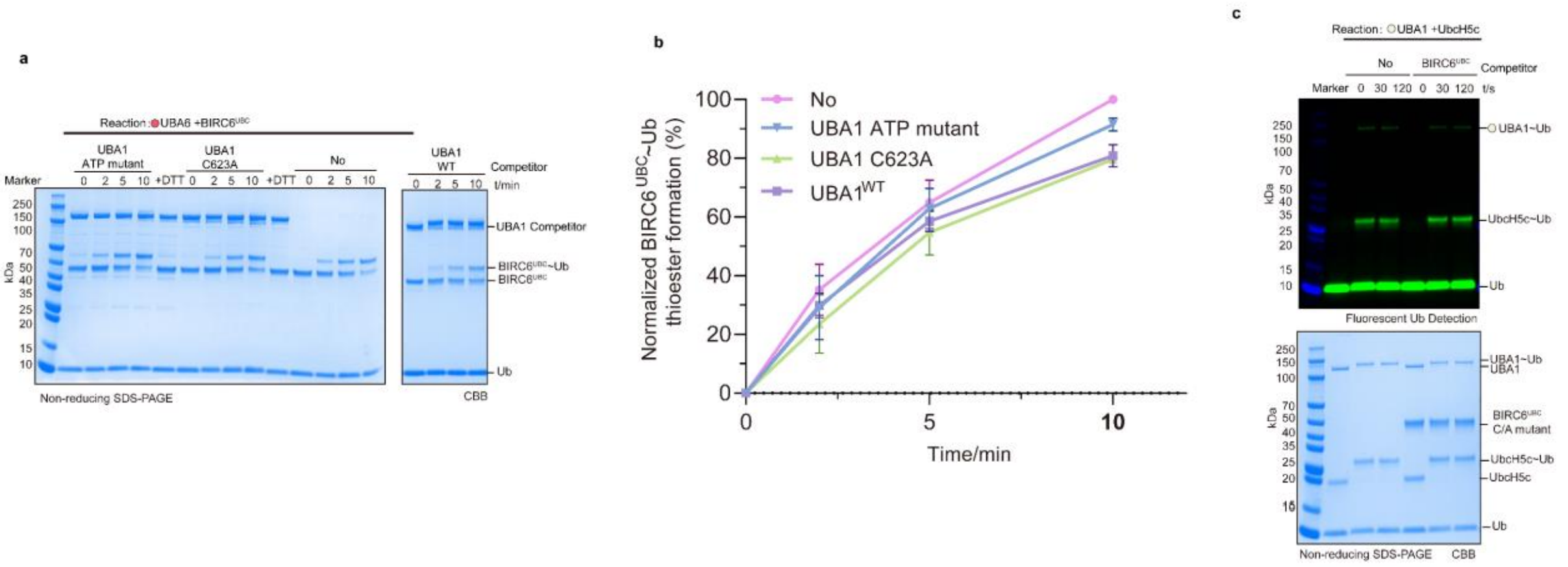

Rev. Fig. 15 | UBA1 variants compete with UBA6 in in BIRC6^{UBC} transthioesterification. a, Biochemical analysis of competition between UBA6 and UBA1 variants during BIRC6^{UBC} transthioesterification. UBA1 variants include UBA1 C623A mutant (active-site Cys623 mutated to Ala), UBA1 ATP mutant (ATP-binding-deficient, containing D576A, K528E, R515A mutations), and UBA1^{WT}. Reactions contained 0.1 μM UBA6 and 6 μM of UBA1^{WT}, UBA1 C623A, or UBA1 ATP mutant. Time points: 0, 2, 5, 10 min. **b,** Quantification of the BIRC6^{UBC}~Ub shown in (a). Data are presented as mean ± s.d. from two independent biological experiments. Quantification was performed on Coomassie Brilliant Blue-stained gels, and the percentage of BIRC6^{UBC}~Ub was calculated as the ratio of the charged form to total BIRC6^{UBC} (uncharged + charged). Relative activities were then obtained by normalizing to UBA1^{WT}. **c**, BIRC6^{UBC} C/A mutant has no inhibitory effect on UBA1-Ubch5c transthioeaterification. The gel image represents independent biological replicates (n=2). Time points: 0, 30, 120s.

8. Finally, the authors generate a model whereby the UFD domain from their BIRC6 cryoEM structure is docked onto the UFD domain of PDB ID 4NNJ to reveal a conformational difference of UFD-BIRC6 that prevents BIRC6 full engagement with UBA1. I find the rationale for this confusing since

in Extended Figure 8 there is little RMSD difference between the author's structure and reported E1-E2 structures, which are mostly UBA1-derived. What is the overall RMSD between the entire E1 of 4NNJ and the reported UBA6-BIRC6 structure? It is known that the UFD domain of E1s can adopt different conformations, so the rationale for taking an E1 not bound to an E2 and superimposing just the UFD domains seems flawed.

Response: We agree with the reviewer's assessment. Based on our most recent experimental data and the refined model, in which BIRC6 exhibits clear preferential binding to UBA6~Ub, directly docking BIRC6 into the apo UBA1 structure (PDB 4NNJ) is no longer justified. Therefore, we have removed this structural docking analysis from the manuscript.

Other points

Line 15. BIRC6 is not specifically activated by UBA6 as written by the authors. E2s are not activated by E1s. There are several instances throughout the text where this is referred to.

Response: We thank the reviewer for this correction. We agree that E2s are not "activated" by E1s; rather, Ubs is activated by E1s, and E1s catalyze E2~Ub thioester formation. We have revised the text accordingly in the Results, Discussion, and Methods sections, replacing "activated" with more accurate terminology such as "*E1 charge E2*" or "*E1 engage E2*"

Lines 59 and 62. It is not clear what the apoptotic pathway being referred to is. In addition, reference 31 describes the role of BIRC6 in regulating autophagy, not programmed cell death.

Response: We thank the reviewer for pointing this out. In the Introduction, we have added a more detailed description of the specific step in the apoptotic pathway in which BIRC6 is involved, as shown below:

"BIRC6 is a giant 4857-amino acid E2–E3 hybrid enzyme and the only essential inhibitor of apoptosis (IAP). Apoptosis is executed by a family of cysteine proteases known as caspases, which initiate and carry out programmed cell death in response to developmental cues or cellular stress. IAP proteins function by directly restricting these caspases, thereby inhibiting the apoptosis. BIRC6's C-terminus contains a ubiquitin-conjugating (UBC) domain, which is a hallmark of the E2 ubiquitin-conjugating enzyme family, while its N-terminal baculoviral IAP repeat (BIR) domains recognize specific degrons on substrates, conferring E3 ligase-like activity for direct substrate recruitment. In the apoptosis pathway, BIRC6 functions exclusively with UBA6, but not UBA1,enabling it to ubiquitinate substrates such as caspases-3, -7, -9 and HTRA2 , thereby suppressing apoptosis."

Additionally, reference 31 has been removed.

Line 95. This sentence requires citation of work demonstrating a double-loaded E1 is required for maximum ubiquitin transfer onto an E2.

Response: We thank the reviewer for the suggestion. We have added the following citation to support the statement that a double-loaded E1 is required for maximum ubiquitin transfer onto an E2:

"Pickart, C.M., Kasperek, E.M., Beal, R. & Kim, A. Substrate properties of site-specific mutant ubiquitin protein (G76A) reveal unexpected mechanistic features of ubiquitin-activating enzyme (E1).

J. Biol. Chem. 269, 7115-7123 (1994)."

Line 102. It is unclear what the authors mean by the atomic-level precision of protein chemical synthesis and the associated citations 36-41 are irrelevant to this manuscript.

Response: We thank the reviewer for pointing this out. We have replaced this sentence with a more appropriate statement regarding the role of ubiquitin-based probes in structural studies of the E1-E2-E3 cascade, supported by relevant references. The revised text is shown below:

"*Activity-based ubiquitin probes have significantly advanced structural studies of ubiquitin-related enzymes, including the ubiquitin E1-E2-E3 cascade and deubiquitinating enzymes (DUBs).*"

Line 142. The authors should acknowledge the Lima lab first used focused 3D classification for resolving ubiquitin transfer states observed when using the PSAN probe approach for capturing ubiquitin transfer from UBA1 to Ubc4 (PMID: 39143218).

Response: We thank the reviewer for this important point. We have now acknowledged that the Lima lab first employed focused 3D classification to resolve ubiquitin transfer states using the PSAN probe approach for capturing ubiquitin transfer from UBA1 to Ubc4 ((*Nature*, **2024**, 633, 216–223, PMID: 39143218). This has been added to the relevant section of the manuscript with the appropriate citation.

"We therefore performed focused 3D classification, an approach first applied to capture ubiquitin transfer states by Kochańczyk and co-workers in the context of UBA1-Ubc4 using the PSAN probe. For both *the singly loaded UBA6-BIRC6^{UBC}-Ub^{T} and doubly loaded UBA6-BIRC6^{UBC}-Ub^{T}-Ub^{A} complexes, focused 3D classification yielded 10 reconstructions each, revealing pronounced Ub^{T} heterogeneity (**Extended Fig. 3d, e**). These reconstructions capture a conformational continuum—from Ub^{T} positioned near the FCCH domain of UBA6 to states proximal to BIRC6^{UBC}. This spatial progression implies the process of the Ub^{T} transfer from UBA6 catalytic cysteine to BIRC6^{UBC} (**Extended Fig. 3d, e**)."*

Line 218. In the interests of transparency, the authors should highlight that the gate helix E1 chimeras have lower thiolation activity (Extended Figure 8). In addition, the authors should demonstrate that transfer of ubiquitin on UBA1/6 dual active E2s is not impaired.

Response: To improve the transparency of our work, we performed E1~Ub charging assays and E1–UbcH5c transthioesterification assays to comprehensively evaluate the activities of UBA1, UBA6, and the gate helix E1 chimeras. A detailed description of the reduced thiolation activity of the gate helix chimeras has now been added to the Results section. The revised text is as follows:

"*We next engineered chimeric E1 enzymes by exchanging the gate helix. Specifically, we generated UBA6, UBA1, a chimera in which UBA6's gate helix was replaced with that of UBA1 (referred to as UBA6$^{GH\text{-}UBA1*}$), and a chimera in which UBA1's gate helix was replaced with that of UBA6 (referred to as UBA1$^{GH\text{-}UBA6*}$). E1~Ub charging assays showed that UBA6$^{GH\text{-}UBA1*}$ exhibited little to no ability to activate ubiquitin, whereas UBA1$^{GH\text{-}UBA6*}$ retained approximately 50% of the activity compared to UBA1^{WT} (**Extended Fig. 8f**). Consistent with this, transthioesterification assays using the promiscuous E2 UbcH5c revealed that UBA6$^{GH\text{-}UBA1*}$ failed to catalyze UbcH5c~Ub formation, while UBA1$^{GH\text{-}UBA6*}$ remained capable of charging UbcH5c, albeit with reduced efficiency (**Extended Fig. 8g, h**). Because the UBA6$^{GH\text{-}UBA1*}$ chimera is defective in the ubiquitin transfer*

*cascade, it precluded further transthioesterification assays with the BIRC6^{UBC}. Therefore, we tested only UBA1$^{GH\text{-}UBA6*}$ for its transthioesterification activity toward BIRC6^{UBC}. A time-course quantitative comparison of the activities of UBA1^{WT}, UBA6^{WT}, and UBA1$^{GH\text{-}UBA6*}$ toward BIRC6^{UBC} showed that the UBA1$^{GH\text{-}UBA6*}$ chimera partially restored this activity, whereas UBA1^{WT} showed no detectable charging of BIRC6^{UBC} (**Fig. 4h, Extended Fig. 8i**).”*

The methods section outlines differences in reaction conditions used for the transthiolation assays. This should be clearly stated in figure legends e.g. Figures 1, 3 & 4 need to highlight the different lengths of time the assays were performed for. i.e. structural activities probed after 2minutes, whereas specificity switch mutations were visualised after a 30-minute reaction.

Response: We thank the reviewer for this important suggestion. In the revised manuscript, we have clearly indicated the different reaction conditions in the corresponding figure legends. Specifically, for structural mutant assays (**Fig. 3h**), we now specify that the reactions were performed for 2 minutes. For specificity gate helix switch mutants (**Fig. 4h, i**), we performed time-course experiments (0, 2, 5, 10, and 30 min) to capture the dynamic range of activity, and this has been noted in the relevant figure legends. These details have also been added to the Methods section to ensure transparency and reproducibility.

Reviewer #1 (Remarks to the Author):

The authors addressed all my concerns, and I support publication in Nature Communications.

Response: We thank the reviewer for supporting the publication of our manuscript.

Reviewer #2 (Remarks to the Author):

The authors have made a good attempt to respond to both reviewers' comments. However, I continue to have concerns about the interpretation of the affinity measurements that the authors use to underpin their conclusions.

1. The authors have now included a second technique (BLI) to measure E1-BIRC6 affinity that gives very different affinities. While I appreciate that differences in affinities can arise between orthogonal techniques, I disagree with the authors' suggestion that a 3-10 fold difference in affinities is still acceptable. Notably in their revised BLI data the difference for BIRC6 binding to UBA6 is almost 17-fold different between MST and BLI (23 µM versus 1.3 µM). Equally concerning is the reciprocal experiment performed by the authors in Rev. Fig 12c, d (incidentally which is not included in the manuscript but only in the rebuttal) where the authors claim to observe a Kd of 5.6 µM when the label is on BIRC6 UBC. It is not clear how the authors could determine a Kd from this fit since at the concentration range used the fraction bound does not reach 1 and the quality of the data points is noisy.

Response: We thank the reviewer for raising these thoughtful concerns regarding the discrepancy in affinity values obtained from MST and BLI assays, as well as the quality of previous fitting results. We have thoroughly re-evaluated all binding experiments in response to your comments, and identified **non-specific binding artifacts** that interfered with our original MST and BLI measurements.

1. Identification of non-specific binding in the initial assays

We consulted technical specialists from the manufacturers of MST and BLI instruments, and performed a series of blank control experiments to assess off-target interactions. For MST assays, control tests confirmed that $BIRC6^{UBC}$ could bind to free Red-tris-NTA dye in the absence of E1 proteins, with an apparent K_D of 975 nM (**Rev. Fig. 1a**). For the original BLI assays using Ni-NTA biosensors, a distinct binding signal was also detected on blank Ni-NTA sensors when incubated with $BIRC6^{UBC}$ at the highest tested concentration (3240 nM) (**Rev. Fig. 1b**). These non-specific interactions were the main cause of the inconsistent affinity values between two orthogonal techniques and the poor quality of previous fitting curves.

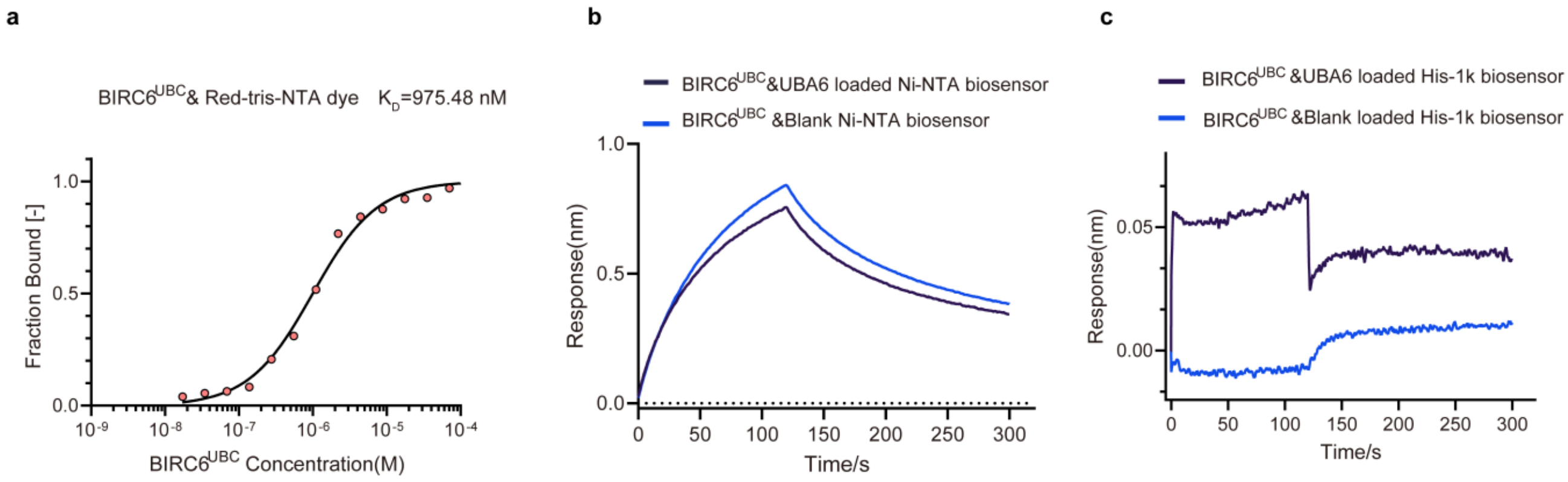

Rev. Fig. 1 | MST analysis of BIRC6^{UBC} binding to free Red-tris-NTA dye and BLI analysis of BIRC6^{UBC} binding to blank Ni-NTA sensors. a, MST binding analysis of BIRC6^{UBC} to blank Red-tris-NTA dye. Fitted K_D =975.48 nM. **b,** BLI binding analysis of BIRC6^{UBC} to blank and UBA6-loaded Ni-NTA biosensor. At 3240 nM BIRC6^{UBC}, binding and dissociation signals on blank Ni-NTA biosensor were comparable to those on UBA6-loaded Ni-NTA biosensor. **c,** BLI binding analysis of BIRC6^{UBC} to blank and UBA6-loaded His-1K biosensor.

We first attempted to reduce non-specific adsorption by serially increasing imidazole concentrations in the running buffer (10 mM, 30 mM and 50 mM). However, this approach failed to eliminate the non-specific binding of BIRC6^{UBC}, and also weakened the specific interaction between His-tagged proteins and Ni-NTA biosensors. According to official manufacturer guidelines (*Harmonizing Regulatory Guidelines for Assay Validation: A Focus on Kinetics-Based Assays Using Octet® BLI Systems*), conventional Ni-NTA biosensors capture His-tagged proteins via metal chelation, a mechanism that is prone to non-specific adsorption for certain protein samples. To resolve this issue fundamentally, we replaced Ni-NTA sensors with high-specificity Anti-Penta-HIS (His1K) biosensors, which rely on specific antigen-antibody recognition toward His-tags and effectively eliminate non-specific binding.

2. Re-measurement of binding affinity using His1K biosensors

Following the guidance of BLI technical specialists, we repeated all BLI binding assays with His1K antibody biosensors. First, we performed blank sensor control using the maximum concentration of BIRC6^{UBC} (3240 nM). No observable association or dissociation signal was detected on blank His1K sensors, demonstrating that BIRC6^{UBC} does not bind to the sensor surface non-specifically within the tested concentration range (40 nM to 3240 nM) (**Rev. Fig. 1c**).

We next characterized the binding of BIRC6^{UBC} to different E1 or E1-Ub conjugates:

- **Cognate pairing (UBA6 and UBA6-Ub):** BIRC6^{UBC} binds UBA6 and UBA6-Ub with steady-state K_D values of 4.82 µM and 2.41 µM, respectively (**Rev. Fig. 2a, c, f**).
- **Non-cognate pairing (UBA1 and UBA1-Ub)**: In contrast to UBA6 and UBA6-Ub, which gave clear concentration-dependent responses and allowed robust steady-state fitting (**Rev. Fig. 2a, c, f**), the sensorgrams for UBA1 and UBA1-Ub largely overlapped across the 40–1080 nM range， with only a modest signal increase observed at 3240 nM (**Rev. Fig. 2b, d, f**). Further elevation of BIRC6^{UBC} concentration (up to 9720 nM) introduced excessive background noise and could not improve data quality. Consequently, the datasets lacked the necessary concentration-dependent variation for reliable steady-state affinity fitting.

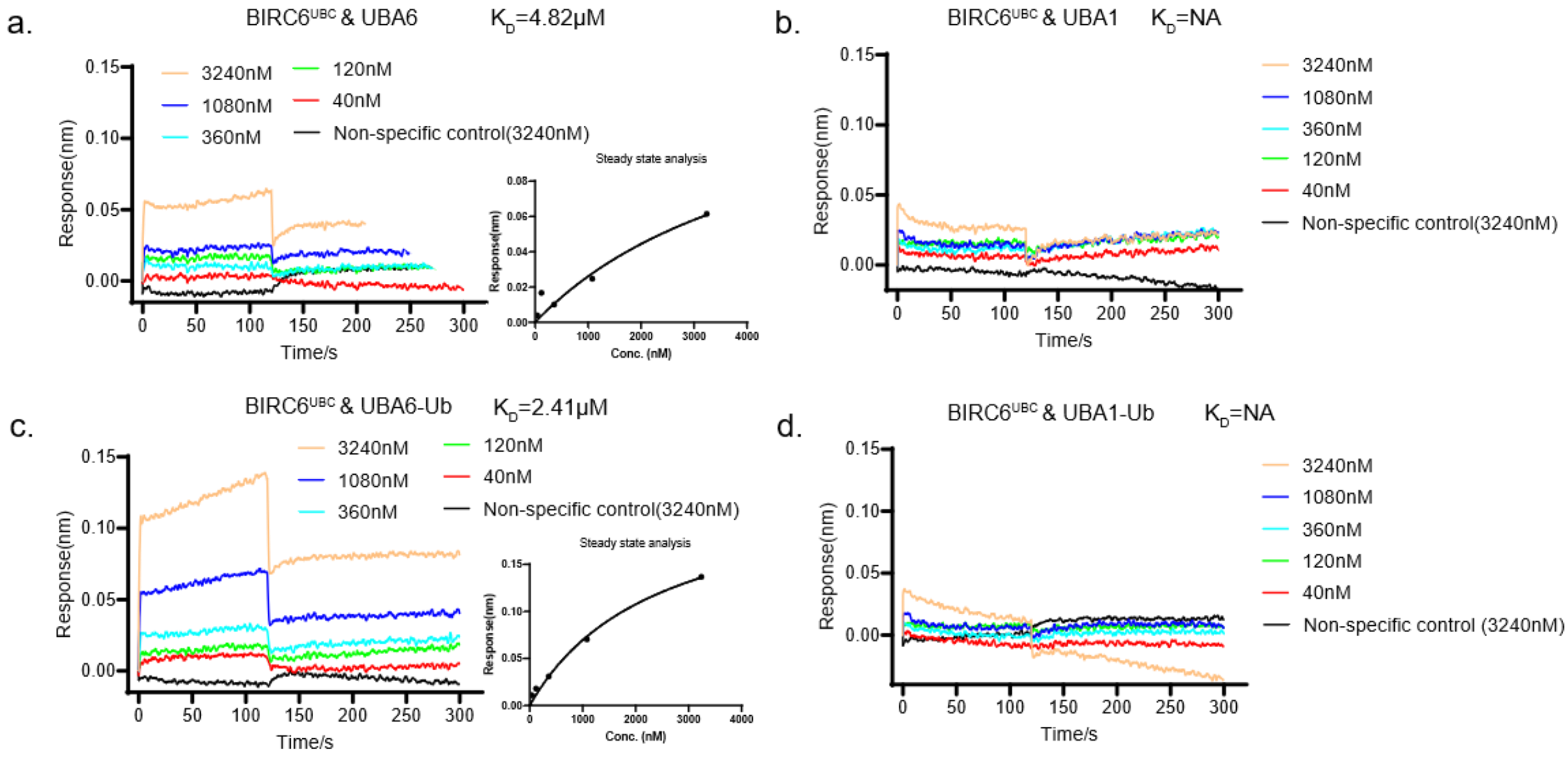

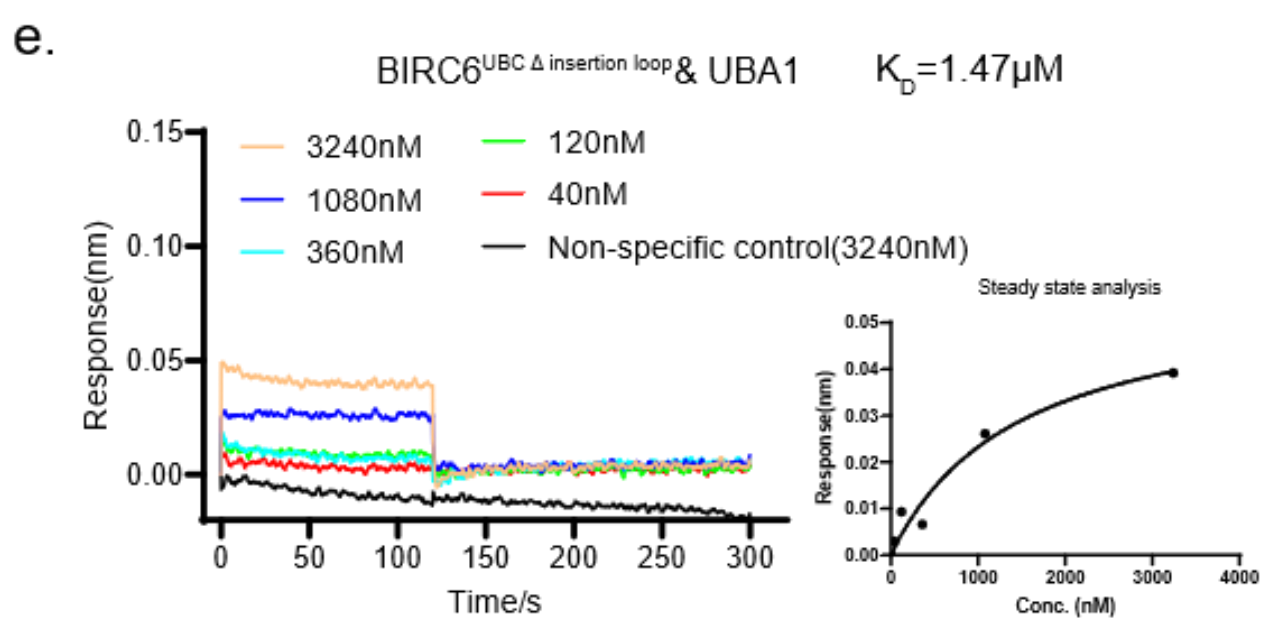

f.

	K_D(μM)	R^2
BIRC6^{UBC} & UBA6	4.82	0.91
BIRC6^{UBC} & UBA6-Ub	2.41	0.99
BIRC6^{UBC} & UBA1	NA	NA
BIRC6^{UBC} & UBA1-Ub	NA	NA
BIRC6$^{UBC\ \Delta\ insertion\ loop}$ & UBA1	1.47	0.94

Rev. Fig. 2 | BLI binding measurements of BIRC6^{UBC} and variants to E1 and Ub-loaded E1 using His-1K biosensor. a-e, BLI analysis of binding of BIRC6^{UBC} to UBA6 (**a**), UBA6-Ub (**c**), or UBA1 (**b**), UBA1-Ub (**d**) and of BIRC6$^{UBC\Delta insertion\ loop}$ to UBA1 (**e**). Concentrations of BIRC6^{UBC} and BIRC6$^{UBC\Delta insertion\ loop}$ used were 40, 120, 360, 1080, and 3240 nM. Right panels in (a), (c), and (e) show binding curves fitted by steady-state analysis. NA = Not available; binding curve could not be obtained by steady-state analysis within the tested concentration range. **f**. Summary of K_D values derived from BLI assays.

These results reconcile our findings with the size-exclusion chromatography (SEC) co-elution profiles reported by Riechmann et al. (*Nat. Struct. Mol. Biol.* **2026**, 33, 464), verifying that BIRC6^{UBC} preferentially interacts with UBA6 rather than UBA1.

Crucially, the BIRC6^{UBC} variant with a truncated insertion loop (BIRC6$^{UBC\triangle insertion\ loop}$) regained detectable binding to UBA1, with a steady-state K_D of 1.47 μM (**Rev. Fig. 2e**). This gain-of-binding phenotype is consistent with our in vitro transthioesterification assays, in which deletion of the insertion loop rescued UBA1-mediated ubiquitin charging of BIRC6^{UBC} (**main Figure 4i**). Together with the above binding data, these observations support our model that the BIRC6^{UBC} insertion loop and the UBA1 gate helix are key determinants in excluding UBA1 while enabling UBA6 specificity. And all original binding data acquired from MST and Ni-NTA-based BLI assays have been replaced with the corrected BLI results obtained using His1K biosensors. We appreciate your critical comments, which prompted us to identify technical flaws in the initial assays and substantially improved the robustness and reliability of our present work.

2. The authors now record an affinity between BIRC6 and Ub-loaded E1. Once again there is a discrepancy between the two techniques. The increase in affinity for BIRC6 to the UBA6-Ub loaded state observed by MST (15 fold increase) is interesting. However, by BLI, the increase observed is less significant (approximately 1.6 fold). The authors conclude that this makes UBA6 the preferred E1. Given the authors stated that a 3-10 fold difference in affinities is acceptable for concluding a comparable affinity, how can the authors conclude that a 1.6 fold difference by BLI is significant? Furthermore, can the authors explain mechanistically how a Ub-loaded UBA6 would bind better to UBA6 than a Ub-loaded UBA1?

Response: We apologize for the inconsistent affinity values obtained from the original MST and BLI measurements. As elaborated in the previous section, these datasets are unreliable due to non-specific interactions between BIRC6^{UBC} and the Red-tris-NTA dye (for MST) as well as Ni-NTA biosensors (for BLI). We therefore base all our interpretations exclusively on the updated BLI results acquired with high-specificity His1K biosensors

In the revised assays, robust and fully saturable binding was observed between BIRC6^{UBC} and UBA6-Ub, with a steady-state K_D of 2.41 μM (**Rev. Fig. 2c**). In contrast, no valid binding curves could be generated for the interaction between BIRC6^{UBC} and UBA1-Ub under the same experimental concentration range (**Rev. Fig. 2d**). These observations indicate that BIRC6^{UBC} preferentially binds UBA6-Ub, while its interaction with UBA1-Ub is negligible.

3. Related to 1) and 2) I am not sure the authors should be so confident on the significance of specific interactions given the large differences between affinity measurements between MST and BLI? The authors note a 1.5-fold difference in binding of BIRC6 UBC lacking the insertion loop to UBA1, compared to wild-type BIRC6, but this effect is very modest compared to the difference of BIRC6 binding to UBA6-Ub, hence it is likely not affinity-driven differences. A more relevant interpretation would be the SCCH domain positions BIRC6 into a correct orientation for transthioesterification to occur, and the clash between the BIRC6 insertion loop and the UBA1 SCCH prevents proper orientation.

Response: As addressed in the above sections, the discrepancies between original MST and BLI data were due to non-specific binding artifacts, and we now interpret results based on the optimized BLI measurements. We concur with the reviewer's insightful interpretation that sterically compatibility and proper molecular orientation controlled by the SCCH domain play dominant roles during the catalytic process. Steric clashes between the BIRC6^{UBC} insertion loop and the SCCH domain of UBA1 further prevent the formation of a catalytically competent complex.

Guided by this perspective and combining our results with previous work from Riechmann et al. (*Nat. Struct. Mol. Biol.* **2026**, 33, 464) and Nayak et al. (*Nat. Commun.* **2026**, 17, 3302), we have refined our mechanistic hypothesis into a "Dual-Code" model to explain the molecular basis of UBA6–BIRC6 selectivity:

- **UFD Code:** The UFD domain acts as the primary binding interface and governs the initial partner selection of BIRC6, as demonstrated in the study by Riechmann et al.

- **SCCH Gate Helix Code:** Our data suggest that the SCCH domain functions as a "catalytic gatekeeper". The unique conformation of the UBA6 gate helix forms a sterically compatible pocket to accommodate the insertion loop of BIRC6^{UBC}. This structural alignment positions the

E2 active site appropriately relative to the E1~Ub thioester intermediate, which enables efficient transthioesterification (the "unlock" state). In contrast, steric hindrance between the UBA1 gate helix and the BIRC6^{UBC} insertion loop blocks the UBC domain from adopting the orientation required for catalysis (the "lock" state).

We have updated the Discussion section and the summary model (**Figure 5**) in the revised manuscript to incorporate this refined framework. This revised model provides a more comprehensive explanation for the molecular mechanism underlying the preferential interaction between UBA6 and BIRC6.

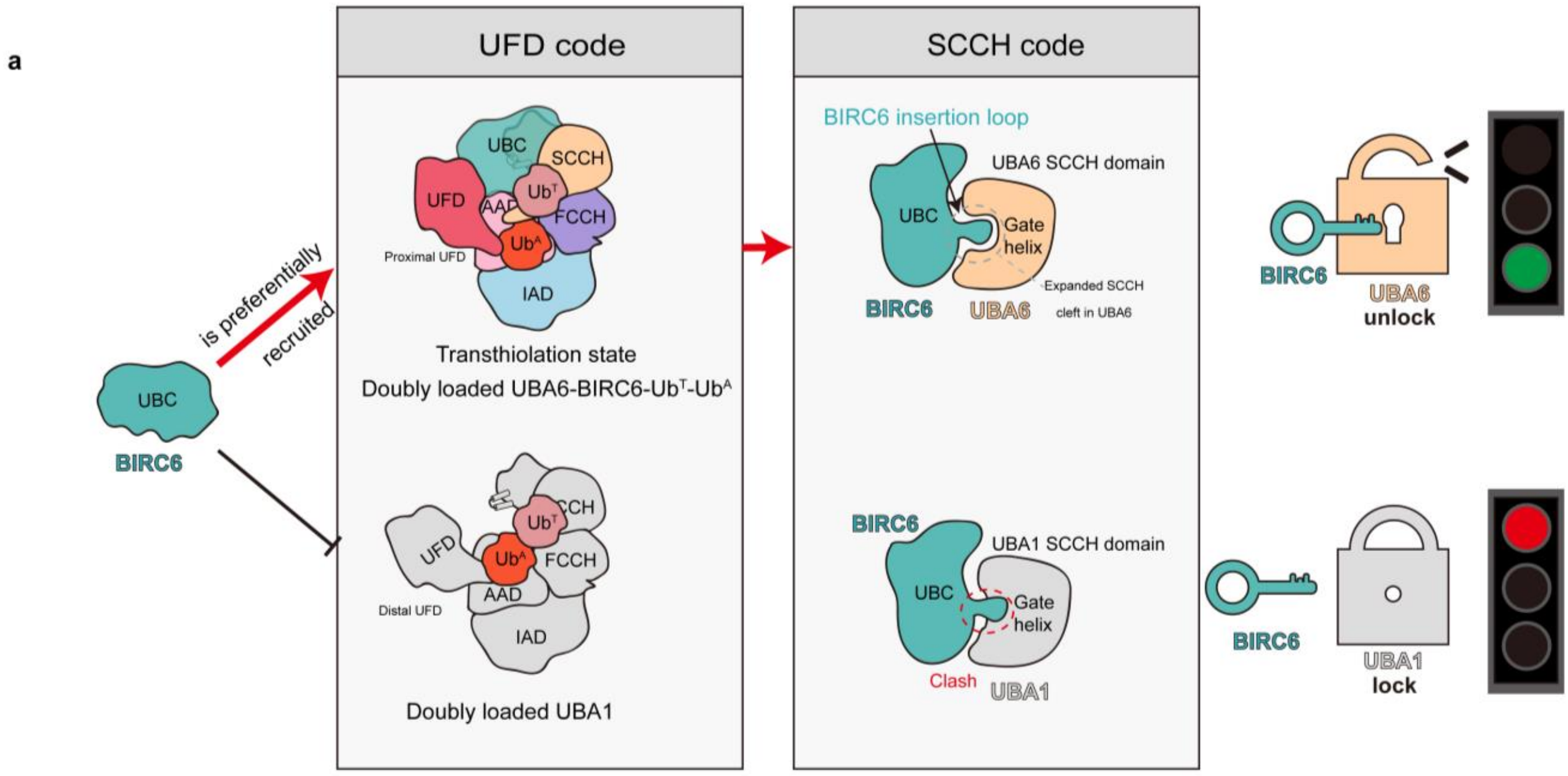

Rev. Fig. 3 | Dual code model of BIRC6^{UBC} specificity with UBA6 over UBA1 in transthioesterification. 1. UFD Code. BIRC6 is preferentially recruited to UBA6~Ub rather than UBA1~Ub. 2. SCCH Gate helix Code. The SCCH domain of UBA6 contains an expanded cleft, and the gate helix adopts a distinct conformation structurally configured to accommodate the insertion loop of BIRC6^{UBC}. The insertion loop fits into a compatible cavity created by the rotated gate helix, enabling catalysis (unlock). In contrast, UBA1 fails to catalyze transthioesterification due to steric clash between the insertion loop of BIRC6^{UBC} and the gate helix of UBA1, which prevents productive engagement (lock).

4. The authors argue that the differences in binding between UBA1 and UBA6 of BIRC6 by Riechmann et al is due to the different techniques used. I agree with this point but still do not understand how a complex of ~1 μM Kd with similar kinetic properties with regards to on and off rates as observed in BLI (Figure 5c,d) can still not bind in analytical SEC observed by Riechmann et al. The authors should not state that this complements the Riechmann et al. study because there is still a discrepancy between the two.

Response: We appreciate the reviewer's prompt for greater precision. We have refined our interpretation to provide a more accurate alignment with previous work.

1. Quantitative Alignment with SEC Findings

Our corrected BLI data demonstrate that BIRC6^{UBC} possesses measurable micromolar affinity for UBA6 (K_D = 4.82 μM) and UBA6-Ub (K_D = 2.41 μM), whereas binding to UBA1 remains

negligible and below the detection threshold (**Rev. Fig. 2a, c**). This clear distinction provides the biophysical basis for the SEC observations by Riechmann et al., where complex co-elution was observed exclusively for the UBA6–BIRC6 pair.

2. Refinement of Comparative Terminology

We acknowledge that the term “complements” was not appropriate for describing the relationship between the two studies. We have therefore replaced this expression in the revised manuscript. Instead of claiming full consistency, we now state that the two studies offer distinct yet synergistic mechanistic insights into the specificity of UBA6–BIRC6 interaction.

While Riechmann et al. elucidated the role of the UFD domain in initial partner selection, our study identifies the gate helix of UBA6 SCCH domain as a secondary checkpoint for catalytic compatibility. This revised wording acknowledges the unique contribution of each study while maintaining a cautious and accurate description of their relationship.

5. The molecular basis for specificity between BIRC6 and UBA6 is described as the complementary interactions between the gate helix of UBA6 and an insertion loop within BIRC6. The authors now show improved biochemistry in order to validate this model but it should be noted that the effects are moderate as while removing the insertion loop from BIRC6 has the strongest effect towards UBA1 activity, albeit with reduced kinetic effects (Figure 4i, Extended Figure 8j), the reciprocal experiment of replacing the gate helix in UBA1 with that of UBA6 only restored activity to 25% with BIRC6 (Figure 4h, Extended Figure 8i). Given that other papers (Riechmann et al and Nayak et al) have shown the contribution of both the SCCH and UFD for driving BIRC6 and UBE2Z specificity, is it likely that including this mutation in a UBA6-swapped UFD domain would further enhance activity towards BIRC6?

Response: We thank the reviewer for this thorough analysis of our mutagenesis data and thoughtful questions.

First, we would like to clarify the partial activity recovery of the $UBA1^{GH-UBA6*}$ chimera. Although this chimera only rescued approximately 25% of $BIRC6^{UBC}$ charging activity, its intrinsic catalytic activity as an E1 enzyme was already reduced to roughly 50% of wild-type UBA1 (**Rev. Fig. 4a-d**). In this context, the detectable gain-of-function still indicates that the UBA6 gate helix contributes to forming a catalytically compatible environment.

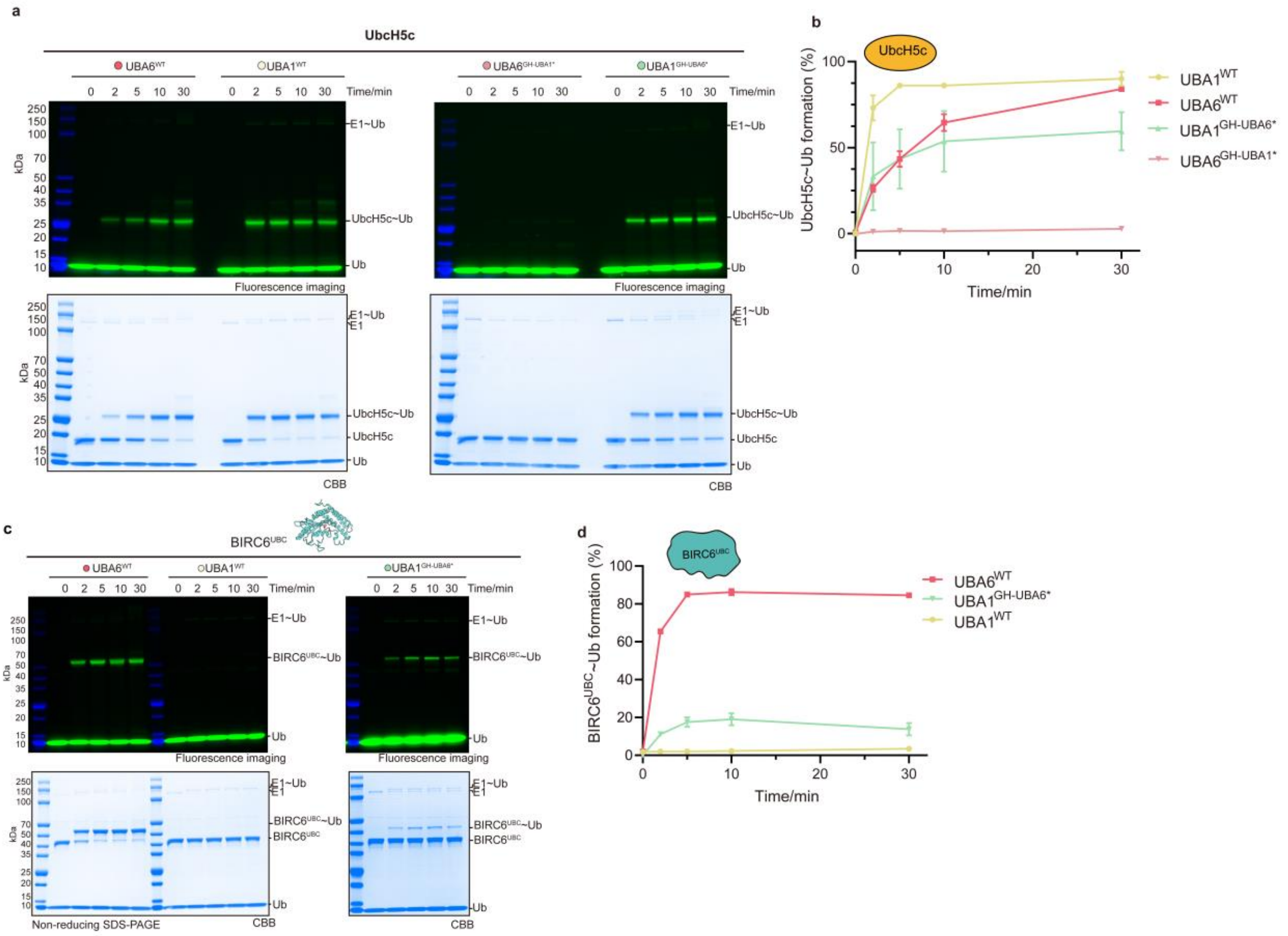

Rev. Fig. 4 | Enzymatic activities of E1 chimeras and BIRC6^{UBC} variants. a, E1-E2 transthioesterification assays using UBA6^{WT}, UBA1^{WT}, UBA6$^{GH-UBA1*}$ or UBA1$^{GH-UBA6*}$as the E1 enzyme and UbcH5c as the E2 enzyme. Time points: 0, 2, 5, 10, and 30 min. **b,** Quantification of the Ubch5c~Ub formation. Data are presented as mean ± s.d. from two independent biological experiments. **c**, E1-E2 transthioesterification assays using UBA6^{WT}, UBA1^{WT} or UBA1$^{GH-UBA6*}$ as the E1 enzyme and BIRC6^{UBC} as the E2 enzyme. Time points: 0, 2, 5, 10, and 30 min. **d**, Quantification of the the BIRC6^{UBC}~Ub. Data are presented as mean ± s.d. from two independent biological experiments.

Second, regarding your question about combining UFD and gate helix mutations: consistent with the findings from Nayak et al., simultaneous swapping of both the UFD and SCCH (including gate helix) domains of UBA6 into UBA1 raises the recovery of BIRC6^{UBC}~Ub formation to approximately 40% (**Rev. Fig. 5**). This result supports the idea that additional UFD domain replacement can improve overall activity.

From PMID: 41764162, Nayak et al.

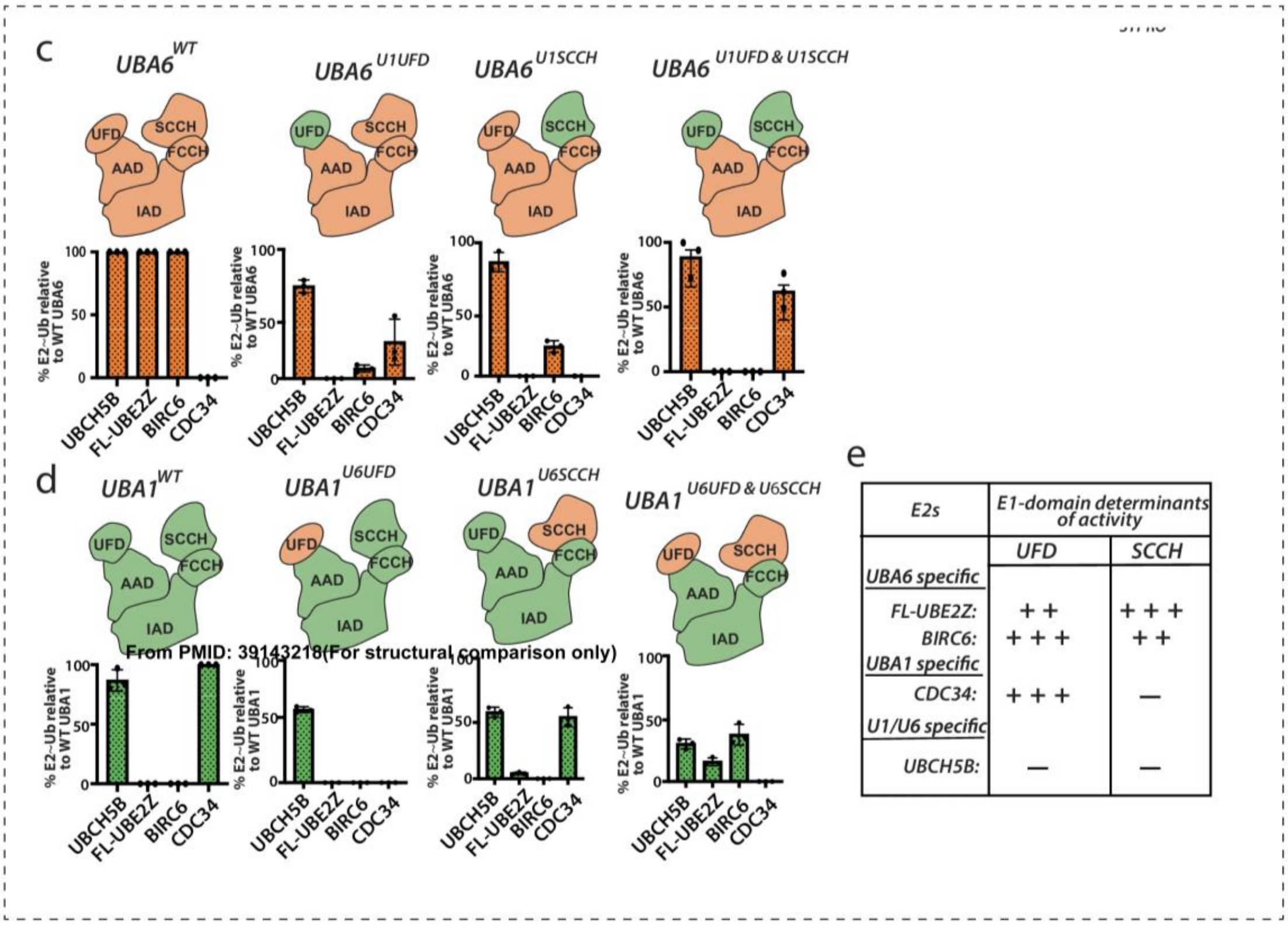

Rev. Fig. 5 | Domain swap experiments of UFD and SCCH domains of UBA1 and UBA6 (from Nayak et al., Nat. Commun. 2026, PMID 41764162).

Collectively, these mutational results support our dual-code model. The UFD domain governs initial partner recruitment and specific binding, while the gate helix within the SCCH domain ensures proper catalytic orientation. Full biochemical compatibility relies on the coordinated action of both domains, which explains the higher activity recovery observed in the dual-domain swap assay.

6. Both reviewers asked about the competition assay, and the authors present additional data in the rebuttal with UBA1 mutants that cannot bind ATP, or cannot undergo thiolation. However, the data has been removed from the manuscript. Is this the intention? The data in Rev. Figure 4 (and 15) is anyway confusing as the quantification of the assay in Rev. Figure 4b actually shows that UBA1 is only slightly inhibiting UBA6-BIRC6 loading and is partially dependent on UBA1's ability to adenylate Ub, i.e. it is competing for ATP in the reaction mixture. This appears to contrast the original data presented by the authors (Rev. Figure 14). Furthermore, the authors state UBA1 and UBA6 bind to BIRC6 with comparable affinities therefore why is a 60-fold excess of UBA1 to UBA6 required to observe 20% inhibition, this would not make sense if both UBA1 and UBA6 have equivalent binding affinities to BIRC6?

Response: We appreciate the reviewer's careful analysis of the competition assays and related datasets. The additional results obtained from UBA1 mutants deficient in ATP binding or thiolation are presented in last rebuttal and have been excluded from the main manuscript, and this removal was deliberate.

We acknowledge that our earlier view of comparable binding affinities between UBA1 and UBA6 for $BIRC6^{UBC}$ conflicts with the competition results, as it is difficult to explain why a 60-fold

molar excess of UBA1 could only yield approximately 20% inhibition under that assumption. This conflict can be clarified using our updated BLI measurements described in previous sections. Our data show that BIRC6^{UBC} binds UBA6-Ub with a steady-state K_D of 2.41 μM, while valid binding curves could not be generated for interactions between BIRC6^{UBC} and UBA1 or UBA1-Ub. This difference in binding capacity accounts for the weak competitive effect, since BIRC6^{UBC} cannot effectively interact with UBA1. We also note the inconsistent outcomes between earlier **Rev. Figure 4/15** and the earlier **Rev. Figure 14**. Assays measuring enzyme competition are easily affected by variations in reaction conditions, which may lead to variable results across experimental batches. Data from UBA1 mutant constructs further suggest that the mild inhibition observed at high UBA1 concentrations is linked to competition for shared ATP and ubiquitin substrates. Consistent with your observation, this effect is partially associated with the Ub adenylation activity of UBA1, rather than direct interference at the E1–E2 interaction interface.

Given that the inhibitory effects observed in these competition assays have limited physiological relevance and do not offer new insights into the core structural gating mechanism of this work, we have removed all competition-related data and mutant results from the revised manuscript. Retaining these datasets may bring unnecessary confusion and shift focus away from our key mechanistic findings. We thank the reviewer for this valuable feedback, which has helped enhance the logical coherence and overall focus of our manuscript.
